# Bio-Enhanced Neoligaments Graft Bearing FE002 Primary Progenitor Tenocytes: Allogeneic Tissue Engineering & Surgical Proofs-of-Concept for Hand Ligament Regenerative Medicine

**DOI:** 10.3390/pharmaceutics15071873

**Published:** 2023-07-03

**Authors:** Annick Jeannerat, Joachim Meuli, Cédric Peneveyre, Sandra Jaccoud, Michèle Chemali, Axelle Thomas, Zhifeng Liao, Philippe Abdel-Sayed, Corinne Scaletta, Nathalie Hirt-Burri, Lee Ann Applegate, Wassim Raffoul, Alexis Laurent

**Affiliations:** 1 Preclinical Research Department, LAM Biotechnologies SA, CH-1066 Epalinges, Switzerland; annick.jeannerat@lambiotechnologies.com (A.J.); cedric.peneveyre@lambiotechnologies.com (C.P.); 2 Plastic and Hand Surgery Service, Lausanne University Hospital, University of Lausanne, CH-1011 Lausanne, Switzerland; joachim.meuli@chuv.ch (J.M.); sandra.jaccoud@chuv.ch (S.J.); michele.chemali@chuv.ch (M.C.); axelle.thomas@chuv.ch (A.T.); zhifeng.liao@unil.ch (Z.L.); philippe.abdel-sayed@chuv.ch (P.A.-S.); corinne.scaletta@chuv.ch (C.S.); nathalie.burri@chuv.ch (N.H.-B.); lee.laurent-applegate@chuv.ch (L.A.A.); 3 Laboratory of Biomechanical Orthopedics, Ecole Polytechnique Fédérale de Lausanne, CH-1015 Lausanne, Switzerland; 4 DLL Bioengineering, STI School of Engineering, Ecole Polytechnique Fédérale de Lausanne, CH-1015 Lausanne, Switzerland; 5 Center for Applied Biotechnology and Molecular Medicine, University of Zurich, CH-8057 Zurich, Switzerland; 6 Oxford OSCAR Suzhou Center, Oxford University, Suzhou 215123, China

**Keywords:** allogeneic cell therapy, clinical protocols, FE002 primary progenitor tenocytes, hand surgery, ligamentoplasty, proof-of-concept, regenerative medicine, tissue engineering, translational development, standardized transplants

## Abstract

Hand tendon/ligament structural ruptures (tears, lacerations) often require surgical reconstruction and grafting, for the restauration of finger mechanical functions. Clinical-grade human primary progenitor tenocytes (FE002 cryopreserved progenitor cell source) have been previously proposed for diversified therapeutic uses within allogeneic tissue engineering and regenerative medicine applications. The aim of this study was to establish bioengineering and surgical proofs-of-concept for an artificial graft (Neoligaments Infinity-Lock 3 device) bearing cultured and viable FE002 primary progenitor tenocytes. Technical optimization and in vitro validation work showed that the combined preparations could be rapidly obtained (dynamic cell seeding of 10^5^ cells/cm of scaffold, 7 days of co-culture). The studied standardized transplants presented homogeneous cellular colonization in vitro (cellular alignment/coating along the scaffold fibers) and other critical functional attributes (tendon extracellular matrix component such as collagen I and aggrecan synthesis/deposition along the scaffold fibers). Notably, major safety- and functionality-related parameters/attributes of the FE002 cells/finished combination products were compiled and set forth (telomerase activity, adhesion and biological coating potentials). A two-part human cadaveric study enabled to establish clinical protocols for hand ligament cell-assisted surgery (ligamento-suspension plasty after trapeziectomy, thumb metacarpo-phalangeal ulnar collateral ligamentoplasty). Importantly, the aggregated experimental results clearly confirmed that functional and clinically usable allogeneic cell-scaffold combination products could be rapidly and robustly prepared for bio-enhanced hand ligament reconstruction. Major advantages of the considered bioengineered graft were discussed in light of existing clinical protocols based on autologous tenocyte transplantation. Overall, this study established proofs-of-concept for the translational development of a functional tissue engineering protocol in allogeneic musculoskeletal regenerative medicine, in view of a pilot clinical trial.

## 1. Introduction

The necessity of functional tendons in the musculoskeletal system and the high incidence of tendon-related disorders are important components of modern socio-economic burdens [1,2,3,4]. High diversity is reported in the etiology of tendon disorders, with possible combined promoting and triggering factors (e.g., intrinsic and extrinsic factors, mechanical overuse). Such diverse and combined factors may potentially lead to tendon ruptures [1,5,6,7]. Then, as the pathological spectrum of tendon disorders is large, various treatment strategies are currently applied and depend on the specific clinical situation [2,3,8,9,10,11,12]. Importantly, symptomatic management of pain and inflammation do not enhance tendon tissue healing capacities, which are inherently poor (i.e., hypocellularity, low vascularization) [1,5,6]. Physiological healing of tendon injuries generally leads to adhesions, scarring, and low overall quality of repair (i.e., mechanically inferior tissues), incurring high morbidity [1,5,13,14,15,16]. Notably, injuries to the Achilles tendons are highly prevalent in sports medicine, where 30% of cases require surgical treatment [5,17]. While partial tendon ruptures may be managed by suturing, volumetric tissue defects require surgical grafting [6,18,19]. Therein, autografting of vestigial tendon tissue is clinically preferred. However, its practical availability is inconsistent and incurs additional morbidity (i.e., related to donor-site surgery). These clinical facts have prompted the development of exogeneous tendon grafts or substitutes (e.g., synthetic matrices, biological constructs) [2,9,20,21,22,23,24,25,26,27,28]. Despite their increased availability and manufacturing process standardization, tendon allografts and xenografts bear an increased risk of tissue inflammation, iatrogenicity, and rejection compared to autografts [3,9,29,30,31,32].

Solid scaffold-based solutions for ligament and tendon repair or replacement are well-defined and commercially available [9,20,24]. An ideal ad hoc scaffold should be biocompatible, show in vivo cell adhesion, cell proliferation/migration, and extracellular matrix deposition (i.e., bio-integration) [4,17,21,22,23,33]. Furthermore, such scaffolds should present good mechanical properties and resist to the physiological strains typically applied to tendons and ligaments [20,21,34]. Therefore, biological (e.g., human or porcine tissues) and synthetic materials have been proposed for tendon surgical reconstruction [9,17,21,22,23,24,25,26]. While biological scaffolds provide a favorable environment for cells, several manufacturing process-related (i.e., decellularization, sterilization) problematics have been reported (e.g., reduced mechanical attributes or pro-inflammatory effects) [19,21,24]. Conversely, artificial tendon grafts can be standardized, tailored to specific applications, and serially produced with a controlled terminal sterilization step while maintaining appropriate critical quality attributes [9,17,35,36,37]. However, their biocompatibility is generally found to be lower compared to biological materials (e.g., formation/release of toxic degradation products) [19,22,24]. Therefore, current efforts are directed toward the supplementation of existing tissue suturing or grafting procedures with appropriate biological components (e.g., growth factors, autologous or allogeneic cells, platelet-rich plasma), to potentially optimize tendon healing [38,39,40,41,42,43,44,45,46].

Notably, growth factors such as platelet-derived growth factor-BB (PDGF-BB) have been preclinically investigated in combination with synthetic scaffolds to promote tendon healing, yielding encouraging results [26,40,41]. As regards the use of platelet-rich plasma (PRP) for tendinopathies, positive symptomatic and functional outcomes have been reported [11,42,47,48]. Recently, various types of therapeutic cell sources were clinically assessed (e.g., mesenchymal stem cells, UVEC, tenocytes) for managing tendon disorders, with positive safety and efficacy outcomes [12,13,49,50,51,52,53]. Among the postulated mechanisms of action of cell therapies, the paracrine modulation of wounded environments by diverse growth factors and cytokines is frequently cited [3,13,38,39]. Of note, autologous cultured tenocyte injections (i.e., Ortho-ATI, Orthocell, Australia) were shown to improve both the function and the MRI tendinopathy scores in chronic lateral epicondylitis at 4.5 years of follow-up [43,52]. In an allogeneic setting, FE002 primary progenitor tenocytes (i.e., clinical grade FE002 progenitor cell source) were proposed as standardized homologous cytotherapeutic materials under the Swiss progenitor cell transplantation program [39,54]. Specifically, FE002 primary progenitor tenocytes were considered for diverse therapeutic tissue engineering purposes or for the optimization of novel injectable medical devices [39,55,56,57,58]. Of note, the FE002 primary progenitor tenocytes of interest (i.e., primary cell type) are diploid cells, which are inherently pre-terminally differentiated and possess stable and robust biological attributes. Such characteristics are maintained within large scale in vitro biotechnological manufacture [39,55]. Importantly, FE002 primary progenitor tenocytes are cytocompatible with diverse implantable materials, are immunologically privileged, and are non-tumorigenic [19,39,54]. Therefore, such primary progenitor cells represent an optimal cellular active substance (i.e., the substance responsible for the activity of a medicine). Off-the-freezer preparation of FE002 progenitor cell-seeded allografts (i.e., using synthetic tendon/ligament scaffolds, decellularized biological tendon matrices, or hyaluronan hydrogels) has previously been reported [19,39,54,56]. Therefore, FE002 primary progenitor tenocytes may be applied in diverse musculoskeletal affections, ranging from volumetric tissue substitution to local pain and inflammation management [39]. Specifically, the combination of an appropriate synthetic scaffold and of FE002 primary progenitor tenocytes for tendon or ligament bioengineering bears the potential of leveraging the desirable attributes of both components [39,56].

Therefore, the general aim of the present study was to establish allogeneic tissue engineering and surgical proofs-of-concept for an artificial tendon/ligament graft bearing cultured FE002 primary progenitor tenocytes. In order to pursue this goal, three specific objectives were defined at the time of designing the presented work, namely:

(1) Experimental verification of the compatibility of the FE002 primary progenitor cells and the Neoligaments scaffold at the cellular and proteomic levels;
(2) Technical devising and experimental validation of optimized manufacturing procedures in order to obtain clinically usable combination products;
(3) Clinical devising and experimental validation of surgical procedures and protocols for hand ligament regenerative medicine with the considered implantable and bio-enhanced combination product.

The retained scaffold (i.e., Infinity-Lock 3 Neoligaments device, non-resorbable woven polyester, Xiros, Leeds, UK) was based on the Leeds-Keio (LK) artificial ligament, which was globally clinically applied in various musculoskeletal indications by several groups since 1982 (i.e., extensive clinical follow-up studies available) [59,60,61,62,63,64,65,66,67]. Designed for passive and gradual integration in patient tissues, the synthetic Infinity-Lock 3 scaffold was incubated with cultured FE002 primary progenitor tenocytes to obtain combination product prototypes [60]. In detail, the objective was to firstly verify the in vitro compatibility of both components, as well as critical and key functional attributes (e.g., cell colonization and extracellular matrix deposition along the scaffold fibers) of the considered combination product, aiming to eventually enhance graft bio-integration in vivo. The second objective was to establish an optimized good manufacturing practice (GMP)-transposable (i.e., adapted for clean room environments) manufacturing process for the combination product, for further translational and clinical applications. The third objective was to validate the applicability of the retained scaffold in two clinical indications of hand ligament surgery within a human cadaveric sub-study, in view of clinical protocol establishment for a pilot clinical trial. Building on the respectively available bodies of knowledge around the considered Neoligaments polymeric scaffold and around the FE002 primary progenitor cell source, tangible data were generated about the considered combinational approach and its applicability in surgical workflows [39,55,56,59,60,61,62,63,64,67,68]. Overall, this study enabled to set forth tangible proofs-of-concept for the translational development of an allogeneic tissue engineering protocol for bio-enhanced hand ligament surgical reconstructive care.

## 2. Materials and Methods

### 2.1. Reagents and Consumables Used for the In Vitro Studies

The reagents and consumables that were used in this study are summarized hereafter, along with the corresponding manufacturers: purified water, PBS buffer, and NaCl 0.9% solutions (Laboratorium Dr. G. Bichsel, Unterseen, Switzerland); high-glucose DMEM cell culture medium, L-glutamine, D-PBS, TrypLE dissociation reagent, penicillin-streptomycin, BCA assay kits, NuPAGE Bis-Tris 4–12% protein gel, MOPS buffer, loading buffer, DTT, antioxidant, page ruler protein ladder, transfer buffer, MTT, antibodies, and 96-well PCR plates (Thermo Fisher Scientific, Waltham, MA, USA); C-Chip Neubauer hemocytometers (NanoEntek, Seoul, Korea); ethanol, methanol, Tween 20, Telomerase activity assay kits, and HCl (Chemie Brunschwig, Basel, Switzerland); Millipore Stericup with 0.22 μm pores, Trypan blue solution, FBS, collagen I from rat tails, and fibronectin (Merck, Darmstadt, Germany); cell culture vessels and plastic assay surfaces (Greiner BioOne, Frickenhausen, Germany and TPP Techno Plastic Products, Trasadingen, Switzerland); RIPA lysis buffer and antibodies (Abcam, Cambridge, UK); protease inhibitor and CellTiter-Glo kits (Promega, Madison, WI, USA); Live-Dead kits and antibodies (Biotium, Fremont, CA, USA); powdered skim milk (Rapilait, Migros, France); saccharose (PanReac AppliChem, Darmstadt, Germany); dextran 40,000 (Pharmacosmos, Wiesbaden, Germany); Lyoprotect cups and Lyoprotect bags (Teclen, Oberpframmern, Germany); nitrocellulose membranes and ECL (Amersham Protran, Cytiva, Marlborough, MA, USA); BSA (PAN Biotech, Aidenbach, Germany).

### 2.2. Instruments and Equipment Used for the In Vitro Studies

For sample analysis, flat bottom 96-well microtitration plates and Eppendorf tubes were purchased from Greiner (Greiner, Frickenhausen, Germany). Component weighing was performed on a laboratory scale (Ohaus, Parsippany, NJ, USA). Sample centrifugation was performed on a Rotina 420R centrifuge (Hettich, Tuttlingen, Germany). Dynamic cell seeding of scaffolds was performed in a Roto-Therm Plus agitator (Benchmark Scientific, Sayreville, NJ, USA). SDS-Page analyses were performed using a Mini Gel Tank and PowerEase 90W (Thermo Fisher Scientific, Waltham, MA, USA). Gel imaging in white light or in chemiluminescence was performed on a Uvitec Mini HD9 gel imager (Cleaver Scientific, Rugby, UK). Colorimetric and luminescence measurements were performed on a Varioskan LUX multimode plate reader (Thermo Fisher Scientific, Waltham, MA, USA). Immunohistochemistry and Live-Dead imaging were performed on an inverted IX81 fluorescence microscope (Olympus, Tokyo, Japan). Telomerase activity PCR analyses were run on a StepOne Real-time PCR Systems instrument (Thermo Fisher Scientific, Waltham, MA, USA). Sample lyophilization was performed in a LyoBeta Mini freeze-dryer (Telstar, Terrassa, Spain). Sample terminal sterilization by γ-irradiation was performed by Ionisos, Dagneux, France.

### 2.3. Surgical and Grafting Materials Used for the Ex Vivo Studies

For the needs of the in vitro and ex vivo work (i.e., human cadaveric model), the artificial ligaments and surgical instruments (i.e., medical devices) were provided by Xiros, Leeds, UK. The provided artificial ligaments (i.e., woven polyester tapes) were 3 mm-wide Neoligaments Infinity-Lock 3 devices (i.e., two different manufacturing process-related options) and Jewel ACL devices. The provided surgical instruments were from a FlexPasser Tendon Retrieval Kit. Standard hand surgery instruments, materials, and consumables were provided by the Plastic and Hand Surgery Service at the CHUV Lausanne University Hospital, Lausanne, Switzerland.

### 2.4. FE002 Primary Progenitor Tenocyte Cell Sourcing and In Vitro Cellular Active Substance Lot Manufacture

The FE002 primary progenitor tenocyte source used for the in vitro experiments of this study consisted of banked primary human diploid cells (i.e., clinical grade FE002 progenitor cell source). The considered FE002 progenitor cells were procured and produced under the Swiss progenitor cell transplantation program and were made available for the present study as cryopreserved stocks, as previously described elsewhere [55]. All of the FE002 primary progenitor tenocytes used in the present study were characterized by in vitro passage levels of 6–8. Briefly, frozen vials of FE002 cells were used as cellular seeding materials for the in vitro expansions necessary to generate the cellular active substance lots. Rapid thawing of the vials was performed and the cells were suspended in warmed complete cell culture medium (i.e., DMEM; 10% *v*/*v* FBS; 5.97 mM L-glutamine). The cell suspension titers and the relative cellular viability were determined by hemocytometer counts using Trypan blue exclusion dye. The cell suspensions were then used to homogeneously seed an appropriate amount of culture vessels using a 1.5 × 10^3^ cells/cm^2^ relative seeding density. The seeded cell culture vessels were incubated at 37 °C in humidified incubators under 5% *v*/*v* CO_2_. Cellular adherence checks were performed the following day and the cell culture medium was exchanged twice weekly. The cell culture vessels were examined at each medium exchange procedure, for confirmation of cell proliferation, adequate proliferative cellular morphology maintenance (i.e., characteristic spindle-shape morphology), and absence of observable extraneous agent contamination. Once optimal cell monolayer confluency was attained (i.e., >95%), the cells were harvested. The cell suspension titers and the relative cellular viability were determined. These cell suspensions were defined as a “fresh cellular active substance lot”, for cell type characterization/qualification studies or for seeding onto synthetic Neoligaments scaffolds for finished combination product preparation. Alternatively, “cryopreserved cellular active substance lots” were used for synthetic scaffold seeding. Therefore, the cell suspensions were used directly after thawing, following the cell enumeration control step. For the needs of the present study, a cellular active substance lot was therefore composed of one of the following:

- “Fresh cellular active substance lot”: Suspension of FE002 primary progenitor tenocytes (i.e., homogeneous cellular suspension in an appropriate solvent/medium, e.g., DMEM-based medium), expanded once in vitro in monolayer culture, harvested, and controlled before further use;
- “Cryopreserved cellular active substance lot”: Cryopreserved FE002 primary progenitor tenocytes (i.e., homogeneous cellular suspension in an appropriate solvent/medium), conditioned in cryopreservation vials, extemporaneously thawed/rinsed, and controlled before further use.

### 2.5. Patient Primary Tenocyte Cell Sourcing and In Vitro Cell Lot Manufacture

The patient primary tenocyte source used for the in vitro experiments of this study consisted of banked primary human diploid cells (i.e., Ad001-Ten standardized cell source). All of the patient tenocytes used in the present study were characterized by in vitro passage levels of 6–8. Patient tenocytes were obtained from the Biobank of the Department of Musculoskeletal Medicine in the CHUV Lausanne University Hospital, Lausanne, Switzerland, in the form of cryopreserved vial lots. Biological material sourcing and primary cell type establishment had been performed from a hand digit flexor tendon (i.e., medical waste) of a 74-year-old female patient. The patient tenocytes were manufactured using serial in vitro cellular expansion rounds, following the same technical specifications as the considered FE002 primary progenitor tenocytes. The patient tenocytes were used for the in vitro experiments of this study, either in “fresh cellular active substance lot” form or in “cryopreserved cellular active substance lot” form.

### 2.6. FE002 Primary Progenitor Tenocyte Cellular Active Substance Characterization Assays

The assays presented hereafter were performed in order to complement the existing body of knowledge around FE002 primary progenitor tenocytes, as previously reported for this cellular active substance or cellular starting material [39,55,56,57,58].

#### 2.6.1. Quality and Potency-Related Characterization and Qualification Assays

Firstly, mass spectrometry proteomic characterization assays were performed to gain insights into the major constituents of the cellular active substance of interest. The comparative proteomic analyses were performed using quantitative mass spectrometry, following the protocol reported by Jeannerat et al. (2021) [55]. Briefly, FE002 primary progenitor tenocytes and patient primary tenocyte samples were lysed and the protein contents were digested using an adapted filter-aided sample preparation (FASP) protocol. The obtained peptides were labelled and were analyzed by LC-MS/MS. Mass spectrometry proteomic data were deposited at the ProteomeXchange Consortium (http://www.proteomexchange.org/, accessed on 23 May 2023) via the PRIDE partner repository with the dataset identifier PXD028359 [55]. Specific data processing then enabled to obtain relative protein levels in the various samples, for comparative consideration. Relatively abundant proteins were retained for further analysis within both experimental groups (i.e., progenitor or patient tenocytes) and were compared to literature reference sources [69].

Secondly, in order to verify that the considered FE002 cellular active substance is capable of adhering on tendon extracellular matrix (ECM) components, an in vitro cellular adhesion assay was performed. Briefly, 96-well ELISA microplates were coated overnight with 50 µg/mL collagen I, 100% FBS, or 5 µg/mL fibronectin. The microplates were then washed with PBS and blocked for 1 h using PBS with 1% BSA. Freshly harvested FE002 primary progenitor tenocytes or primary patient tenocytes were suspended in DMEM with 1% BSA at a final concentration of 2 × 10^5^ cells/mL. Volumes of 100 µL of cell suspension were dispensed in each well and the microplates were incubated for 1 h at 37 °C. The plates were then gently washed using DMEM with 1% BSA and pictures of the wells were taken on an inverted microscope, for the comparative assessment of cellular adhesion.

#### 2.6.2. Quality and Safety-Related Characterization and Qualification Assays

Firstly, a β-galactosidase staining assay was performed, in order to confirm that the considered FE002 primary progenitor tenocytes were a cell type (i.e., and not a cell line) with a finite in vitro lifespan and reached senescence in culture at high passage levels under standard conditions. Cells at in vitro passage levels known to be characterized by significantly reduced proliferation capacities in the retained manufacturing system (i.e., passage levels > 8) were used for the assays. Briefly, FE002 primary progenitor tenocytes were seeded in T25 cell culture flasks at 1.5 × 10^3^ cells/cm^2^ and were expanded until reaching 70% confluency. The cells were then fixed for 5 min in 10 mL of fixation solution containing 1.85% formaldehyde with 0.2% glutaraldehyde. The cells were then rinsed twice using PBS. The cells were stained overnight at 37 °C with a SA-β-gal staining solution containing 0.1% X-gal, 5 mM potassium ferrocyanide, 5 mM potassium ferricyanide, 150 mM NaCl, and 2 mM MgCl_2_ in a 40 mM citric acid/sodium phosphate solution at pH 6.0. The cells were washed twice with PBS and once with DMSO to remove the staining solution. The presence of β-galactosidase-positive (i.e., stained in blue) cells was assessed microscopically. Staining for the senescence marker β-galactosidase was performed between in vitro passage levels 8 and 10.

Secondly, a telomerase activity assay was performed using the Telomerase activity quantification qPCR assay kit in order to confirm the non-tumorigenic potential of the considered FE002 primary progenitor tenocytes (i.e., absence of significant levels of telomerase activity). Telomerase activity quantification was performed using frozen cellular dry pellets (i.e., passage level 8 for the FE002 cells). HeLa cells were obtained from the Musculoskeletal Research Unit at the University of Zurich (Zurich, Switzerland) and were used as positive controls in the telomerase assay. For cell lysate preparation, cellular dry pellets (i.e., 2–5 × 10^6^ cells/tube) were retrieved from −80 °C storage. Cells lysis was performed by mixing the cells with 20 µL of lysis buffer (i.e., supplemented with PMSF and β-mercaptoethanol before use) per million cells before a 30-min incubation period on ice. The samples were then centrifuged at 12,000 rpm for 20 min at 4 °C. The supernatants were transferred to new Eppendorf tubes. For the telomerase-mediated reaction, 0.5 µL of sample, 4 µL of 5× telomerase reaction buffer, and 15.5 µL of nuclease-free water were mixed and incubated for 3 h at 37 °C. The reaction was quenched by heating the samples for 10 min at 85 °C. The samples were centrifuged at 1500× *g* for 10 s and were stored on ice. The wells necessary for the qPCR reactions were prepared in triplicate in qPCR plates by mixing 1 µL of the prepared sample, 2 µL of primers, 10 µL of TaqGreen qPCR master mix, and 7 µL of nuclease-free water. The qPCR plates were sealed and centrifuged at 1500× *g* for 15 s. The samples were run on a StepOne Real-time PCR Systems instrument. The qPCR run conditions comprised an initial denaturation step of 10 min at 95 °C and 36 amplification cycles (i.e., denaturation over 20 s at 95 °C; annealing over 20 s at 52 °C; extension over 45 s at 72 °C). Samples with a Ct >33 in value were considered as being negative. Relative telomerase activity quantification between two samples was based on the 2^−ΔCt^ calculation method.

### 2.7. FE002 Primary Progenitor Tenocyte-Seeded Neoligaments Graft: Manufacturing Process Development Phase

#### 2.7.1. Progenitor Cell Seeding and Combination Product Incubation Processes

In order to establish an initial proof-of-concept for scaffold-based FE002 progenitor cell-bearing construct bioengineering, conservative parameters and technical specifications were used. Two synthetic scaffold cell seeding strategies were experimentally investigated. Firstly, a static cell seeding protocol was used. Neoligaments Infinity-Lock 3 scaffold pieces of 1 cm in length were placed in 12-well microplates and volumes of 250 µL of cell suspension (i.e., fresh FE002 cellular active substance in complete cell culture medium, at various final cellular concentrations ranging from 25 × 10^3^ to 10^5^ cells/scaffold) were homogeneously dispensed in order to completely soak the scaffolds. Cell recovery quality control plates (i.e., 6-well microplates) were prepared at that time, with the same technical specifications as for FE002 primary progenitor tenocyte manufacturing activities. The sample-bearing microplates were incubated for 1 h at 37 °C, to enable initial cellular attachment. Then, volumes of 500 µL of complete cell culture medium (i.e., with 1% penicillin-streptomycin) were dispensed in each well and the microplates were incubated again. The cell-seeded scaffolds were maintained in culture for 14 days with medium exchange procedures performed twice per week before endpoint harvest.

Secondly, a dynamic cell seeding protocol was used. Fresh 1 cm-pieces of Infinity-Lock 3 scaffold were placed in 2 mL Eppendorf tubes and 1 mL of cell suspension (i.e., fresh FE002 cellular active substance in complete cell culture medium, at various final cellular concentrations) was dispensed in each tube. Cell recovery quality control plates were prepared at that time, as described hereabove. The tubes were incubated at 37 °C overnight under rotational agitation (i.e., 13 rpm, monoaxial rotation), to enable initial cellular attachment. Then, the scaffolds were transferred to 15 mL centrifugation tubes and were covered with 1 mL of fresh complete cell culture medium (i.e., with 1% penicillin-streptomycin). The volumes of 1 mL of spent culture medium (i.e., used for overnight dynamic cell seeding) were conserved and were used to prepare secondary cell recovery quality control plates. The cell-seeded scaffolds were maintained in culture for 14 days with medium exchange procedures performed twice per week before endpoint harvest. The dynamic cell seeding protocol was then repeated using fresh patient tenocyte suspensions (i.e., in “fresh cellular active substance lot” form), for cytocompatibility comparison with the FE002 primary progenitor tenocytes and for validation of the ability of patient cells to adhere to the synthetic scaffolds. The dynamic cell seeding protocol was then repeated again using two sub-types of Infinity-Lock 3 scaffolds (i.e., plasma-treated and non-plasma treated scaffolds), for assessment of the impact of the additional scaffold processing step on cytocompatibility and cellular functionality parameters.

For endpoint cell-seeded construct harvesting, the spent cell culture medium was removed from the 3D culture vessels. The constructs were rinsed thrice by immersion in warm PBS and were made available for further in vitro studies or for conditioning in finished product transport medium. At each step of the construct incubation process, the recovery quality control microplates were assessed (i.e., cell adherence on the culture surface and low cellular detachment, appropriate adherent cell morphology, positive cell confluency evolution, appropriate cellular metabolic activity) as part of in-process controls (IPC). Appropriate post-process controls (PPC) were implemented as appropriate.

#### 2.7.2. CellTiter-Glo Assay for Endpoint Assessment of Cellular Metabolic Activity within the Combination Products

A CellTiter-Glo assay was used to assess the metabolic activity of the cells on the constructs following the incubation period. Briefly, the constructs were harvested, rinsed, and placed in 24-well microplates. Volumes of 200 µL of PBS and 200 µL of CellTiter-Glo reagent were dispensed in each well. Micropipette tips were then used to lightly crush the immerged constructs. The plates were incubated for 10 min at ambient temperature. Finally, 200 µL of supernatant were isolated from each well and luminescence was measured.

#### 2.7.3. MTT Assay for Endpoint Assessment of Cellular Metabolic Activity and Cell Distribution throughout the Combination Products

An MTT assay was used to assess the cytocompatibility of the FE002 primary progenitor tenocytes and the Neoligaments scaffolds (i.e., Infinity-Lock 3 and Jewel ACL devices). Specifically, the MTT assay was used to confirm (i) the adherence of the cells throughout the scaffolds, (ii) the maintenance of cellular metabolic activity on the scaffolds, and (iii) the quality of cellular colonization of the scaffolds (i.e., homogeneous repartition of the cells on the available fiber surfaces). Furthermore, MTT assays were performed at various timepoints (i.e., between 1 day and 3 weeks) of the incubation phase of the cell-seeded constructs and enabled to assess the 3D in vitro cellular proliferation and migration on the scaffolds. For endpoint analysis, the constructs were harvested and incubated for 2 h at 37 °C in a 5 mg/mL MTT solution. Following rinsing of the constructs, photographic imaging was performed. For the further quantification of the MTT dye in the considered constructs, the dye was extracted using a 0.04 N HCl ethanolic solubilization solution. Then, volumes of 150 µL of the MTT extracts were transferred to 96-well microplates. Sample absorbance values were determined at a wavelength of 570 nm.

#### 2.7.4. Live-Dead Assay for Endpoint Assessment of Cellular Viability and Distribution throughout the Combination Products

A Live-Dead assay (i.e., viability and toxicity assay kit) was used to assess cellular viability, cellular adhesion, and cellular morphology directly on the constructs, according to the manufacturer’s instructions. Briefly, the Live-Dead staining solution was prepared by mixing 10 mL of D-PBS, 5 µL calcein, and 20 µL ethidium homodimer III. The cell-seeded scaffolds were harvested, rinsed with D-PBS, and incubated with the Live-Dead staining solution for 30 min at ambient temperature. The samples were then washed to remove any excess reagents and were imaged on an Olympus IX81 microscope using the appropriate channels.

#### 2.7.5. Western Blotting for Endpoint Assessment of Extracellular Matrix Component Synthesis and Deposition within the Combination Products

Western blotting analysis was used in order to assess the synthesis and deposition of selected tendon-related ECM proteins (e.g., collagen I, decorin) within the incubated constructs. Briefly, the cell-seeded scaffolds were harvested, washed with PBS, and incubated for 15 min on ice in 300 µL of RIPA lysis buffer supplemented with protease inhibitors. The samples were centrifuged at 3000× *g* for 5 min at 4 °C and the supernatants were stored at −20 °C until analysis. The samples were separated by electrophoresis on NuPAGE 4–12% Bis-tris polyacrylamide gels before being transferred onto nitrocellulose membranes. The membranes were blocked in 0.05% PBS-Tween 20 supplemented with 4% skimmed milk for 15 min at ambient temperature and were then incubated overnight at 4 °C in the primary antibody solution (i.e., anti-collagen I, anti-decorin, or anti-actin). The following day, the membranes were washed in PBS-Tween 20 buffer and were incubated for 1 h at ambient temperature in the corresponding HRP-secondary antibody. Revelation was performed using the ECL Prime chemiluminescence detection system. For all of the presented Western blotting assays, the following antibodies were used:

- Primary anti-collagen I antibody: Abcam Ref. N°ab34710 (1:1000 dilution)
- Primary anti-decorin antibody: Abcam Ref. N°ab277636 (1:1000 dilution)
- Primary anti-actin antibody: Thermo Fisher Ref. N°PA1-21167 (1:200 dilution)
- Secondary anti-rabbit HRP antibody: Biotium Ref. N°20403 (1:2000 dilution)
- Secondary anti-mouse HRP antibody: Biotium Ref. N°20401 (1:2000 dilution)

#### 2.7.6. Immunofluorescence Imaging for Endpoint Assessment of Extracellular Matrix Component Synthesis and Deposition throughout the Combination Products

Direct immunofluorescence staining was used to assess cellular adhesion, cellular morphology, and tendon-related extracellular matrix protein (e.g., collagen I, aggrecan, decorin) deposition throughout the incubated constructs. Briefly, the cell-seeded scaffolds were harvested, washed with PBS, and fixed overnight in PAF at 4 °C. An antigen retrieval step was then performed for 10 min at 37 °C in an antigen retrieval buffered solution (i.e., 0.05 M Tris-HCl; 0.1% CaCl_2_; 0.15 M NaCl) supplemented with 2 mg/mL hyaluronidase. Then, the samples were blocked for 1 h at ambient temperature in a 0.05% PBS-Tween 20 solution supplemented with 1% BSA. The samples were incubated overnight at 4 °C in a primary antibody solution (i.e., phalloidin-iFluor594, anti-collagen I, anti-aggrecan). Except for phalloidin staining, revelation was eventually performed by incubating the constructs in the corresponding secondary antibodies coupled to Alexa Fluor 488. The samples were imaged on an Olympus IX81 microscope. For all of the presented immunofluorescence assays, the following antibodies were used:

- Primary anti-phalloidin-iFluor594 antibody: Abcam Ref. N°ab176757 (1:1000 dilution)
- Primary anti-decorin antibody: Abcam Ref. N°ab175404 (1:100 dilution)
- Primary anti-collagen I antibody: Abcam Ref. N°ab138492 (1:100 dilution)
- Primary anti-aggrecan antibody: Abcam Ref. N°ab3778 (1:100 dilution)
- Primary anti-tenomodulin antibody: Thermo Fisher Ref. N°PA5-112767 (1:100 dilution)
- Rabbit IgG isotype control antibody: Abcam Ref. N°ab172730 (1:100 dilution)
- Mouse isotype control antibody: Abcam Ref. N°ab170190 (1:100 dilution)
- Secondary anti-rabbit Alexa 488 antibody: Abcam Ref. N°ab150081 (1:250 dilution)
- Secondary anti-mouse Alexa 488 antibody: Abcam Ref. N°ab150113 (1:250 dilution)

### 2.8. FE002 Primary Progenitor Tenocyte-Seeded Neoligaments Graft: Manufacturing Process Optimization Phase

#### 2.8.1. Optimized Cell Seeding and Combination Product Incubation Processes

In order to establish a combination product manufacturing process characterized by enhanced scalability and ease of transposition to GMP manufacturing settings, several modifications to the initial technical specifications were implemented. The main objective at this point was to reduce the overall manufacturing period for the cell-seeded constructs and to separate the cellular active substance manufacturing phase from the combination product manufacturing phase, while conserving the endpoint quality attributes of the constructs. Therefore, high cell seeding densities were used (i.e., 10^5^ cells/cm of scaffold) and the construct incubation period was reduced from 14 days to 6 ± 2 days. Endpoint characterization assays were performed (i.e., MTT, Live-Dead, immunofluorescence) to comparatively assess the quality attributes of the combination products in both experimental conditions (i.e., conservative vs. optimized manufacturing workflow). The results of these assessments were summarized in ad hoc parametric grading tables.

#### 2.8.2. Pilot Assessment of Combination Product Lyophilization & Sterilization Processes

In order to initiate a preliminary evaluation for the feasibility of obtaining temperature-stable and terminally-sterilized FE002 primary progenitor tenocyte-seeded constructs, pilot lyophilization and sterilization studies were performed. Firstly, cell-seeded constructs were prepared using fresh FE002 progenitor cellular active substance materials with a 3-week incubation period. Following endpoint harvest, the constructs were immerged in lyopreservation solution composed of 8% saccharose and 2% dextran 40,000 in diluted PBS buffer. The samples were initially frozen at −20 °C. Following loading in the freeze-dryer, annealing was performed between −20 °C and −30 °C. Primary drying was performed over 48 h under a partial vacuum of 0.08 mbar and with a shelf temperature of −22 °C. Secondary drying was performed over 14 h under a partial vacuum of 0.008 mbar and with a shelf temperature of 25 °C. The resulting freeze-dried samples were then conditioned in airtight boxes and were stored at 4 °C until further use. A sublot of samples was processed by ^60^Co gamma irradiation at a dose of 25–30 kGy. For comparative sample analysis, the contents of the boxes were rehydrated with the appropriate amount of distilled water and were left to soak for 5 min. Endpoint characterization assays were performed (i.e., Live-Dead, immunofluorescence imaging) in order to assess the quality attributes in both of the experimental conditions (i.e., lyophilized samples and lyophilized/irradiated samples). The results of these assessments were compared to those obtained on freshly harvested constructs and were summarized in ad hoc parametric grading tables.

### 2.9. Human Ex Vivo Surgical Study for Clinical Protocol Establishment in Hand Ligament Regenerative Medicine

#### 2.9.1. Ex Vivo Human Anatomy Material Procurement and Surgical Processing

For the needs of the ex vivo part of the study, human anatomical body limbs were provided by the Unit of Anatomy and Morphology of the University of Lausanne (Lausanne, Switzerland). All of the ex vivo work was performed on the premises of the Unit of Anatomy and Morphology of the University of Lausanne. The retained ex vivo anatomical model consisted of a left arm from an elderly female patient, sectioned mid-humerus. The arm was frozen but was not chemically preserved before the study. The arm was thawed and was stored at 4 °C until use in the study. In order to optimize the use of human cadaveric samples, the arm was used in a perforator flaps training after completion of this study. To this goal, intra-arterial infusion with 60 mL of commercial latex milk mixed with green acrylic colorant was performed 24 h before the ex vivo study, in order to optimally visualize the microvascular structures. At the end of both studies, all biological materials were disposed of following the applicable regulations and waste management workflows within the Unit of Anatomy and Morphology of the University of Lausanne.

#### 2.9.2. Ex Vivo Neoligaments Graft Implantation Procedure for Ligamento-Suspension Plasty after Trapeziectomy

For the needs of the procedure, the arm was prepared and was placed through an operating field. An initial incision was performed on the dorsal aspect of the trapeziometacarpal joint. Subcutaneous dissection followed, preserving the dorsal branch of the radial nerve. After opening of the articular capsule, the trapezium was fully removed and the flexor carpi radialis (FCR) tendon was exposed. The FlexPasser device was then used to reach under the FCR and to set the ad hoc polymeric sheath in place. Using the sheath, the Infinity-Lock 3 device was passed under the FCR. Excess Infinity-Lock 3 materials were excised and both ends of the device were fixed using osteosutures with Supramid 3-0 (B. Braun, Melsungen, Germany) threads on the basis of the metacarpal bone. Sufficient tension was set to achieve an effective suspension of the thumb upon testing.

#### 2.9.3. Ex Vivo Neoligaments Graft Implantation Procedure for Thumb Metacarpo-Phalangeal Ulnar Collateral Ligamentoplasty

For the needs of the procedure, an initial incision was performed to gain access to the ulnar collateral ligament (UCL). The UCL was sectioned and a 1 cm-portion of the ligament was removed in order to artificially create the equivalent of a complete rupture. Testing of the articulation confirmed an excessive laxity following the section. The Infinity-Lock 3 device was installed and was sutured in place distally first, using Supramid 3-0. Excess Infinity-Lock 3 materials were excised and the free end of the device was sutured proximally using the same thread. Testing of the articulation confirmed that the lateral stability of the joint was restored. If the remaining portions of the artificial ligament were to be insufficient for direct suturing, direct fixation into the bone could be performed using an anchor such as Micro-Mitek (DePuys Synthes, Comté de Bristol, MA, USA). The artificial grafts showed some filamentory fragmentation after being cut. This was assessed as being similar to what can be clinically observed in native tendons and did not impact the stability of the construct.

### 2.10. Statistical Analysis of the Data and Presentation of the Results

For the statistical comparison of average values from two sets of data, a paired Student’s *t*-test was applied, following appropriate evaluation of the normal distribution of data, wherein a *p*-value < 0.05 was retained as a base for statistical significance determination. The calculations and data presentation were performed using Microsoft Excel (Microsoft Corporation, Redmond, WA, USA), Microsoft PowerPoint, and GraphPad Prism version 8.0.2 (GraphPad Software, San Diego, CA, USA).

## 3. Results

### 3.1. FE002 Primary Progenitor Tenocytes Possess Quality and Safety Attributes Compatible with Translational and Clinical Musculoskeletal Tissue Engineering

In order to confirm and to further document the quality- and safety-related attributes of the considered FE002 primary progenitor tenocytes, several cellular active substance characterization and qualification studies were performed in vitro. For facilitated reading of the results, a general overview of the design of the study, presenting the major phases, is presented in Figures S1A and S1B. Firstly, a comparative proteomic analysis revealed that the considered FE002 primary progenitor tenocytes contained major proteinic constituents known to compose native human tendons (e.g., ECM proteoglycans, collagens, ECM glycoproteins), which were also found in the considered patient primary tenocyte group (Table 1).

Secondly, an in vitro cellular adhesion assay was performed using patient primary tenocytes and FE002 primary progenitor tenocytes, to comparatively assess their respective adhesion potentials on ubiquitous ECM components (e.g., collagen I, fibronectin, i.e., found in abundant amounts in tendinous tissues). The results indicated that both of the considered primary cell types were capable of similar and rapid adhesive behaviors on collagen I-, fibronectin-, and FBS-coated surfaces (Figure S2). Of note, FBS is known to contain vitronectin, which is among the molecules most probably responsible for the considered cellular adhesive properties. Thirdly, a β-galactosidase staining assay confirmed that FE002 primary progenitor tenocytes reached senescence at high in vitro passage levels (e.g., passage level N°10), thereby confirming the finite nature of the cell type’s lifespan (Figure S3). Finally, a comparative telomerase activity quantification assay enabled to confirm that FE002 primary progenitor tenocytes possess telomerase activity levels which are two orders of decimal magnitude below those of known tumoral cell lines (i.e., HeLa cells, Table S1). Generally, the obtained in vitro original data complemented the previously published biological characteristics/attributes of FE002 primary progenitor tenocytes [39,55]. Overall, the obtained data enabled to set forth important quality- and functionality-related attributes of FE002 primary progenitor tenocytes (i.e., capacity to adhere to known components of tendons and ligaments) and important safety-related attributes (i.e., low propensity for presenting tumorigenic behaviors) of this cellular active substance.

### 3.2. Allogeneic FE002 Primary Progenitor Tenocytes May Be Rapidly Combined with Neoligaments Devices to Form Biologically-Enhanced Grafts

**Figure 1:**
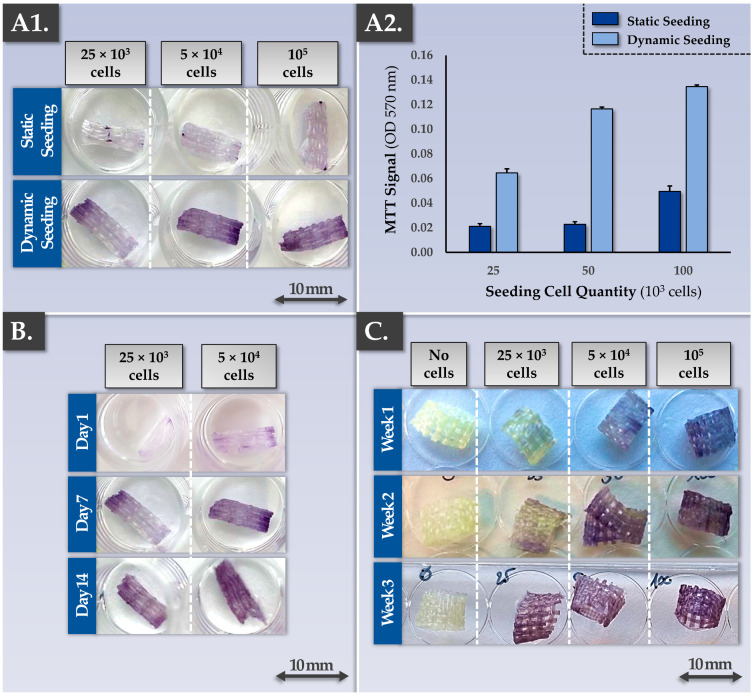
Results of biocompatibility and tissue engineering process development studies aiming to establish the cell seeding density, the cell seeding type (i.e., static or dynamic cell seeding), and the combination product incubation period. The results outlined that in optimal conditions, the FE002 progenitor cells adhere on the scaffolds, are capable of 3D proliferation, and stay metabolically active (i.e., positive MTT readout). (**A1**,**A2**) Dynamic seeding of FE002 primary progenitor tenocytes on the Infinity-Lock 3 scaffolds resulted in superior colonization (i.e., increased efficiency) compared to static cell seeding, as assessed by MTT staining (i.e., cell viability and localization on the scaffold). A dose-dependent relationship was evidenced between the cell seeding density and the scaffold colonization capacity. Imaging was performed 7 days after cell seeding of plasma-treated scaffolds. (**B**) Results indicated that the dynamically seeded FE002 cells were capable of proliferation on the scaffolds. An assessment of various combination product incubation periods revealed that significant scaffold colonization and homogeneous cellular proliferation were attained with a dose of 5 × 10^4^ cells/scaffold at the 7-day and 14-day timepoints. The observed significant increase in MTT signals was confirmed by quantitative analysis (data not shown). (**C**) Similar results of scaffold colonization by FE002 cells were obtained with Jewel ACL scaffolds (i.e., with dynamic cell seeding), as assessed by MTT staining. These results indicated that such scaffolds could potentially be used with a high degree of versatility for musculoskeletal bioengineering, depending on the anatomical location of the affection. Overall, the use of higher cell seeding densities enables reaching of high cell quantities within the combination products in shortened incubation time-periods. OD, optic density.

The sound development of a cell-scaffold finished combination product implies that the chosen scaffold should be perfectly biocompatible with the therapeutic cellular active substance of interest. Specifically, the cells need to be able to bind to the scaffold’s fibers/surfaces and remain viable up until the time of finished product clinical administration. In addition, the cell seeding process must be efficient, to reduce the proportion of non-binding cells. The synthetic material composing the Neoligaments Infinity-Lock 3 scaffold is known to passively allow cell and tissue ingrowth following clinical application. Therefore, the initial focus point of the present study, in view of establishing allogeneic musculoskeletal tissue engineering protocols, consisted in the validation of cytocompatibility aspects between Neoligaments scaffolds and FE002 primary progenitor tenocytes. For material rationalization purposes, the in vitro studies were performed on Infinity-Lock 3 or Jewel ACL scaffold subunits of 1 cm, obtained by fractionation of whole device units. Various cell seeding strategies were investigated, using two cell seeding modalities (i.e., static vs. dynamic), various cell seeding relative doses, and various construct incubation time-periods. The results confirmed the cytocompatibility between the considered Neoligaments scaffolds and the FE002 primary progenitor tenocytes. Furthermore, significant differences between the two cell seeding strategies were evidenced, favoring the exclusive subsequent use of dynamic cell seeding protocols for the in vitro assays of the study (Figure 1(A1,A2)).

Specifically, after cell seeding and construct incubation, MTT staining and the subsequent MTT dye quantification firstly demonstrated the presence of metabolically active cells binding to the scaffolds. Secondly, while the cell seeding density had a significant impact on scaffold colonization capacity by the cells at early construct incubation time-points, samples at late time-points (i.e., two or three weeks of incubation) displayed comparable cellular colonization (Figure 1B,C). These results generally indicated that the Infinity-Lock 3 and Jewel ACL scaffolds were comparable in terms of wettability/cytocompatibility and that the FE002 primary progenitor tenocytes were capable of homogeneous 3D proliferation throughout both scaffolds (Figure 1). Furthermore, it was shown that a dynamic cell seeding protocol was required to enable significant cellular adhesion to the scaffold fibers and that some sort of saturation occurred after homogeneous scaffold colonization was achieved by the cells (Figure 1).

The obtained cytocompatibility data gathered using the MTT readout was confirmed by the Live-Dead readout, showing the presence of adherent and viable cells along the scaffold fibers following incubation (Figure S4). Scaffold autofluorescence had been previously experimentally excluded. In endpoint, most of the present FE002 primary progenitor tenocytes were assessed as being alive (i.e., green staining), while only a few dead cells (i.e., red staining) could be observed on the scaffolds. Furthermore, Live-Dead cell staining allowed to specifically observe the spreading and alignment of the cells along the scaffold fibers (Figure S4). Overall, while the MTT readout enabled the rapid global evaluation of cellular adhesion, scaffold colonization quality, and cellular metabolic activity maintenance, the Live-Dead readout provided complementary information (i.e., relative viable cellular proportion, cell conformation along the fibers). Finally, additional potency-related data were gathered in endpoint on the Infinity-Lock 3 scaffolds bearing FE002 primary progenitor tenocytes, by using immunofluorescence readouts (Figure S5). These results confirmed the presence and the structural organization along the scaffold fibers of ECM components which are naturally present in tendons (i.e., decorin, tenomodulin, aggrecan, phalloidin), confirming the ability of the cultured FE002 primary progenitor tenocytes to deploy their functions in appropriate 3D environments (Figure S5).

**Figure 2:**
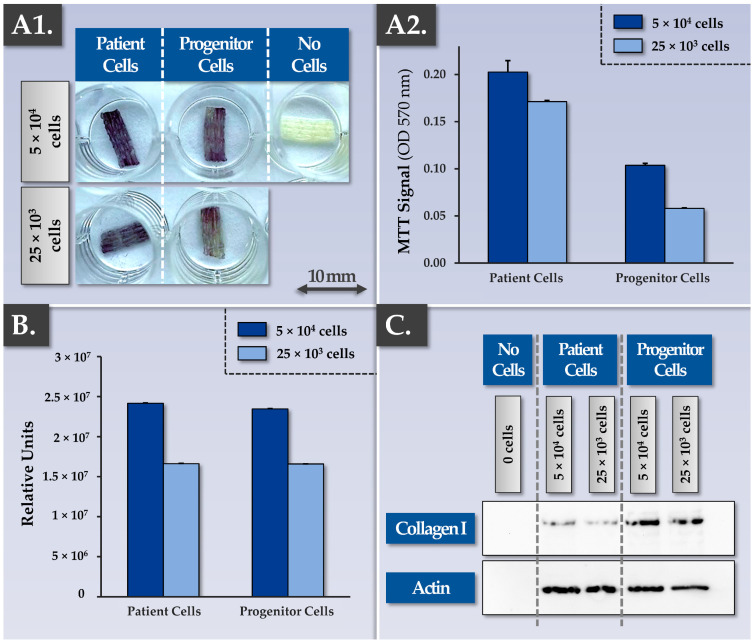
Results of comparative assessments for Infinity-Lock 3 scaffold colonization capacities by patient primary tenocytes and by FE002 primary progenitor tenocytes. (**A1**) Both cell types were shown to be able to bind and proliferate throughout the scaffolds. Imaging was performed 13 days after dynamic cell seeding of plasma-treated scaffolds. (**A2**) Patient primary tenocytes were shown to possess similar scaffold colonization capacities compared to FE002 primary progenitor tenocytes, as assessed by MTT staining. A dose-dependent relationship was evidenced between the cell seeding density and scaffold colonization capacity. Superior metabolic activity levels were recorded in the patient tenocyte group. Analyses were performed 6 days after dynamic cell seeding of plasma-treated scaffolds. (**B**) CellTiter-Glo quantification data from the same scaffold lots (i.e., 6 days of incubation) showed similar results compared to the MTT quantification data. (**C**) Western blotting results revealed a dose-dependent and enhanced human collagen I synthesis and deposition within the scaffolds by the FE002 primary progenitor tenocytes compared to the patient primary tenocytes. Whole gel imaging for collagen I and actin is presented in Figure S8. OD, optical density.

Successful tendinous or ligament tissue replacement using a synthetic scaffold requires a form of progressive in vivo graft colonization by the tissues and cells of the patient. While the cytocompatibility and the cellular adhesion potentials of the considered FE002 primary progenitor tenocytes were already assessed, the cytocompatibility of the scaffold with patient primary tenocytes required characterization work (Figure 1 and Figure S2). Specifically, preliminary assays showed that patient primary tenocytes were capable of excellent cellular adhesion on major tendon ECM components (i.e., biological surfaces, e.g., collagen I, fibronectin) in vitro (Figure S2). Then, the MTT-based cytocompatibility assay, previously described for the FE002 primary progenitor cell source, was performed again on cell-seeded constructs bearing patient primary tenocytes. Qualitative and quantitative results of these assays confirmed similar behaviors between the FE002 primary progenitor cells and the patient cells on the Infinity-Lock 3 scaffold, with enhanced metabolic activity recorded in the patient tenocyte groups (Figure 2(A1,A2)).

Such similar behaviors between the two considered primary cell types were further confirmed in Live-Dead assays, in immunofluorescence assays (e.g., phalloidin, aggrecan revelation), and in MTT-based histology assays on the patient tenocyte-seeded samples (Figures S4, S6 and S7). Furthermore, MTT-based timepoint analyses performed on constructs bearing patient tenocytes demonstrated the cellular proliferation and the scaffold colonization potentials of the latter (Figure S7). Then, a Western blot analysis confirmed that total protein quantities on the scaffolds and specific protein (i.e., aggrecan, decorin, collagen I, actin) quantities on the scaffolds were significantly increased at the 3-week timepoint compared to the 1-week timepoint (full data not shown). Importantly, the gathered experimental data enabled to confirm in vitro that the artificial scaffolds behaved as intended in the presence of patient primary tenocytes (i.e., isolated from hand tendon tissue), namely with the physical provision/presence of an appropriate 3D environment allowing (i.e., passively) biological material ingrowth.

In view of optimizing the quantitative in-process controls for scaffold cellular colonization assessment, a CellTiter-Glo readout was used to comparatively characterize constructs bearing FE002 primary progenitor tenocytes or patient primary tenocytes (Figure 2B). The results were in line with the data obtained using the MTT-based readouts (i.e., presence of a dose-response relationship and slightly superior absolute values for the patient primary tenocyte groups, Figure 2(A1,A2),B). While the CellTiter-Glo readout is more sensitive and can be performed rapidly to obtain quantitative results, as compared to the MTT-based readout, the qualitative assessment of scaffold colonization (i.e., cellular adhesion and distribution) is however not possible. It should be noted that as both quantitative readouts are based on metabolic reactions, their use for the quantitative comparison of different cell types is not directly possible without prior normalization. In addition to the comparative and quantitative assessments of scaffold colonization potentials at a cellular level (i.e., MTT-based and CellTiter-Glo readouts), immunology-based readouts revealed that the constructs bearing FE002 primary progenitor tenocytes contained more synthesized and deposited ECM components (e.g., collagen I) compared to the patient primary tenocyte groups (Figure 2C). Close consideration of these potency-related results indicated that multiparametric controls (i.e., at least at cellular and proteomic levels) were necessary for appropriate endpoint construct or finished combination product assessment.

**Figure 3:**
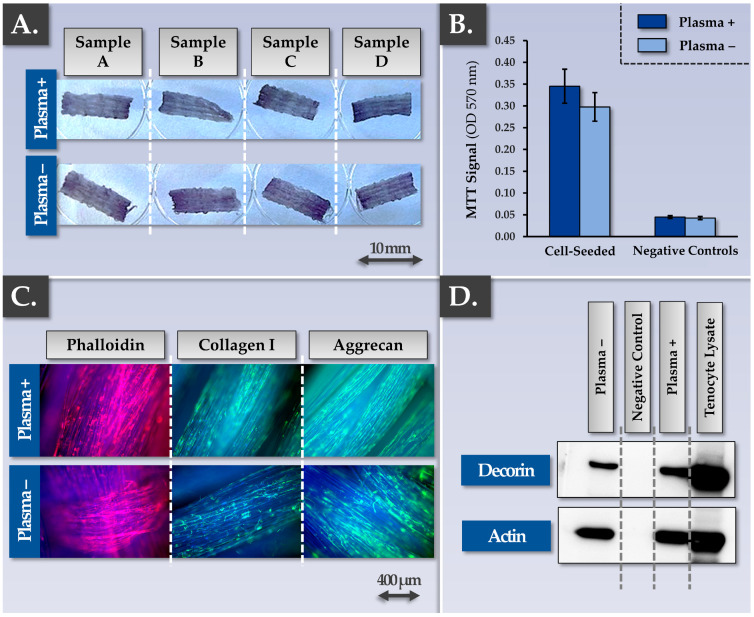
Results of the parallel qualification studies for plasma-treated and non-plasma-treated Infinity-Lock 3 scaffolds following FE002 primary progenitor tenocyte seeding and incubation for three weeks. (**A**,**B**) Results revealed no observable or statistically significant difference (i.e., *p*-value = 0.11) in the scaffold colonization potential of dynamically-seeded FE002 primary progenitor tenocytes between the two groups, as assessed by MTT staining and subsequent dye quantification. (**C**) Similar behaviors (i.e., phalloidin, collagen I, aggrecan synthesis and deposition) were evidenced for the two scaffold groups by immunofluorescence. (**D**) Similar behaviors in terms of decorin synthesis and deposition within the scaffolds were evidenced for the two considered groups by Western blotting. Whole gel imaging for decorin and actin is presented in Figure S9. OD, optical density.

As regards the type of Infinity-Lock 3 scaffolds to potentially be used in musculoskeletal tissue engineering, two technical processing options exist. The first option corresponds to the CE-marked scaffold (i.e., non-plasma treated scaffold). The second option corresponds to a plasma-treated scaffold, developed for enhanced wettability and cellular/tissular colonization properties [70]. Comparative multiparametric assessment (i.e., MTT- and immunology-based assays) of plasma-treated vs. non-plasma-treated scaffolds firstly revealed no significant differences between the groups, as all of the considered samples performed well in terms of functionality parameters (Figure 3).

Specifically, FE002 primary progenitor tenocyte adhesion on the scaffolds was not found to be influenced by scaffold plasma pre-treatment (Figure 3A,B). The whole construct surface was indeed covered with cells and ECM proteins such as actin, collagen I, aggrecan, and decorin (Figure 3C). The observed equivalence between the plasma-treated and the non-plasma-treated Infinity-Lock 3 scaffolds was further investigated in endpoint using comparative grading of efficacy/potency-related parameters of the cellular component of the combined finished product (Table 2).

Therein, the results confirmed that all of the considered samples were characterized by good performance for all of the investigated parameters (Table 2). At this point of the study, the available knowledge and data about cellular colonization of the Infinity-Lock 3 scaffold and ECM component synthesis/deposition throughout the scaffold enabled the establishment of a theoretical model describing the various steps and functionality-related mechanisms at play during construct cell-seeding and incubation (Figure S10). At this point, the protocol for obtaining the Infinity-Lock 3 constructs bearing allogeneic FE002 primary progenitor tenocytes was assessed as being established and validated, with multiparametric characterization of the obtained bio-enhanced grafts (Figure 1 and Figure 3, Table 2).

### 3.3. Bio-Enhanced Constructs Bearing Viable FE002 Primary Progenitor Tenocytes May Potentially Be Converted into Temperature-Stable & Devitalized Cell-Based Therapeutic Products

A proof-of-concept sub-study was then carried out in order to investigate the potential for further processing (i.e., stabilization by lyophilization, γ-irradiation terminal sterilization) of the obtained FE002 progenitor cell-bearing constructs. The result indicated that construct lyophilization resulted in the obtention of temperature-stabilized grafts, within a devitalized cellular therapeutic product setting (Figures S11 and S12, Table 3).

Furthermore, while lyophilized construct terminal sterilization was not technically excluded at this point (i.e., significant residual presence of cellular materials and ECM components within the irradiated samples), it was assessed that extensive further formulation work and processing optimization was required, in order to potentially obtain appropriate devitalized cellular or cell-free constructs (Figures S11 and S12, Table 3).

### 3.4. Bio-Enhanced Constructs Bearing FE002 Primary Progenitor Tenocytes May Be Obtained Using an Optimized and GMP-Transposable Manufacturing Process

The specific bases for allogeneic musculoskeletal tissue engineering using FE002 primary progenitor tenocytes and Infinity-Lock 3 scaffolds were set forth in the previous sections. In order to further establish an optimized and GMP-transposable process for cell-seeded construct manufacture, further in vitro studies were performed. Specifically, the first technical aspect of optimization to be investigated pertained to the state of the FE002 progenitor cell seeding materials (i.e., cellular active substance form) at the start of the finished combination product manufacturing phase. Specifically, all of the in vitro assays presented in the previous sections of the study were performed with fresh cellular active substance materials. Therefore, the first objective of the optimization phase was to use cryopreserved cellular active substance materials instead (i.e., for scaffold seeding), with extemporaneous thawing of the cell seeding lot (i.e., for temporal decoupling of the 2D cell expansion phase and the 3D construct manufacturing process). The second objective of the optimization phase was to establish a temporally condensed construct incubation process, for overall manufacturing resource and therapeutic pathway rationalization. Therefore, parametrically controlled cellular active substance and scaffold-based cell-bearing construct manufacturing processes were established, based on existing practices in musculoskeletal cell-based therapeutic approaches (Figures S13 and S14).

**Figure 4:**
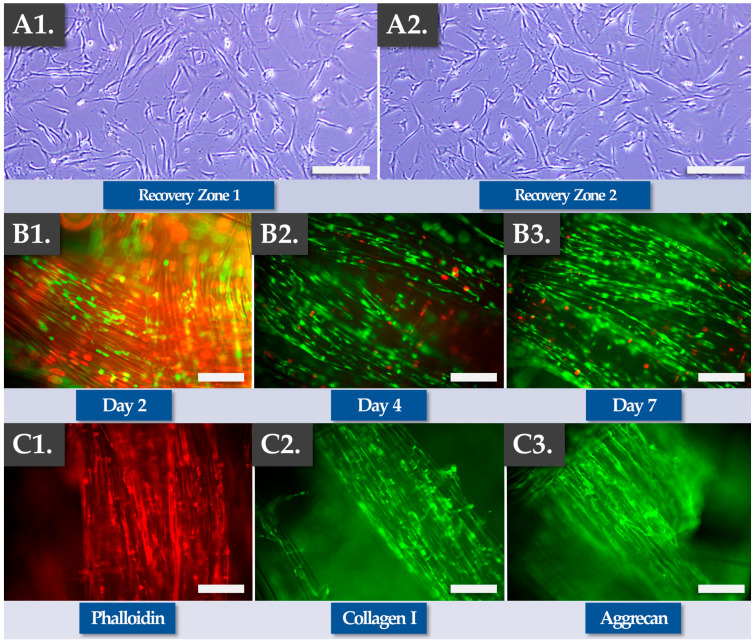
Results of finished product manufacturing process optimization work, using FE002 primary progenitor tenocytes and Infinity-Lock 3 scaffolds in culture for 7 days. (**A1**,**A2**) Cell recovery assays confirmed that the seeded cells conserved in vitro adhesion and proliferation capacities following direct initiation from cryostorage. Scale bars = 75 µm. (**B1**–**B3**) Iterative Live-Dead assays showed that the cells adhered throughout the scaffold, aligned themselves along the fibers, and spread along the fibers during proliferation. Several dead cells (i.e., in red fluorescence) could be observed, yet most of the cells were found to be viable (i.e., in green fluorescence). Overall, the proportion of viable cells was found to be more important at the 4-day and the 7-day timepoints. (**C1**–**C3**) Endpoint immunostainings were found to be positive for phalloidin, collagen 1, and aggrecan, confirming cellular alignment and extracellular matrix synthesis and deposition along the scaffold fibers after 7 days of incubation.

Experimentally, cryopreserved FE002 primary progenitor tenocyte cellular active substance lots were directly prepared and used to dynamically seed Infinity-Lock 3 scaffolds (i.e., high seeding cell dose of 10^5^ cells/cm of scaffold) and the constructs were incubated as described previously for a time-period of 7 days. Cell recovery controls were performed in 2D culture to assess the cellular adhesion and proliferation potentials/behaviors after thawing. The results of the optimization studies firstly confirmed the quality-related attributes (i.e., in vitro adhesion, proliferation) of the cellular active substance following extemporaneous thawing as equivalent to the same attributes of the fresh cellular active substance (Figure 4(A1,A2)).

Secondly, the results confirmed the functionality-related attributes of the FE002 primary progenitor tenocytes during and after construct incubation (i.e., cellular adhesion and proliferation along the scaffold fibers, Figure 4(B1–B3)). Specifically, Live-Dead data confirmed cellular viability maintenance and proliferation within the construct, especially at the two later timepoints (Figure 4(B1–B3)). Importantly, a minoritarian yet significant amount of non-viable cells (i.e., in red fluorescence) were recorded at the 2-day timepoint, while only small amounts of non-viable cells were recorded at later timepoints (Figure 4(B1–B3) and Figure S15). Such results confirmed the need for a minimal in vitro incubation period of the cell-seeded constructs of at least 4 days, in order to maximize in situ cellular viability and function. Specific endpoint Live-Dead assays showed adherent, viable, and highly organized FE002 primary progenitor tenocyte networks throughout the scaffolds (Figure S16). Thirdly, the results confirmed the functionality-related aspects of the FE002 primary progenitor tenocytes following construct incubation (e.g., ECM component synthesis and deposition along the scaffold fibers, Figure 4(C1–C3), Figures S17 and S18). Specifically, immunohistology performed at the 7-day timepoint showed that the scaffold fibers were coated with actin, phalloidin, collagen I, and aggrecan (Figure 4(C1–C3)). Overall, no significant differences were observed in terms of quality, purity, and potency-related parameters between the standard combined finished product manufacturing protocol (i.e., low cell seeding dose, 14 days of incubation) and the optimized protocol (i.e., high cell seeding dose, 7 days of incubation, Table 4).

Of note, both the plasma-treated and the non-plasma-treated Infinity-Lock 3 scaffolds were used within the optimized cell-seeded construct manufacturing protocol. Therein, no significant differences were evidenced between both scaffold types (Figures S15–S18). At this point of the study, the optimized protocol for obtaining clinically usable constructs bearing allogeneic FE002 primary progenitor tenocytes was assessed as being established and validated, in conformity with the corresponding controlled and parametrically defined manufacturing processes (Figures S13 and S14, Tables S2–S5). Overall, it was shown that the combined finished products, displaying appropriate critical and key quality attributes, could be rapidly manufactured using a simple GMP-transposable process, starting with cryopreserved FE002 cellular active substance materials.

### 3.5. Infinity-Lock 3 Constructs May Be Applied in Several Indications of Cell-Assisted Hand Ligament Regenerative Medicine

**Figure 5:**
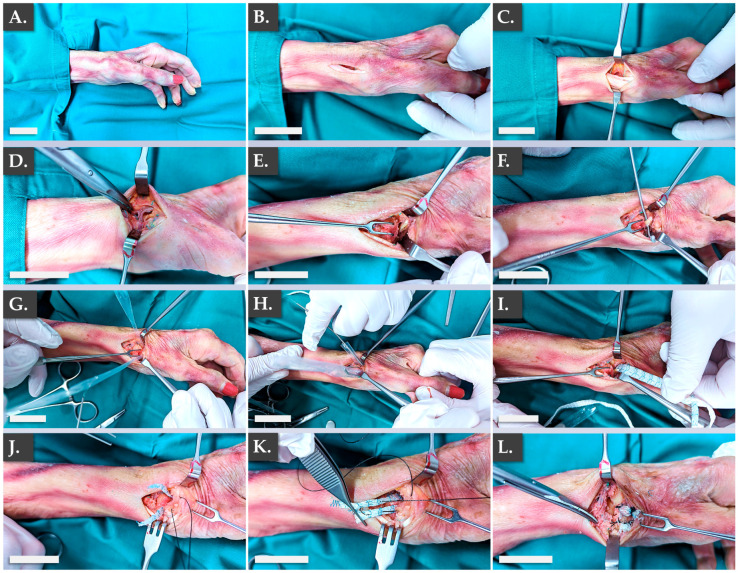
Illustrated step-by-step surgical overview of the ligamento-suspension plasty after trapeziectomy procedure using the Infinity-Lock 3 construct. (**A**) Initial setup. (**B**) Incision. (**C**) Exposure of APL and EPB tendons. (**D**) Exposure of the dorsal branch of the radial nerve. (**E**) FCR exposure. (**F**) FlexPasser installation. (**G**) Sheath installation. (**H**,**I**) Installation of the Infinity-Lock 3 construct using the sheath. (**J**) Removal of excess synthetic graft material. (**K**,**L**) Suturing of the Infinity-Lock 3 construct to the bone. Scale bars = 2.5 cm. APL, abductor pollicis longus; EPB, extensor pollicis brevis; FCR, flexor carpi radialis.

The intended therapeutic uses of the FE002 primary progenitor tenocyte-seeded constructs comprise several applications in the surgical management of hand tendon/ligament reconstructive interventions. In order to specifically verify the applicability of the Infinity-Lock 3 scaffold in two of the considered therapeutic indications, an ex vivo anatomical model was used. The first part of the ex vivo study enabled to confirm the applicability of the Infinity-Lock 3 construct for ligamento-suspension plasty after trapeziectomy (Figure 5).

**Figure 6:**
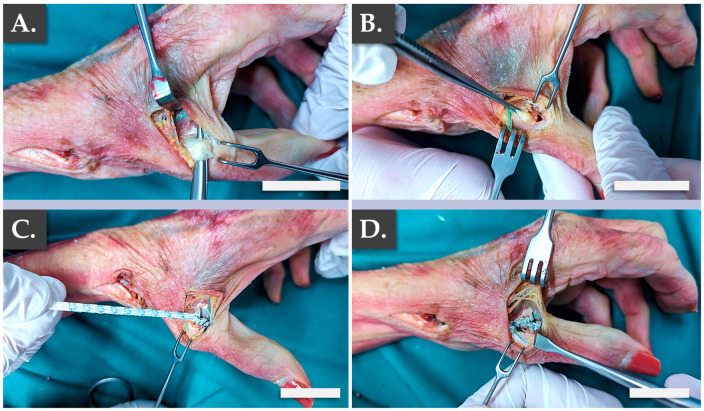
Illustrated step-by-step surgical overview of the thumb metacarpo-phalangeal ulnar collateral ligamentoplasty. (**A**) Exposure of the thumb UCL. (**B**) Ablation of a portion of the thumb UCL. (**C**) Distal suture of the Infinity-Lock 3 construct. (**D**) Proximal suture of the Infinity-Lock 3 construct. Scale bars = 2.5 cm. UCL, ulnar collateral ligament.

Specifically, it was confirmed that the structural and physical roles of such grafts were satisfactorily filled by the Infinity-Lock 3 construct, based on the surgical assessments of the authors. This application was considered of particular interest for patients presenting severe local osteoarthritis symptoms. Notably, it was noted that the length of the Infinity-Lock 3 construct was substantially reduced, following suturing and resection of excess synthetic graft materials, confirming the need for the production of excess graft material lengths (i.e., to comply with handling and surgical implantation needs, Figure 5J–L). The second part of the ex vivo study enabled to confirm the applicability of the Infinity-Lock 3 construct for thumb metacarpo-phalangeal ulnar collateral ligamentoplasty (Figure 6).

It was confirmed once more that the structural and physical roles of such grafts were satisfactorily filled by the Infinity-Lock 3 construct, based on the surgical assessments of the authors (Video A, Video B). This application was considered of particular interest for patients presenting acute UCL ruptures, also known as “skiers’ thumb”. Specifically, it was confirmed from a surgical point-of-view that the construct was adapted for the considered use and that the intervention resulted in the obtention or restoration of appropriate thumb mobility (Video A, Video B). Overall, the presented ex vivo results enabled to establish surgical proofs-of-concept and surgical protocols for hand ligament regenerative medicine, in view of the further translational and clinical studies to be performed with the considered allogeneic bioengineered grafts.

## 4. Discussion

### 4.1. Quality and Efficacy Parameters/Attributes: Cytocompatibility, Cellular Function, and Proteomic Constituents in the FE002 Primary Progenitor Tenocyte-Bearing Constructs

The general approach to musculoskeletal tissue engineering adopted within this study was firstly oriented toward finished product quality attribute maximization and secondly toward technical simplification, for the eventual obtention of overall manufacturing efficiency (Figures S1A and S1B). As is required by legal and normative documentation relative to novel cell-based or cell-containing products, the study focused on two main successive phases, namely the FE002 cellular active substance production and then the scaffold-based cell-bearing combined finished product production. As concerns the former, an extensive body of knowledge around the FE002 primary progenitor tenocyte clinical grade cell source predated the present study and was largely reported in the literature [19,39,55,56,57,58,71]. Specifically, multiple aspects of FE002 primary progenitor cell type characterization and qualification have been investigated, up to and including an in vivo GLP study in rabbits [39,71]. Furthermore, extensive validation work pertaining to primary progenitor cell type manufacturing in multi-tiered biobanking systems enabled to confirm the stability and the sustainability of the therapeutic cell source, based on in-house experience with GMP-produced and clinically applied alternative FE002 primary progenitor cells (e.g., FE002 dermal progenitor fibroblasts) [39,72].

Generally, an important concern regarding the in vivo implantation of viable exogeneous cells for therapeutic purposes is the risk of tumor formation. Notwithstanding the available data (i.e., in vitro, in ovo, and in vivo) relative to FE002 primary progenitor tenocyte safety (i.e., iterative karyotyping, soft agar tumorigenicity assay, CAM model, rabbit model), additional documentation of cellular active substance safety attributes was required [39,71]. Therefore, cell type lifespan characterization assays (e.g., in vitro senescence confirmation by evolutive population doubling value assessment and β-galactosidase activity determination) enabled to unequivocally confirm the primary nature of diploid FE002 primary progenitor tenocytes (Figure S3). Secondly, the results of the determination of telomerase activity within the FE002 primary progenitor cellular active substance fell in line with the data gathered the in soft agar and in vivo assays, namely the inability of the FE002 primary progenitor tenocytes to develop tumoral growth/behaviors in the retained experimental settings (Table S1) [39]. Specifically, the telomerase activity quantification assays enabled to provide a quantitative assessment for an important parameter related to safety attributes of the cellular active substance, contrasting with the soft agar and in vivo assays (i.e., descriptive or semi-quantitative, Table S1) [39].

Of note, telomeres protect chromosome ends against chromosomal fusion, recombination, or terminal DNA degradation [73]. Telomeres progressively shorten during processes of DNA replication and cell division in primary cell types (i.e., non-continuous cells), eventually leading to a progressive stop in replication and therefore resulting in cell senescence [73]. Telomerase is a reverse transcriptase enzyme capable of adding telomeric repeats to chromosome ends. In somatic human cells, telomerase activity is decreased after birth resulting in telomere length shortening with each cell division [73]. In contrast, cancer cells have high telomerase activity resulting in telomere maintenance and cell immortality [73]. The original data gathered on FE002 primary progenitor tenocytes, when compared to that of known cancerous cell lines (i.e., HeLa and HCT-116 positive controls), confirmed the appropriate level of function of telomerase in the cellular active substance of interest (Table S1).

Overall, the aggregated safety-related data around the FE002 primary progenitor tenocyte source was assessed as appropriate and conforming to the further considered therapeutic use of such cells in human regenerative medicine [39]. Specifically, the telomerase assay results were interpreted positively in light of the fact that at least one continuous cell line (i.e., immortalized cells) has been safely and effectively used in large-scale orthopedic clinical trials, with no reported safety concerns linked to inherent cellular active substance safety parameters (i.e., Invossa, Kolon TissueGene, Rockville, MD, USA) [74]. Therefore, while classical approaches to safety evaluation of novel cell-based therapies remain of critical importance before initiating clinical investigational use, recent developments and the available clinical data should also be factored in the corresponding risk analyses.

With regards to functionality-oriented aspects of FE002 primary progenitor cell type characterization, the original in vitro data presented in this study (e.g., cellular adhesion on ECM-coated surfaces) confirmed the biocompatibility data previously gathered using ex vivo decellularized equine tendon tissues (Figure S2) [19]. Furthermore, detailed investigation into the interactions between the FE002 primary progenitor tenocytes and the synthetic Infinity-Lock 3 scaffold have confirmed the compatibility and the function at cellular and proteinic levels (Figure 1 and Figure 4). From a mechanistic point-of-view, the rationale for combining therapeutic FE002 primary progenitor tenocytes on synthetic scaffolds may be considered as multifaceted. Firstly, within the context of hand ligament reconstructive surgery, the scaffold itself passively exerts the principal (i.e., necessary and sufficient) mode of action (i.e., structural tissular repair/replacement). Thus, the mode of action of the biological constituents (i.e., allogeneic FE002 cells and materials produced by them) is ancillary to that of the scaffold, when considering the finished combination product (Figure S10). Secondly, it is known that patient tissues and cells colonize the passive scaffold, in a manner or rate dependent upon the structural specificities of the synthetic device, based on existing in vivo preclinical and clinical reports (Tables S6–S8) [60,68]. Therefore, the use of a biologically-enhanced construct bearing allogeneic FE002 progenitor materials has the potential to qualitatively enhance graft bio-integration following implantation (e.g., scaffold colonization by host cells, avoidance of tissue adhesions, regulation of inflammation, modulation of implantation tissular environment) [75,76].

Specifically, the FE002 progenitor cell-bearing Infinity-Lock 3 constructs are known to be covered in biological materials (i.e., allogeneic progenitor cells, ECM constituents), which can potentially additionally or actively (i.e., in contrast to the passive nature of the barren scaffold itself in that regard) enable colonization of the graft by endogenous host tissues and cells. It was shown that scaffold colonization with FE002 primary progenitor tenocytes resulted in fiber coating with collagen I, decorin, and aggrecan, which are known tendon/ligament ECM proteins. Therefore, ECM deposition should create a different kind of localized favorable environment for integration by the surrounding patient tissues, through biological enhancement (e.g., biological priming) of the scaffold. Indeed, one major identified problem of tendinous tissue healing is the reduced quality of repair, leading to important rates of tissue re-tear [5,6,7,15]. Modern strategies to improve tendon healing outcomes are oriented toward biological-enhanced (e.g., growth factors, PRP, or various therapeutic cell sources) devices, with encouraging results (Table S9) [3,12]. Therein, the effects of bio-supplementation in tendon and ligament surgery have notably been investigated for several decades [75,76]. It was specifically shown that the combination of artificial LK scaffolds with a strip of fascia lata or infrapatellar fat pad accelerated tissue induction and increased the remodeling processes in vivo [75,76]. Such elements strongly support the considered use of FE002 primary progenitor materials for Infinity-Lock 3 scaffold bio-enhancement from a mechanistic viewpoint, aiming to provide biological cues for holistically optimized tissular repair/regeneration.

At the proteomic level, the experimental data has shown that the considered FE002 progenitor cellular active substance was composed of collagens, ECM glycoproteins, and ECM proteoglycans (Table 1) [55]. Data analyses revealed that the reported protein panel for the FE002 primary progenitor cells was highly similar to that of the patient primary tenocytes considered in this study and additional cross-referencing was made to reported proteomic analyses of human tendons (i.e., relative comparison possible) [69]. Importantly, this analysis demonstrated that FE002 primary progenitor tenocytes maintain the expression of critical human tendon ECM proteins after in vitro monolayer expansion and cell banking (Table 1). While collagen represents 60–85% of tendon dry mass and collagen I is the most abundant form of collagen in tendon tissue, additional minor ECM proteins have been identified in the FE002 progenitor cellular active substance and are important for tendon function and tendon health (Table 1) [77]. In the collagen family, the identified COL1, COL3, but also COL5, COL6, and COL12 proteins are known for their role in fibrillogenesis, and FE002 primary progenitor tenocytes also express a panel of ECM-associated glycoproteins (e.g., COMP, fibronectin, tenascin-C, Table 1). Furthermore, large proteoglycans (e.g., aggrecan) and SLRPs proteoglycans (e.g., decorin, biglycan) were identified and are involved in several aspects of tendon biology (i.e., fibrillogenesis, modulation of cell proliferation, migration, or differentiation) (Table 1) [69,77]. Overall, it was confirmed that the considered FE002 primary progenitor tenocytes contained proteinic constituents that are characteristic of human tendinous tissues and that could be of functional interest within the presented scaffold-based progenitor cell-bearing combination product, for bio-enhancement by synthetic fiber coating (Table 1) [69]. Concomitantly to ECM protein deposition throughout the Infinity-Lock 3 scaffolds, the FE002 primary progenitor tenocytes are also a source of additional biological factors (e.g., FGF-2 or HGF growth factors), which can locally assist and potentially promote the complex processes of tissue healing, in a similar way as PRP or MSC injections/grafting [78,79,80,81,82,83,84,85,86,87,88]. Therein, the use of standardized therapeutic primary cell sources (e.g., clinical grade FE002 primary progenitor cell sources) may be considered as advantageous over the use of genetically manipulated materials, from a technical simplicity and an in vivo safety standpoint [89,90,91,92,93,94,95]. Importantly, while new tendon/ligament tissue is forming and migrating within the implanted construct, the scaffold itself possesses, by design, the physical properties allowing to withstand the mechanical constraints applied onto the articulation [60,76]. From a technical and processing point-of-view, the present study set forth several proofs-of-concept for combination product preparation, stabilization, and sterilization. While finished product terminal irradiating sterilization was not technically excluded based on the available functional readouts, it was assessed that significant further studies were required in order to obtain acceptable off-the-shelf combination constructs. It is important to note that process parameters and product attributes are specific to the applied process and that the requirements for the final form of the product notably depend on the intended clinical use.

Generally, the reasoning and rationale for cell-seeded construct use in musculoskeletal regenerative medicine is based on existing experience with chondrocyte therapy, wherein biological enhancement of matrices has been studied [96]. This approach aims to prime the implanted grafts and to provide stimulatory signals to the surrounding environment in order to optimize tissular repair [96]. Extensive characterization work and clinical hindsight are available in this domain, notably for autologous chondrocytes cultured on synthetic scaffolds (e.g., Chondro-Gide, Optimaix) in vitro [96]. By analogy, the obtained functionality-related data gathered on the FE002 primary progenitor tenocyte cellular active substance of interest confirmed that despite “substantial manipulation” of the cells in serial monolayer culture, cellular quality attributes and functionality are conserved. Such considerations are based on widely known and regulatorily accepted manufacturing and control parameters in the field of cartilage tissue engineering (i.e., comprising a cell culture expansion phase) [96]. Therein, demonstration of the ability of the therapeutic cells to readopt a chondrogenic behavior in 3D culture (i.e., following the 2D expansion phase, characterized by a transient loss of chondrogenicity) constitutes the basis of in vitro product potency characterization and qualification. By extension, for the FE002 cell-seeded Infinity-Lock 3 constructs, the functional biological aspects were documented at the cellular and proteomic levels, which are more informative/relevant (i.e., functionally impactful) than gene expression profiles, for example. In particular, such functional attributes of the cellular active substance (i.e., 3D cellular attachment and proliferation, ECM scaffold fiber coating) may be used as in vitro potency assays for alternative cytotherapeutic formulations (e.g., injectable FE002 cell suspensions in HA-based hydrogels or in autologous human serum-supplemented saline solutions) containing viable FE002 primary progenitor tenocytes [56].

Overall, both the FE002 progenitor cellular active substance and the combined finished product were shown to possess attributes and functions which are in line with the intended use of the bio-enhanced constructs, in view of optimizing musculoskeletal tissue reconstruction processes (Figure 4, Table 1). Specifically, quality- and functionality-related parameters/attributes of the FE002 primary progenitor tenocytes have been analyzed and investigated in light of pre-existing work in cartilage tissue engineering. This angle is especially interesting, as the scientific, clinical, and regulatory dimensions of chondrocyte-based therapy are much more advanced and accepted than those in the scarce current field of clinical tenocyte-based regenerative therapies, providing a tangible comparison point within the musculoskeletal system.

### 4.2. Applicability of FE002 Primary Progenitor Tenocyte-Bearing Constructs in Allogeneic Cell-Assisted Hand Ligament Regenerative Medicine

For the sound development of novel cell-based or cell-containing combination products, several regulatory and clinical risks may be mitigated by the use of a material or device which has been previously approved and clinically used successfully (i.e., documented track-record). While several technologies and materials are currently commercialized for tendon and ligament reconstruction, the Neoligaments devices were selected based on the extensive available clinical hindsight and the high versatility in available device designs, enabling eventual widening of the clinical indications for alternative FE002 progenitor cell-seeded constructs (e.g., progenitor cell-assisted rotator cuff repair, Tables S6–S8) [20,24,25,26,59,60,61,62,63,64,65,66,67,68].

In detail, the studied Infinity-Lock 3 system is a CE-marked medical device in the form of an open weave tape with densely woven sections (https://www.neoligaments.com/, accessed on 8 June 2023, Figure 5). This device is a sterile and single-use, non-absorbable, implantable tape made from polyester (i.e., 100% polyethylene terephthalate). Listed clinical indications comprise soft tissue approximation and structural reconstruction in musculoskeletal surgical procedures, such as the reconstruction of damaged or torn ligaments and tendons. The Infinity-Lock system is an adaptation of the Leeds-Keio ligament (LK ligament), which has been tested and validated in porcine and in canine in vivo models (Table S6) [75,76]. The LK synthetic scaffolds were developed several decades ago and were clinically used for ligament and tendon repair surgeries, wherein long-term patient follow-up data are available (Tables S7 and S8) [62,63,64,66,67].

Importantly, notable studies on the biocompatibility of such devices with human hand tendon tissues (e.g., study of tendon tissue ingrowth) have been considered to constitute the technical foundations for the design of the presented FE002 primary progenitor tenocyte-bearing bio-enhanced graft [60,68]. Therein, healthy human adult extensor tendon tissue was sutured on both ends of 0.5-cm synthetic scaffold segments and then kept in culture for several weeks [60]. Cell migration and proliferation from the tendon tissue onto the scaffold were observed [60]. Overall, the original experimental in vitro data gathered in the present study have demonstrated that FE002 primary progenitor cells and patient primary tenocytes could adhere to the Infinity-Lock 3 scaffold and that the local 3D environment was also suitable for cell proliferation, migration, and ECM protein deposition (Figure 4). Combined with the historical preclinical and clinical data available on the scaffold material itself, a clear demonstration was laid down herein for the applicability of the bio-enhanced graft in the intended clinical uses (Tables S7 and S8).

Of note, retrospective analyses and prospective investigations have highlighted several risks associated with the use of the considered synthetic tapes, such as minimal acute inflammatory tissue reaction, transitory local irritation, allergic reaction and discomfort, or skin breakdown due to prominent knots or fixation devices under the skin (Tables S7 and S8). Such elements were interpreted as being coherent with the process of implanting a biocompatible material in the human body, where the considered material passively and progressively undergoes encasement and colonization by the surrounding native tissues and cellular components [4,6,9]. While the structural and topographical specificities of the implanted device may play a role in the rate of colonization, it is known that suboptimal integration may be characterized by tissue adhesions (i.e., incurring additional morbidity) [14,15,16,17,22]. This bottleneck may be averted with the use of a silicone sheath during surgical hand tendon or ligament reconstruction, to limit the occurrence of tissular adhesions. Furthermore, based on the known behaviors of the polyester scaffold itself in vivo, the considered use of biologically-enhanced constructs bears the potential of optimizing colonization by native tissues and bio-integration, through “priming” of the synthetic surfaces with FE002 primary progenitor tenocyte materials [60,75,76]. The objective of such an approach consists in potentially diminishing the rate of tissular adhesions and the need for secondary/corrective surgeries in the clinical setting. Overall, the existing body of knowledge on the Infinity-Lock 3 scaffold, on the FE002 primary progenitor tenocytes, and on the combination thereof enables to confirm and set forward the applicability of the considered progenitor cell-bearing constructs in allogeneic cell-assisted hand ligament regenerative medicine.

### 4.3. Current Translational Development of Cell Therapies for Tendon & Ligament Repair/Regeneration: Alternative Tenocyte-Based Regenerative Medicine Protocols

While novel CAR-T cell therapies attract much of the attention in the current field of oncology, somatic cell therapy is notably already effectively used at large scales in orthopedics for the treatment of large cartilage defects. First-in-human autologous chondrocyte implantations (ACI) date back to 1994 with the original Brittberg studies and the technique has incrementally evolved over time to optimize the cell delivery methods to the chondral/osteochondral defect site [96]. Long-term studies are now available for ACI and demonstrate the positive clinical outcomes of such regenerative strategies, encouraging the development of similar technologies for the treatment of other musculoskeletal pathologies and affections [97]. Namely, multiple therapeutic cell-based approaches (i.e., autologous and allogeneic, cell-based and cell-free) have been investigated in vivo and at clinical levels for tendon tissue regenerative medicine (Table S9) [2,3,12,13,98,99].

For optimal contextualization of the allogeneic bioengineering solution investigated herein (i.e., cytotherapeutic application of FE002 primary progenitor tenocytes) and comparison to similar protocols in use at the clinical level, the autologous example of the Ortho-ATI (Orthocell, Murdoch, Australia) technology is presented and discussed. Orthocell Ltd., an Australian biotechnology company, transposed the accumulated knowledge around ACI to the development of an autologous tenocyte injection (ATI) strategy. Autologous tenocyte supplementation efficacy has firstly been demonstrated in rabbit models of acute and chronic tendinopathy, in which autologous tenocytes were delivered through grafting of a tenocyte-seeded biological scaffold (i.e., ACI Maix and Restore matrix) or through direct cell injection, respectively, at the site of injury [100,101]. After 8 weeks, a clear improvement in the healing process compared to the no cell treatment groups was observed. Specifically, inflammation and angiogenesis were reduced, collagen I expression was increased, and tendon fiber structure, arrangement, or ultimate load failure were improved [100,101]. The therapeutic tenocytes also accelerated the scaffold absorption by the host and part of the injected cells were still identified at the injured site at the end of the studies. These experimental observations strongly suggested that tenocyte delivery enhanced the intrinsically limited healing process of tendon tissues and could constitute a clinically potent treatment in effective tendinopathy management [100,101].

The first long-term clinical study results were reported for the use of Ortho-ATI in the treatment of chronic resistant lateral epicondylitis (LE) (i.e., clinical trial number ACTRN12607000402448) [43,52]. Therein, 16 patients assessed as refractory to classical non-surgical treatments were enrolled in the study. Patellar tendon biopsies were performed under local anesthesia and the autologous tenocytes (i.e., starting cellular materials) were isolated for expansion. The therapeutic cell lots (i.e., cellular active substance) were validated through the analysis of a panel of CD markers (i.e., CD18, CD34, CD44, CD45, CD90, CD106, CD46, and Stro-1) and the gene expression analysis of specific genes (i.e., ACAN, Col I, Col III, decorin, MAGP2, Mohawk, scleraxis, and TGF-β). As concerns the finished product, 4–10 × 10^6^ cells were formulated with autologous human serum (aHS) and injected under ultrasound guidance into the tendinopathic site [43,52]. At 4 weeks post-surgery, the patients could resume sport activities. Lasting symptom improvements were recorded after a mean follow-up time of 4.5 years. Therein, the VAS pain, QuickDASH, and grip strength scores improved by 78%, 84%, and 132.6% respectively and no ossification was observed at the elbow injection site [43,52].

The efficacy of Ortho-ATI does not seem limited to LE, as sustained improvements of symptoms have also been recorded in rotator cuff repair (i.e., clinical trial number ACTRN12617000684325) and for gluteal tendinopathy [102]. Based on the currently available data, the ATI therapeutic approach appears to provide a safe and effective way to provide quick and lasting symptom improvements in patients who suffered for several months, did not respond to classical treatments (e.g., PRP, corticosteroids), and eventually would have to go through more invasive surgeries [100,101,102]. Similarly to ACI, the exact mechanism of action of Ortho-ATI is unknown but most probably impacts multiple aspects of tendinopathy (i.e., cellular integration and ECM synthesis, growth factor supply, inflammation modulation), shifting the balance from a degenerative state to a regenerative state, which is not possible with conventional treatments [100,101,102].

Considering the promising Ortho-ATI safety and efficacy results, development of the next generation of tenocyte-based cell therapies using an allogeneic clinical grade cell source (e.g., FE002 progenitor cell sources) can tangibly be envisioned [39,100,101]. Allogeneic cell sources (e.g., stem and progenitor cells) have been previously proposed by several authors for musculoskeletal tissue engineering and for the optimal restoration promotion of tendons in particular [103,104,105,106]. Specific focus was set on the therapeutic contributions of ECM components, given their strong implications in specific tissular healing processes [107,108,109,110,111,112,113]. As concerns the specific use of the FE002 primary progenitor tenocyte source of interest for tendon and ligament regenerative medicine, several formulation approaches and bioengineering concepts have previously been reported (e.g., injectable cellular hydrogel suspension, cell seeding of decellularized equine tendons, use in bio-fabrication settings) [39,54,56,57,58]. Generally, FE002 primary progenitor tenocytes have been extensively studied and have demonstrated the potential of becoming a standardized cell source for tendon and ligament disorder treatment [39,71]. Specifically, FE002 primary progenitor tenocytes are stable and pre-terminally differentiated cells, the safety profile shows low risks (i.e., no anchorage-independent cell growth potential, limited lifespan in culture, low telomerase activity), and extensive cryopreserved lots of cellular active substance can be established and validated (Table S1) [39]. Thereafter, FE002 primary progenitor tenocytes were shown to be compatible with hyaluronic acid formulations, remained viable after extrusion through syringes, can be lyophilized, and can be seeded onto biological and synthetic scaffolds (Figure 4) [19,39,54,56,57,58]. Such approaches have confirmed the high versatility of FE002 primary progenitor tenocytes for novel allogeneic cell-based and cell-containing therapies in a number of clinical indications (i.e., with adapting of the synthetic scaffold nature/shape/size and the surgical protocol) [39,56]. This versatility opens a multitude of options for the development of new allogeneic cell-based therapeutic products tailored to specific musculoskeletal pathologies.

### 4.4. Study Limitations and Future Perspectives

Several limitations have been identified within this study. From a first technical viewpoint, the finished product conditioning and transport medium still needs to be specified and validated, while allowing for conservation of critical quality attributes for the entire finished product validity period. Based on parallel research, the product transport medium may be constituted by a hyaluronan-based hydrogel or an autologous serum-based saline solution with appropriate supplements [54,71]. From a second technical viewpoint, upscaling of the manufacturing protocol to whole Infinity-Lock 3 scaffolds shall be performed, in order to obtain clinically usable constructs of appropriate dimensions, based on the surgical needs and clinical demands (i.e., excess product length manufacture, Figure 5) [114,115,116,117,118]. While the scale of cell seeding and construct incubation may be different using whole Infinity-Lock 3 scaffolds, the extent of process validation studies performed using 1-cm scaffold subunits enables to robustly predict construct behavior in vitro. Specifically, appropriate methodological elements shall be used for the evaluation of the impact of changes in the cytotherapeutic product manufacturing process (e.g., ICH Q5E methodology). From a third technical viewpoint, further development and full validation of the control assays described in the present study shall be performed, specifically as concerns efficacy-related parameters. These aspects are however of prime importance only at later stages of clinical investigational use, according to applicable guidance documents (e.g., Potency Tests for Cellular and Gene Therapy Products) [119].

Future perspectives based on this study consist in the further translational qualification and investigational work around the FE002 primary progenitor tenocytes of cytotherapeutic interest (i.e., primarily in regenerative medicine applications for the hand, e.g., acute sharp force trauma or degenerative pathologies) [120]. Based on the fact that the FE002 primary progenitor tenocyte cellular active substance has been characterized and qualified using multiple safety-related assays and that in vivo cellular implantation has already been performed in a rabbit GLP study, the next steps of the planned translational work comprise a first-in-man clinical trial [39]. As previously mentioned, the present study sets forth important functionality-related parameters for the FE002 primary progenitor tenocytes (e.g., ECM component synthesis and deposition in 3D culture), contributing to the qualification of such primary allogeneic cells for alternative tissue engineering applications [121]. Specifically, diversification of the clinical indications for products containing such FE002 cells comprise the original protocols set forth by the authors for this cell source, namely the use of cellular hydrogel suspensions for intra-tendinous or peritendinous injection treatment of tendinopathies/tendinosis [39,56]. This approach, technically simplified as compared to the tissue engineering protocol using the synthetic Infinity-Lock 3 scaffold, may represent an optimized solution for overall cost management and for widespread clinical applicability, similarly to the well-known autologous ultrasound-guided use of PRP for tendinopathies in sports medicine [47,56].

## 5. Conclusions

The aim of the present study was to establish novel tissue engineering and surgical proofs-of-concept for a bio-enhanced artificial Neoligaments graft bearing cultured viable allogeneic FE002 primary progenitor tenocytes (i.e., clinical grade standardized cellular active substance). In vitro studies confirmed that the progenitor cell-seeded constructs could be obtained using optimized and GMP-transposable processes and were characterized by good quality and functionality-related parameters/attributes. The results of the study have notably shown that FE002 primary progenitor tenocytes were capable of cellular adhesion, proliferation, and homogeneous tendon ECM component synthesis/deposition throughout the Infinity-Lock 3 scaffold, with progressive and structurally-organized biological coating of the synthetic fibers. Ex vivo cadaveric work confirmed that the Infinity-Lock 3 constructs could be clinically applied in two indications of cell-assisted hand surgery (i.e., ligamento-suspension plasty after trapeziectomy and thumb metacarpo-phalangeal ulnar collateral ligamentoplasty). These original data were analyzed and discussed in light of the known behaviors of the synthetic Neoligaments scaffolds following in vivo implantation and of existing clinical practices using cultured autologous tenocytes for bioengineering and cell therapies (e.g., Ortho-ATI). Generally, specific discussion points about the available body of knowledge for the allogeneic FE002 progenitor cellular source and about the retained synthetic scaffold materials enabled the comprehensive assessment and general mitigation of the risks associated with the presented novel tissue engineering solution. Overall, this study enabled to set forth important proofs-of-concept for the translational development of an allogeneic tissue engineering protocol for hand ligament regenerative medicine, in view of further investigative clinical work.

## Figures and Tables

**Table 1:** Results of the comparative proteomic analysis for the determination of the major constituents in FE002 primary progenitor tenocyte and patient tenocyte samples (i.e., from cells expanded in vitro in monolayers). Relative quantitative data were expressed as fold change logarithms (i.e., base 2 log), with a significance threshold value specified at 0.9 for upregulation and an FDR threshold value specified at ≤0.01. The data are reported for three selected protein classes, namely collagens, ECM glycoproteins, and ECM proteoglycans. ECM, extracellular matrix; FC, fold change; FDR, false discovery rate.

Protein Class	Protein Name	Short Protein Name	Patient Tenocytes vs. FE002 Progenitor Tenocytes logFC Value	FDR Value
1. ECM Proteoglycans	Basement membrane-specific heparan sulfate proteoglycan core protein	HSPG2	−0.2981	≤0.01
	Versican core protein	VCAN	−0.4311	≤0.01
	Aggrecan core protein	ACAN	−0.8185	≤0.01
	Decorin	DCN	1.5648	≤0.01
	Biglycan	BGN	0.3762	≤0.01
	Prolargin	PRELP	0.3975	≤0.01
	Testican-1	SPOCK1	0.3765	≤0.01
	Podocan	PODN	1.1864	≤0.01
	Mimecan	OGN	−1.5874	≤0.01
2. Collagens	Collagen alpha-3(VI) chain	COL6A3	0.2684	≤0.01
	Collagen alpha-1(XII) chain	COL12A1	−0.4344	≤0.01
	Collagen alpha-2(I) chain	COL1A2	−0.9689	≤0.01
	Collagen alpha-1(I) chain	COL1A1	−1.2075	≤0.01
	Collagen alpha-1(XIV) chain	COL14A1	−2.0388	≤0.01
	Collagen alpha-2(VI) chain	COL6A2	0.1635	≤0.01
	Collagen alpha-1(III) chain	COL3A1	−0.8283	≤0.01
	Isoform 2 of Collagen alpha-1(V) chain	COL5A1	−1.3237	≤0.01
	Collagen alpha-1(XVIII) chain	COL18A1	−0.1641	≤0.01
	Collagen alpha-2(V) chain	COL5A2	−1.4452	≤0.01
	Collagen alpha-1(II) chain	COL2A1	0.6062	≤0.01
	Collagen alpha-1(XVI) chain	COL16A1	−0.3993	≤0.01
	Collagen alpha-1(IV) chain	COL4A1	−0.6277	≤0.01
	Collagen alpha-1(VIII) chain	COL8A1	0.7691	≤0.01
	Collagen alpha-1(XI) chain	COL11A1	−0.4923	≤0.01
3. ECM Glycoproteins	Fibronectin	FN1	0.9885	≤0.01
	Laminin subunit beta-2	LAMB2	−0.6965	≤0.01
	Tenascin	TNC	0.1536	≤0.01
	Laminin subunit alpha-4	LAMA4	−0.2982	≤0.01
	Laminin subunit gamma-1	LAMC1	−0.1818	≤0.01
	Peroxidasin homolog	PXDN	−0.1384	≤0.01
	von Willebrand factor A domain-containing protein 5A	VWA5A	0.6092	≤0.01
	Transforming growth factor-beta-induced protein ig-h3	TGFBI	0.3414	≤0.01
	Thrombospondin-1	THBS1	−0.4929	≤0.01
	Tenascin-X	TN-X	0.9108	≤0.01
	Laminin subunit beta-1	LAMB1	0.1839	≤0.01
	Lactadherin	MFGE8	−0.2366	≤0.01
	EGF-like repeat and discoidin I-like domain-containing protein 3	EDIL3	0.3395	≤0.01
	Procollagen C-endopeptidase enhancer 1	PCOLCE	−0.4078	≤0.01
	Laminin subunit alpha-3	LAMA3	−0.1923	≤0.01
	Extracellular matrix protein 1	ECM1	1.2584	≤0.01
	Fibulin-2	FBLN2	−0.5234	≤0.01
	Thrombospondin-2	THBS2	0.1871	≤0.01
	Latent-transforming growth factor beta-binding protein 1	LTBP1	−0.2777	≤0.01
	Collagen triple helix repeat-containing protein 1	CTHRC1	−2.3638	≤0.01
	Fibulin-1	FBLN1	0.5411	≤0.01
	Latent-transforming growth factor beta-binding protein 3	LTBP3	−0.3839	≤0.01
	Insulin-like growth factor-binding protein 7	IGFBP7	0.2386	≤0.01
	CCN family member 1	CYR61	0.8094	≤0.01
	Cysteine-rich with EGF-like domain protein 2	CRELD2	−0.6177	≤0.01
	Netrin-G1	NTNG1	−0.5626	≤0.01
	Latent-transforming growth factor beta-binding protein 2	LTBP2	0.9153	≤0.01
	Cartilage oligomeric matrix protein	COMP	−0.3154	≤0.01
	Cysteine-rich with EGF-like domain protein 1	CRELD1	−0.5640	≤0.01
	Insulin-like growth factor-binding protein 5	IGFBP5	−1.4395	≤0.01
	Adipocyte enhancer-binding protein 1	AEBP1	1.0318	≤0.01
	Insulin-like growth factor binding protein 3 isoform b	IGFBP3	0.9616	≤0.01
	Microfibril-associated glycoprotein 4	MFAP4	−2.3113	≤0.01
	Fibrillin-2	FBN2	−0.6129	≤0.01
	Slit homolog 3 protein	SLIT3	−0.3010	≤0.01
	Laminin subunit alpha-1	LAMA1	0.6983	≤0.01
	Slit homolog 2 protein	SLIT2	0.5349	≤0.01
	Thrombospondin-3	THBS3	−0.5152	≤0.01
	Target of Nesh-SH3	ABI3BP	0.5272	≤0.01
	Laminin subunit alpha-5	LAMA5	−0.7307	≤0.01
	Netrin-4	NTN4	−0.5776	≤0.01
	Matrix-remodeling-associated protein 5	MXRA5	−0.3548	≤0.01

**Table 2:** Grading table for the assessment of equivalence between plasma-treated and non-plasma-treated Infinity-Lock 3 scaffolds within the finished product manufacturing workflows. Equivalence was assessed for finished product efficacy-related parameters/attributes only (i.e., cellular components). ECM, extracellular matrix; 3D, three dimensions.

Efficacy Parameter Type	Controls/Assays	Targets & Acceptance Criteria	Endpoint Construct Gradings ^1^	
			Non-Plasma-Treated	Plasma-Treated
Cellular Viability Maintenance in 3D	MTT; Live-Dead	Presence of viable cells throughout the constructs	+++	+++
Cellular Quantity in 3D	MTT; Live-Dead	Presence of cells throughout the constructs; In amounts comparable to historical data	+++	+++
Cellular Adhesion & Cellular Morphology in 3D	Live-Dead	Presence of adherent cells throughout the constructs; Cellular alignment along the scaffold fibers	++	+++
Cellular Proliferation Capacity in 3D	CellTiter-Glo; Live-Dead	Presence of cells throughout the constructs in larger amounts than after cell seeding	+++	+++
Cellular Colonization Homogeneity in 3D	MTT; Live-Dead	Presence of homogeneously distributed cells throughout the constructs	+++	+++
Extracellular Matrix Synthesis & Deposition in 3D	Immunofluorescence; Immunohistochemistry	Presence of adherent ECM components along the fibers within the constructs	++	+++
Extracellular Matrix Deposition Homogeneity in 3D	Immunofluorescence	Presence of homogeneously distributed ECM throughout the constructs	+++	+++

^1^ Gradings were attributed as follows: (+++) = conforming, excellent performance; (++) = conforming, good performance; (+) = conforming; (±) = unclear, additional data required; (–) = non-conforming.

**Table 3:** Grading table for the assessment of lyophilized and lyophilized/irradiated constructs (i.e., cellular components) within exploratory finished product manufacturing optimization studies. ECM, extracellular matrix; 3D, three dimensions.

Efficacy Parameter Type	Controls/Assays	Targets & Acceptance Criteria	Endpoint Construct Gradings ^1^	
			Lyophilized	Lyophilized/Irradiated
Cellular Quantity in 3D	Live-Dead	Presence of cells throughout the constructs in amounts comparable to historical data	++	+
Cellular Adhesion & Cellular Morphology in 3D	Live-Dead	Presence of adherent cells throughout the constructs; Cellular alignment along the scaffold fibers	++	±
Cellular Colonization Homogeneity in 3D	MTT; Live-Dead	Presence of homogeneously distributed cells throughout the constructs	+++	±
Extracellular Matrix Synthesis & Deposition in 3D	Immunofluorescence	Presence of adherent ECM components along the fibers within the constructs	++	+
Extracellular Matrix Deposition Homogeneity in 3D	Immunofluorescence	Presence of homogeneously distributed ECM throughout the constructs	+++	±

^1^ Gradings were attributed as follows: (+++) = conforming, excellent performance; (++) = conforming, good performance; (+) = conforming; (±) = unclear, additional data required; (–) = non-conforming.

**Table 4:** Grading table for the assessment of equivalence between the standard protocol and the accelerated protocol within combined finished product manufacturing. Equivalence was assessed for finished product efficacy-related parameters/attributes only (i.e., cellular components) using the non-plasma-treated Infinity-Lock 3 scaffold. ECM, extracellular matrix; 3D, three dimensions.

Parameter Class	Parameter Type	Controls	Targets & Acceptance Criteria	Endpoint Construct Gradings ^1^	
				Standard Protocol 14 Days	Optimized Protocol 7 Days
Quality	Cellular Adherence/Viability in 2D	Recovery plates	Presence of viable & adherent cells in recovery plates	+++	+++
Quality	Cellular Quantity/Proliferation Capacity in 2D	Recovery plates	Presence of actively proliferating cells in recovery plates	+++	+++
Purity	Cellular Population Identity & Non-Contamination in 2D	Recovery plates	Specific cellular morphology comparable to historical data; Monomodal cellular population	+++	+++
Potency	Cellular Viability in 3D	MTT; Live-Dead	Presence of a majority of viable cells	+++	+++
Potency	Cellular Quantity/Proliferation Capacity in 3D	CellTiter-Glo; Live-Dead	Presence of actively proliferating cells on the constructs	+++	+++
Potency	Cellular Adhesion & Morphology in 3D	Live-Dead	Presence of cells throughout the constructs; Alignment of cells along the construct fibers	+++	+++
Potency	Cellular Colonization Homogeneity in 3D	MTT; Live-Dead	Homogeneous presence of cells throughout the constructs	+++	++
Potency	Extracellular Matrix Synthesis & Deposition	Immunohistochemistry; Immunofluorescence	Presence of adherent ECM components along the scaffold fibers	+++	+++
Potency	Extracellular Matrix Deposition Homogeneity in 3D	Immunofluorescence	Homogeneous ECM presence throughout the constructs	+++	+++

^1^ Gradings were attributed as follows: (+++) = conforming, excellent performance; (++) = conforming, good performance; (+) = conforming; (±) = unclear, additional data required; (–) = non-conforming.

## Data Availability

The data presented in this study are not publicly available due to legal and statutory restrictions. The data presented in this study are available upon written and reasonable request from the corresponding authors.

## Supplementary Materials

The following supporting information can be downloaded at: https://www.mdpi.com/article/10.3390/pharmaceutics15071873/s1, Figure S1A: General study design overview; Figure S1B: Illustrated study design phases; Figure S2: Results of in vitro cellular adhesion assays; Figure S3: Results of β-galactosidase staining assays; Figure S4: Results of Live-Dead assays for primary tenocytes on synthetic Infinity-Lock 3 scaffolds; Figure S5: Results of immunofluorescence assays for FE002 primary progenitor tenocytes on synthetic Infinity-Lock 3 scaffolds; Figure S6: Results of immunofluorescence assays for primary patient tenocytes on synthetic Infinity-Lock 3 scaffolds; Figure S7: Results of MTT-based cytocompatibility assays for primary patient tenocytes on synthetic Infinity-Lock 3 scaffolds; Figure S8: Whole gel imaging for collagen I Western blot analyses; Figure S9: Whole gel imaging for decorin Western blot analyses; Figure S10: Experimental model for the description of functionality-related attributes of FE002 primary progenitor tenocytes on synthetic Infinity-Lock 3 scaffolds; Figure S11: Results of Live-Dead assays for the assessment of the impact of cell-seeded construct processing; Figure S12: Results of immunofluorescence assays for the assessment of the impact of cell-seeded construct processing; Figure S13: Parametric and controlled manufacturing process established for the FE002 primary progenitor tenocyte cellular active substance; Figure S14: Parametric and controlled manufacturing process established for the cell-seeded constructs; Figure S15: Results of Live-Dead assays for the validation of the optimized construct manufacturing protocol; Figure S16: Results of Live-Dead assays for the validation of the optimized construct manufacturing protocol; Figure S17: Results of immunofluorescence assays for the validation of the optimized construct manufacturing protocol; Figure S18: Results immunofluorescence assays for the validation of the optimized construct manufacturing protocol; Table S1: Results of the telomerase activity quantification study; Table S2: Listing of CPP/KPP for cellular active substance manufacture; Table S3: Listing of CPP/KPP for finished product manufacture; Table S4: Listing of CQA/KQA for the cellular active substance; Table S5: Listing of CQA/KQA for the finished product; Table S6: Listing of available preclinical in vivo data on the synthetic constructs; Table S7: Listing of the available in vivo clinical data on the synthetic constructs; Table S8: Listing of post-market clinical follow-up studies performed on the synthetic constructs; Table S9: Summarized listing of human clinical trials using therapeutic cell-based products/therapies for tendon repair and regenerative medicine; Video A: Illustration for damaged UCL distal suture testing; Video B: Illustration for damaged UCL complete suture testing.

## Abbreviations

ACL	anterior cruciate ligament
ADRC	adipose-derived regenerative cells
APL	abductor pollicis longus
ASC	adipose-derived stem cells
BCA	bicinchoninic acid
BM-MSC	bone marrow-derived mesenchymal stem cells
BSA	bovine serum albumin
CAM	chorioallantoic membrane model
cATMP	combined advanced therapy medicinal product
CE	European conformity certification
CHUV	centre hospitalier universitaire vaudois
CPP	critical process parameter
CQA	critical quality attribute
CT	cycle threshold
DMEM	Dulbecco’s modified Eagle medium
DMSO	dimethyl sulfoxide
DNA	deoxyribonucleic acid
DTT	dithiothreitol
ECL	electrochemiluminescence
ECM	extracellular matrix
EDTA	ethylenediaminetetraacetic acid
ELISA	enzyme-linked immunosorbent assay
EPB	extensor pollicis brevis
EU	European Union
EU	endotoxin unit
FACS	fluorescence-activated cell sorting
FASP	filter-aided sample preparation
FBS	fetal bovine serum
FC	fold change
FCR	flexor carpi radialis
FDR	false discovery rate
FE002	clinical grade primary progenitor cell sources
GLP	good laboratory practices
GMP	good manufacturing practices
HA	hyaluronic acid
HRP	horseradish peroxidase
IPC	in-process control
kDa	kiloDaltons
kGy	kiloGray
KPP	key process parameter
KQA	key quality attribute
LC-MS/MS	liquid chromatography tandem mass spectrometry
LE	lateral epicondylitis
MCB	master cell bank
MD	medical device
MoA	mechanism of action
MOPS	3-(N-Morpholino)propanesulfonic acid
MRI	magnetic resonance imaging
MSC	mesenchymal stem cell
MTT	3-(4,5-dimethylthiazol-2-yl)-2,5-diphenyltetrazolium bromide
NA	not applicable
NAT	nucleic acid amplification technique
OD	optical density
PAF	paraformaldehyde
PBS	phosphate-buffered saline
PCB	parental cell bank
PDGF-BB	platelet-derived growth factor-BB
Ph. Eur.	European Pharmacopoeia
PMSF	phenylmethanesulfonyl fluoride
PPC	post-process control
PRP	platelet-rich plasma
PTRCT	partial-thickness rotator cuff tears
QA	quality assurance
QC	quality control
qPCR	quantitative polymerase chain reaction
RH	relative humidity
RIPA	radio-immunoprecipitation assay
SDS	sodium dodecyl sulfate
TrSt	standardized transplant
UCL	ulnar collateral ligament
UK	United Kingdom
USA	United States of America
UVEC	umbilical vein endothelial cell
Vs	vs.
WCB	working cell bank
