# Supplementary material for: Bio-Enhanced Neoligaments Graft Bearing FE002 Primary Progenitor Tenocytes: Allogeneic Tissue Engineering & Surgical Proofs-of-Concept for Hand Ligament Regenerative Medicine
Source: Pharmaceutics. 2023 Jul 3;15(7):1873. doi: 10.3390/pharmaceutics15071873 (PMC10385025; doi:10.3390/pharmaceutics15071873)
Supplement: Supplementary file 1 [file pharmaceutics-15-01873-s001.zip › Supplementary Materials.pdf]

pharmaceutics MDPI

Supplementary Materials: Bio-Enhanced Neoligaments Graft Bearing FE002 Primary Progenitor Tenocytes: Allogeneic Tissue Engineering & Surgical Proofs-of-Concept for Hand Ligament Regenerative Medicine

Annick Jeannerat, Joachim Meuli, Cédric Peneveyre, Sandra Jaccoud, Michèle Chemali, Axelle Thomas, Zhifeng Liao, Philippe Abdel-Sayed, Corinne Scaletta, Nathalie Hirt-Burri, Lee Ann Applegate, Wassim Raffoul and Alexis Laurent

1. Supplementary Figures

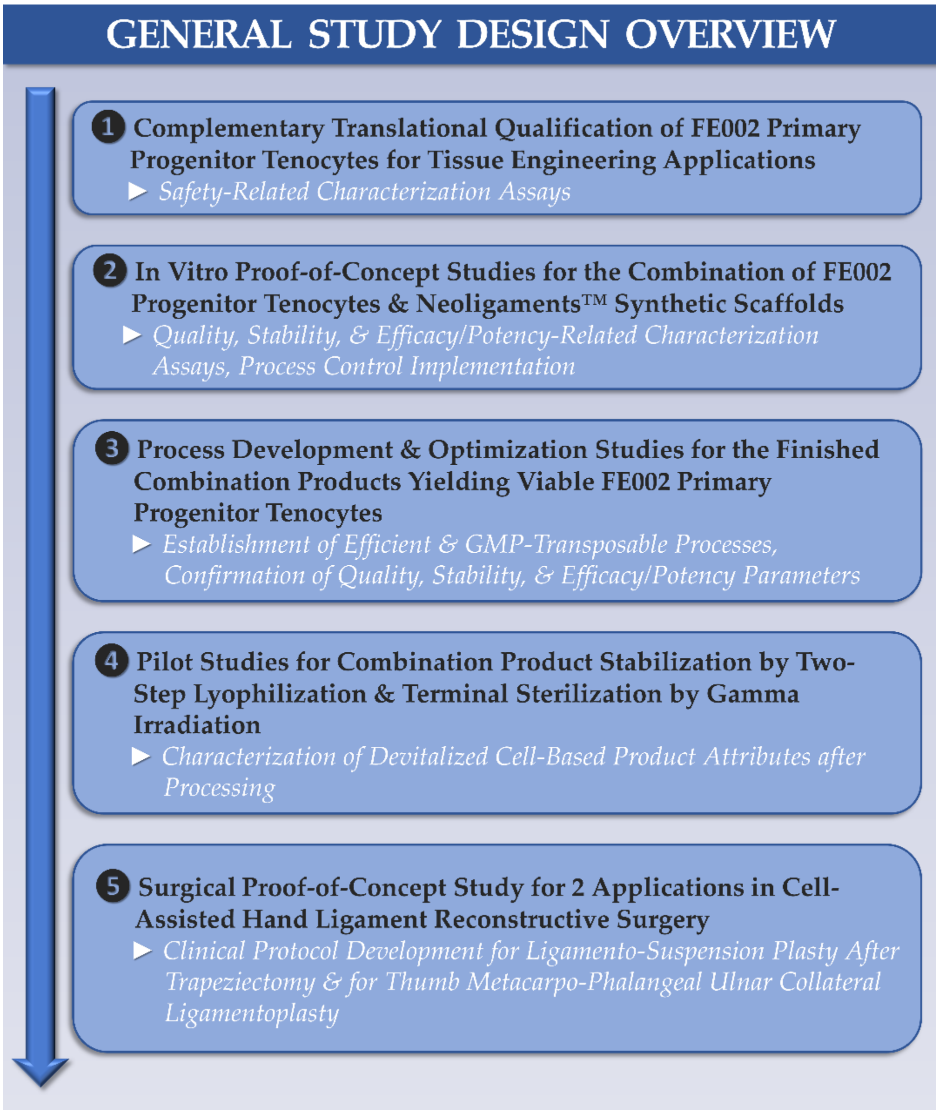

Figure S1A. General overview of the design of the study. (❶) The first step enabled to confirm that the clinical grade FE002 primary progenitor tenocytes of interest were well-adapted for bioengineering purposes, notably from the preclinical safety point-of-view. (❷) The second step enabled to confirm that the considered FE002 primary progenitor tenocytes could be combined in vitro with Neoligaments scaffolds and could deploy their intended mechanisms of action. (❸) The third step enabled to establish optimized process parameters and technical specifications, in view of producing the considered finished combination products under GMP manufacturing standards. (❹) The fourth step enabled to preliminarily assess the potential for temperature stabilization and terminal sterilization of the combination products, for potential off-the-shelf availability of a devitalized cell-based or cell-free product. (❺) The fifth and last step enabled to establish clinical protocols for the use of the considered combination products in specific cell-assisted hand ligament reconstructive surgeries. GMP, good manufacturing practices.

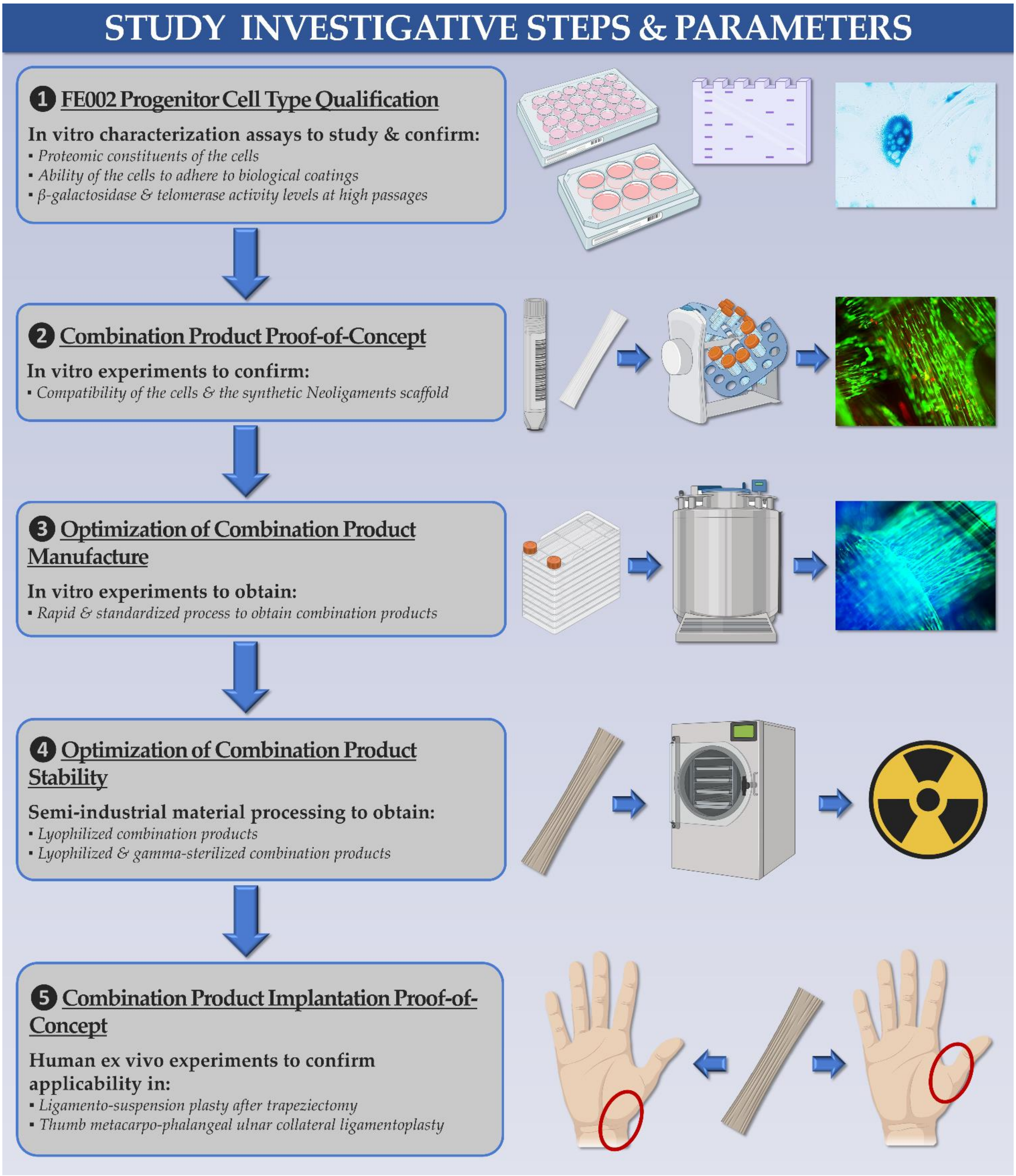

Figure S1B. Illustrated study design, with specification of the investigative steps and the related parameters. (❶) Original in vitro data was gathered to characterize the composition and the behavior of the considered FE002 primary progenitor tenocyte source. (❷) Validation work showed that the cellular components and the scaffold component of the considered combination product are compatible. (❸) Validation work showed that the considered combination products could be obtained for clinical application using simple methods and reasonable technical resources. (❹) Exploratory experiments indicated that two-step lyophilization and gamma irradiation processes do not completely destroy the scaffold and the associated biological materials. (❺) The clinical protocol establishment study confirmed that the considered graft could be used in two indications of hand ligament repair and/or replacement.

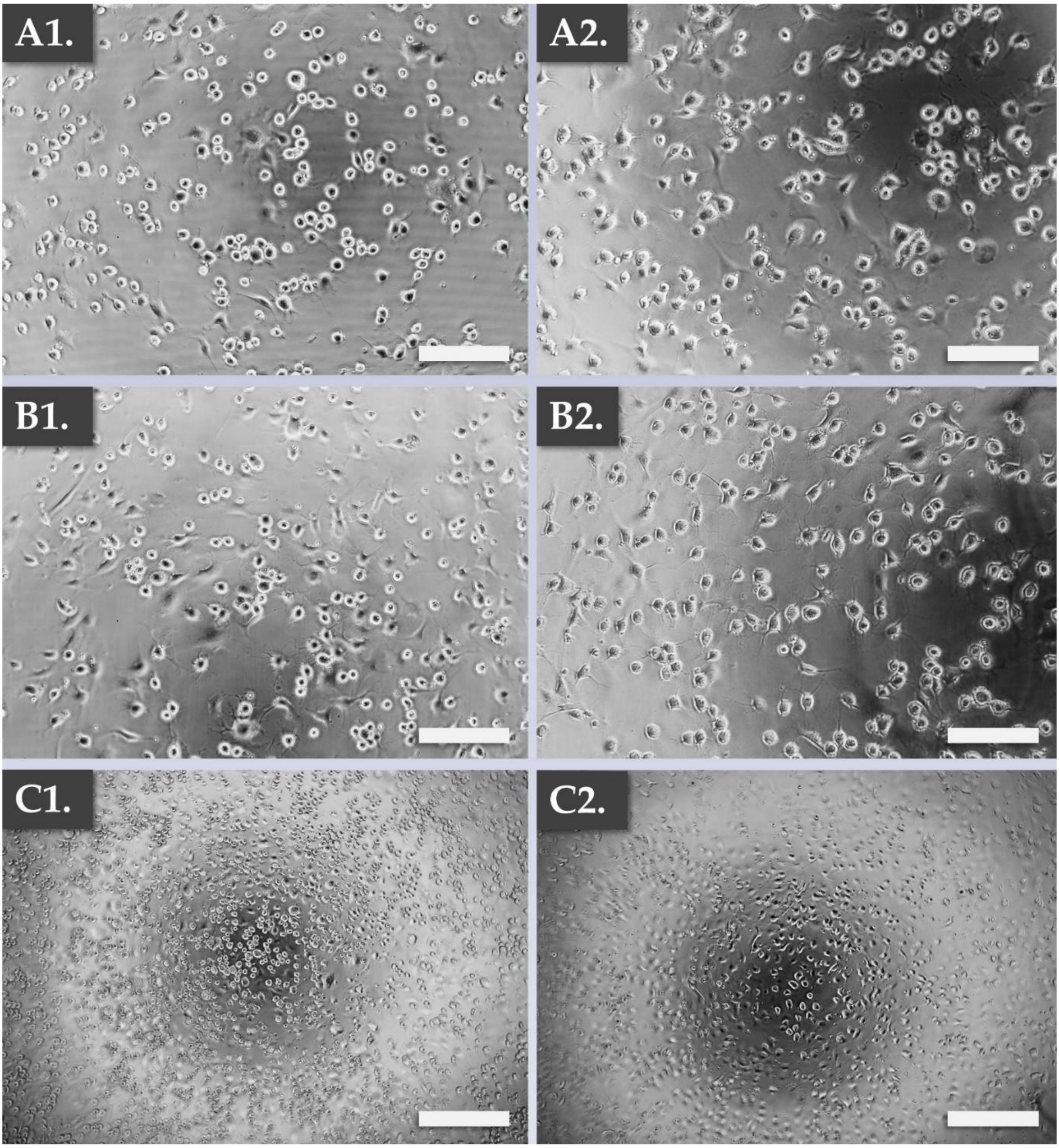

Figure S2. Results of an in vitro cellular adhesion assay aiming to verify the adhesion capacities of primary tenocytes on extracellular matrix components. (**A**) Adhesion results for FE002 primary progenitor tenocytes and patient primary tenocytes on collagen I-coated surfaces, respectively. Scale bars = 50 µm. (**B**) Adhesion results for FE002 primary progenitor tenocytes and patient primary tenocytes on FBS-coated surfaces, respectively. Of note, FBS is known to contain an array of proteins of interest for cellular adhesion (e.g., vitronectin). Scale bars = 50 µm. (**C**) Adhesion results for FE002 primary progenitor tenocytes and patient primary tenocytes on fibronectin-coated surfaces, respectively. Scale bars = 150 µm. Overall, the experimental results indicated that both primary cell types were capable of adhering to extracellular matrix components in vitro and that the adhesion behaviors of both primary cell types were assessed as being similar. FBS, fetal bovine serum.

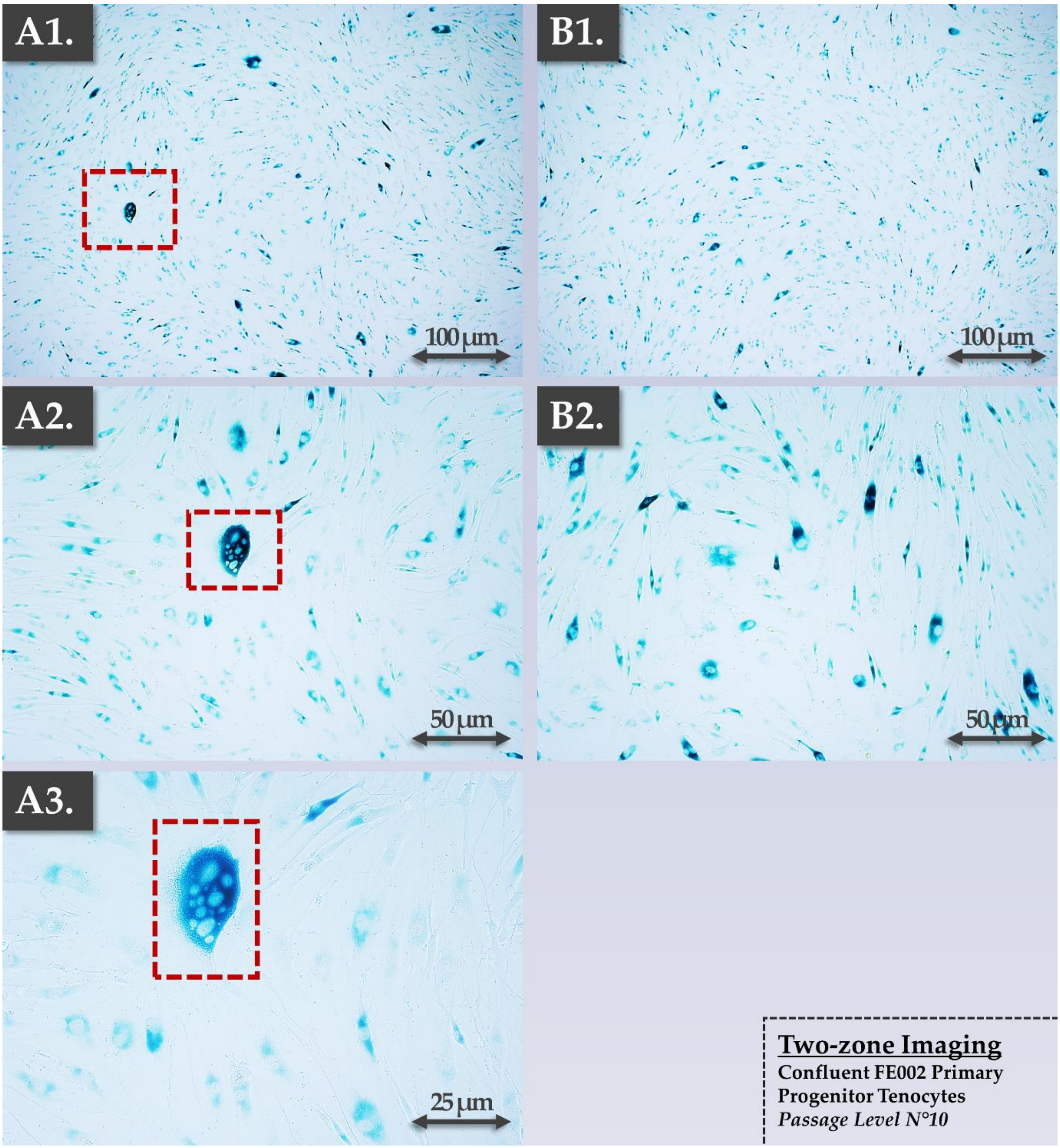

Figure S3. Results of a β-galactosidase staining assay of FE002 primary progenitor tenocytes at a late in vitro passage level (i.e., passage level N°10, usually 3–4 in vitro passage levels above those of interest for clinical cytotherapeutic use). At this passage level, most of the cells (i.e., > 90%) were found to be positive for β-galactosidase activity and the in vitro cellular proliferation potential was substantially reduced. Specifically, an increase in β-galactosidase-positive cell contents was observed with each incremental in vitro cell passage level, along with an increase in cell population doubling times. These results confirmed that the considered FE002 primary progenitor tenocytes were characterized by a finite in vitro lifespan, thereby limiting the risk of uncontrolled proliferation and tumor formation in vivo. Such results were further confirmed by the recorded drop in cell population doubling numbers and the rapid rise in cell population doubling times for cultured cells at high passage levels (i.e., passage levels > 9 in the retained manufacturing system and under ad hoc technical specifications). (**A1**,**B1**) Microscopy photographic records of β-gal-stained cells under 10× optical enlargement. Scale bars = 100 µm. (**A2**,**B2**) Microscopy photographic records of β-gal-stained cells under 20× optical enlargement. Scale bars = 50 µm. (**A3**) Microscopy photographic records of β-gal-stained cells under 40× optical enlargement. Scale bars = 25 µm. In panels **A1**–**A3**, a degranulating and vacuole-filled progenitor cell is evidenced in a red outline.

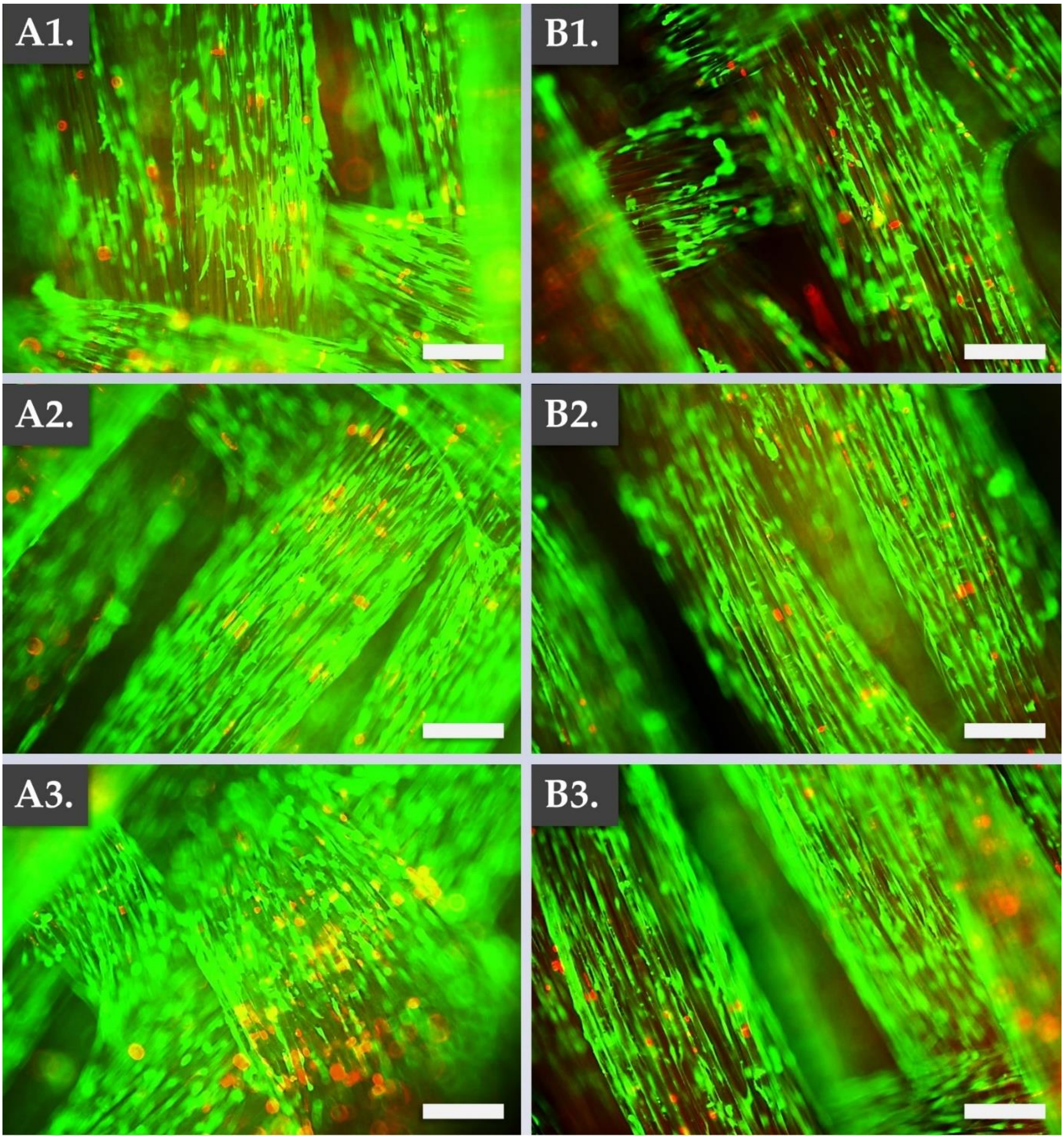

Figure S4. Illustrative results of cytocompatibility studies, using two types of primary tenocytes and plasma-treated Infinity-Lock 3 scaffolds, as assessed by Live-Dead cell imaging following 10 days of combination product incubation. Both primary cell types were shown to adhere and to proliferate throughout the scaffolds. At the end of the incubation period, a vast majority of cells were found to be viable and had adopted a highly organized structure within the scaffold, with tight cellular alignment along the scaffold fibers. (**A1**–**A3**) Various zones of constructs bearing patient primary tenocytes. These results verify the intended (i.e., original) passive purpose of the scaffold itself, namely the provided possibility for patient cells to progressively colonize the scaffold. Scale bars = 150 µm. (**B1**–**B3**) Various zones of constructs bearing FE002 primary progenitor tenocytes. These results confirm the new intended purpose of the scaffold within the combination product, namely the carrying of the therapeutic cellular payload on a functionalized (i.e., bio-enhanced) construct. Scale bars = 150 µm.

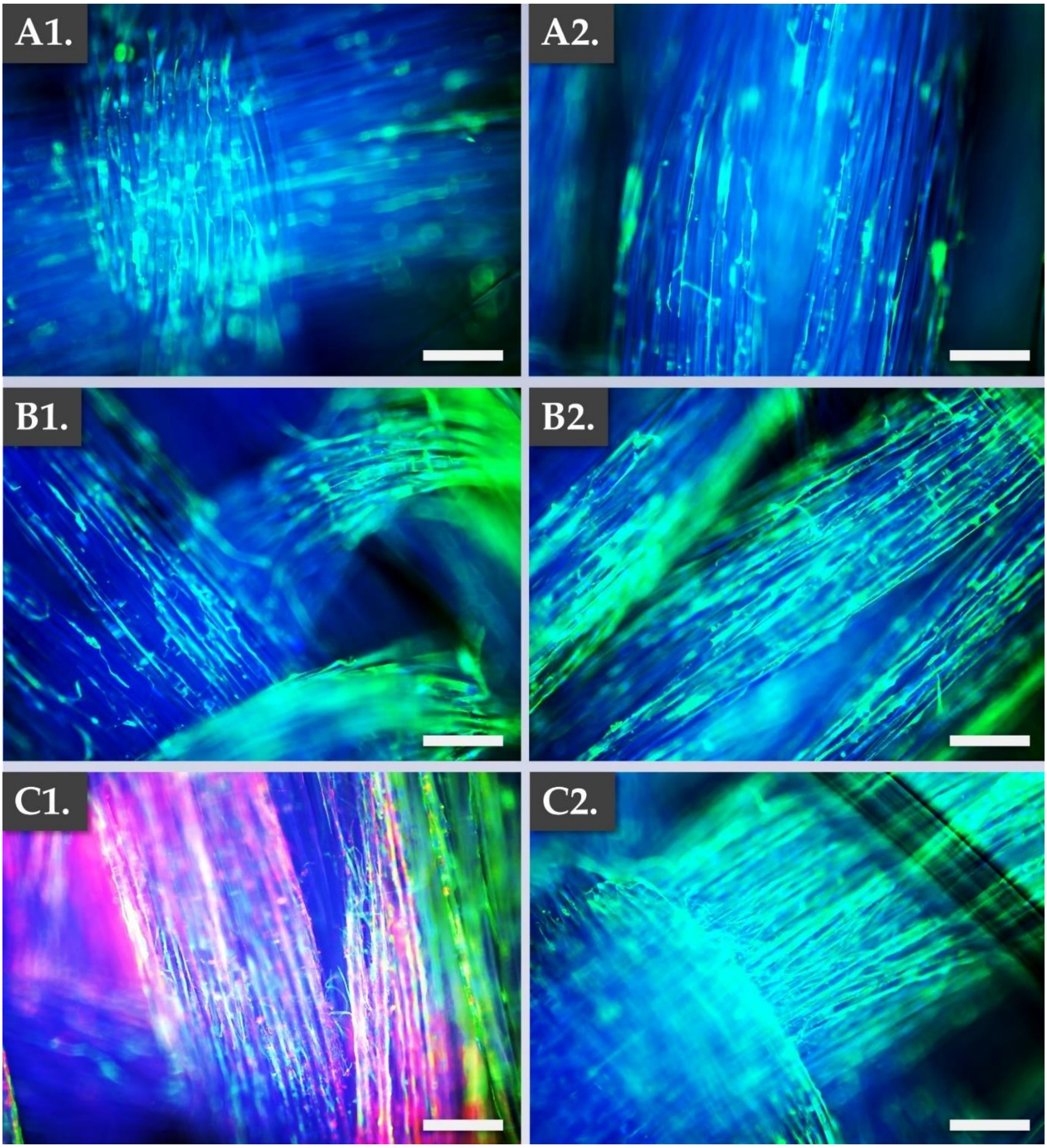

Figure S5. Immunofluorescence data excerpts showing extracellular matrix synthesis and deposition along the Infinity-Lock 3 scaffold fibers by the FE002 primary progenitor tenocytes of interest. The combination products were incubated during twenty-one days before endpoint processing and imaging. (**A1**) Imaging of decorin on a non-plasma-treated scaffold. Scale bar = 150 μm. (**A2**) Imaging of decorin on a plasma-treated scaffold. Scale bar = 150 μm. (**B1**) Imaging of tenomodulin on a non-plasma-treated scaffold. Scale bar = 150 μm. (**B2**) Imaging of tenomodulin on a plasma-treated scaffold. Scale bar = 150 μm. (**C1**) Imaging of aggrecan and phalloidin on a non-plasma-treated scaffold. Scale bar = 150 μm. (**C2**) Imaging of tenomodulin on a plasma-treated scaffold. Scale bar = 150 μm.

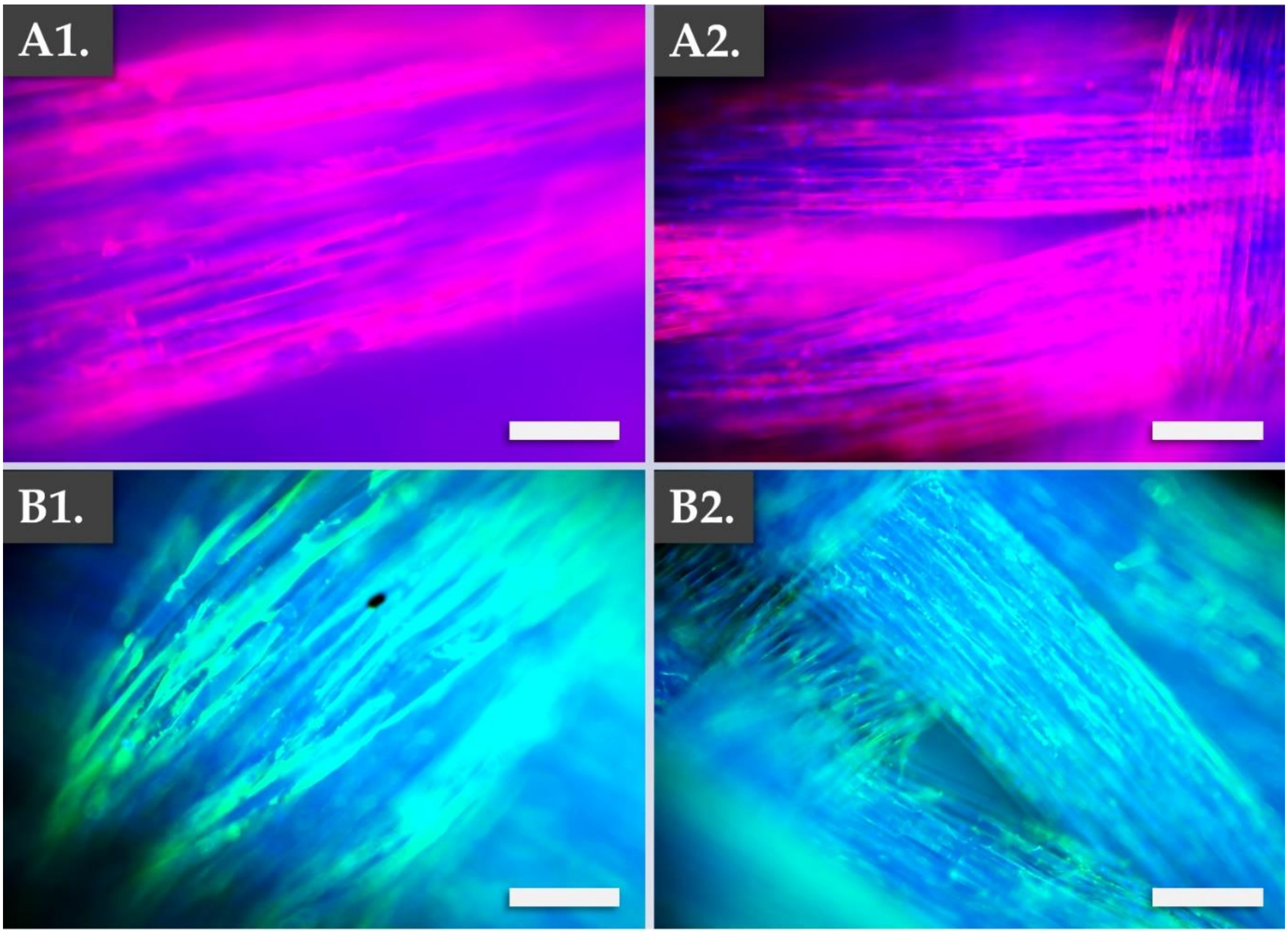

Figure S6. Immunofluorescence data excerpts showing extracellular matrix synthesis and deposition along the scaffold (i.e., plasma-treated Infinity-Lock 3) fibers by the patient primary tenocytes. The combination products were incubated during 28 days before endpoint processing and imaging. (**A1**) Imaging of phalloidin and Hoechst stain. Scale bar = 100 µm. (**A2**) Imaging of phalloidin and Hoechst stain. Scale bar = 200 µm. (**B1**) Imaging of aggrecan and Hoechst stain. Scale bar = 200 µm. (**B2**) Imaging of aggrecan and Hoechst stain. Scale bar = 200 µm.

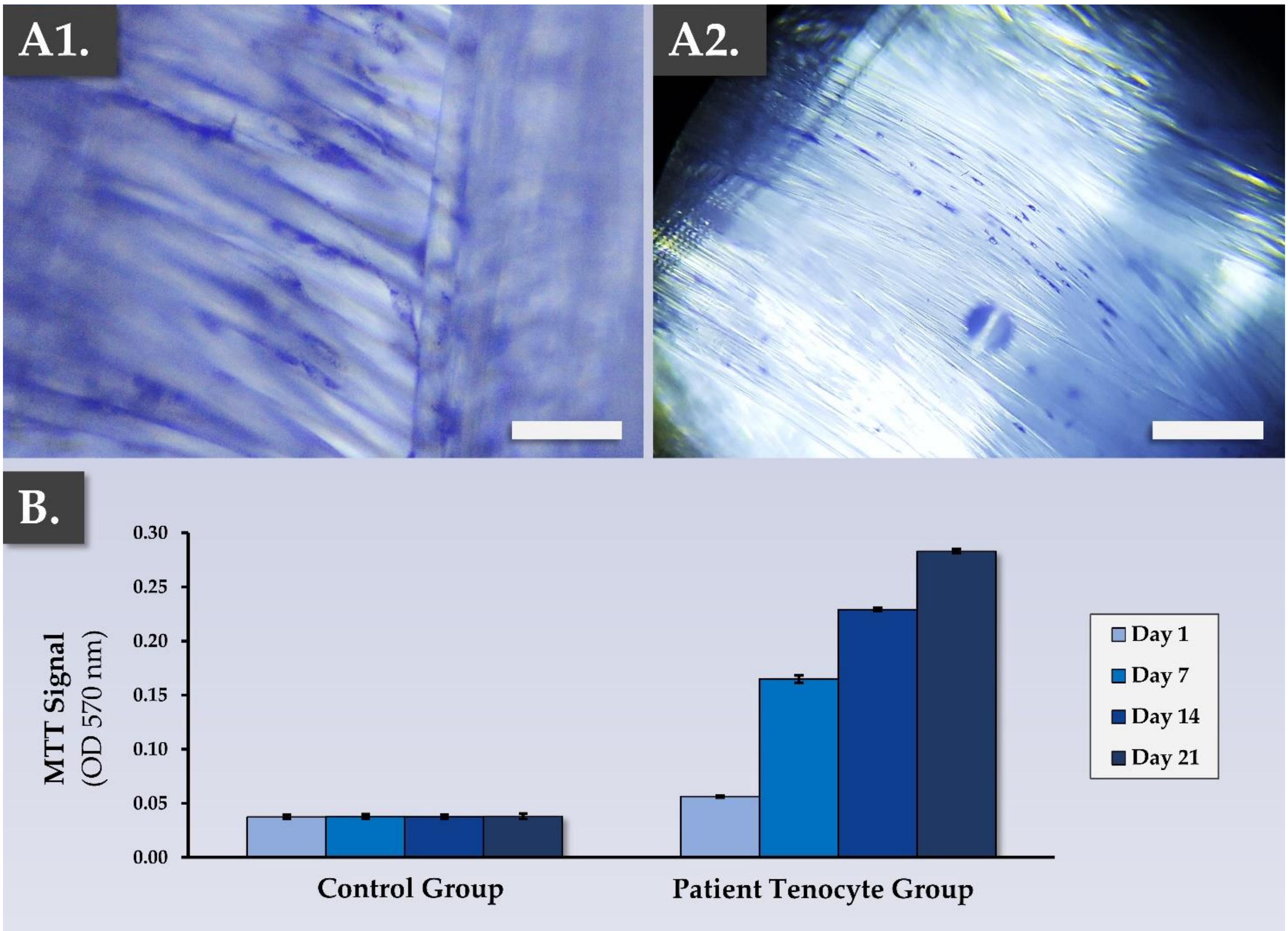

Figure S7. Illustrative excerpts of cytocompatibility studies on plasma-treated Infinity-Lock 3 scaffolds using patient primary tenocytes. (**A1**,**A2**) Imaging of patient tenocytes on the scaffold fibers, with MTT staining at an early timepoint following cell seeding. The cells adhered to the scaffold and were aligned along the fibers. Scale bar = 50 µm and 100 µm, respectively. (**B**) Iterative MTT quantification after dynamic cell seeding of the scaffolds with the patient primary tenocytes. The data shows that the patient tenocytes proliferate over time on the scaffold during the 21 days of incubation, which is confirmed visually by the MTT stains (i.e., visual assessment of progressive scaffold colonization, with progressively enhanced homogeneity of cellular coverage). OD, optical density.

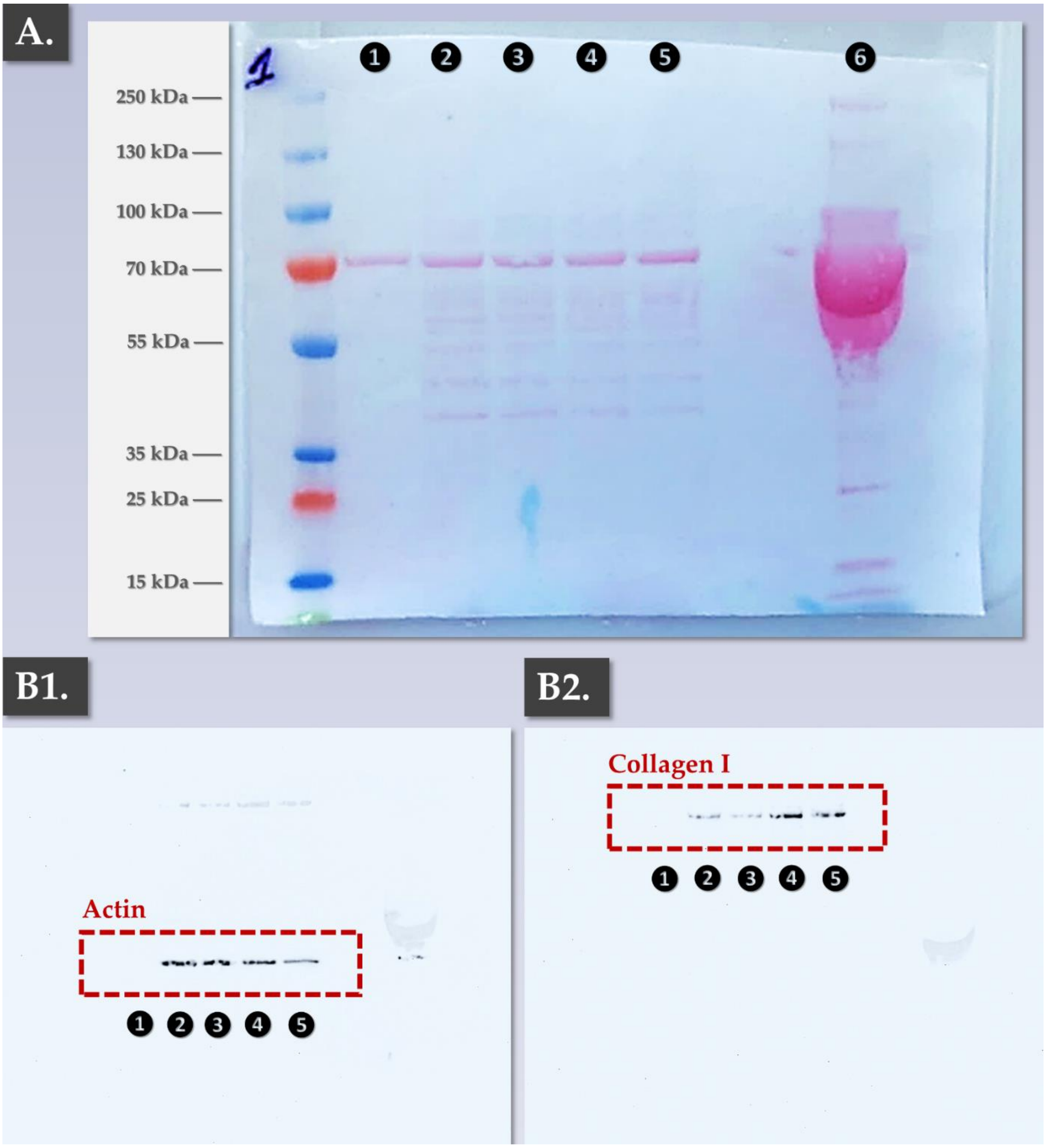

Figure S8. Whole gel data presentation relative to the Western blot data excerpts included in Figure 2, with focus on collagen I. (**A**) Imaging of the gel following Ponceau staining, along with an annotated molecular weight ladder relative to the collagen I gel. The gel was prepared with samples derived from constructs incubated without any cells (i.e., channel number 1), samples from constructs incubated with patient primary tenocytes at a seeding dose of 5 × 10^4 cells/cm (i.e., channel number 2), samples from constructs incubated with patient primary tenocytes at a seeding dose of 2.5 × 10^4 cells/cm (i.e., channel number 3), samples from constructs incubated with FE002 primary progenitor tenocytes at a seeding dose of 5 × 10^4 cells/cm (i.e., channel number 4), samples from constructs incubated with FE002 primary progenitor tenocytes at a seeding dose of 2.5 × 10^4 cells/cm (i.e., channel number 5), and samples containing complete cell culture medium only (i.e., channel number 6, large blot representing BSA). Ponceau staining revealed that a band in all of the samples migrated at the same level as the major band corresponding to the cell culture medium. This demonstrated that some proteins from the cell culture medium can bind to the scaffold. (**B1**) Western blot for actin. Exposure time = 2 min. (**B2**) Western blot for collagen I. Exposure time = 2 min. BSA, bovine serum albumin; kDa, kiloDalton; min, minute.

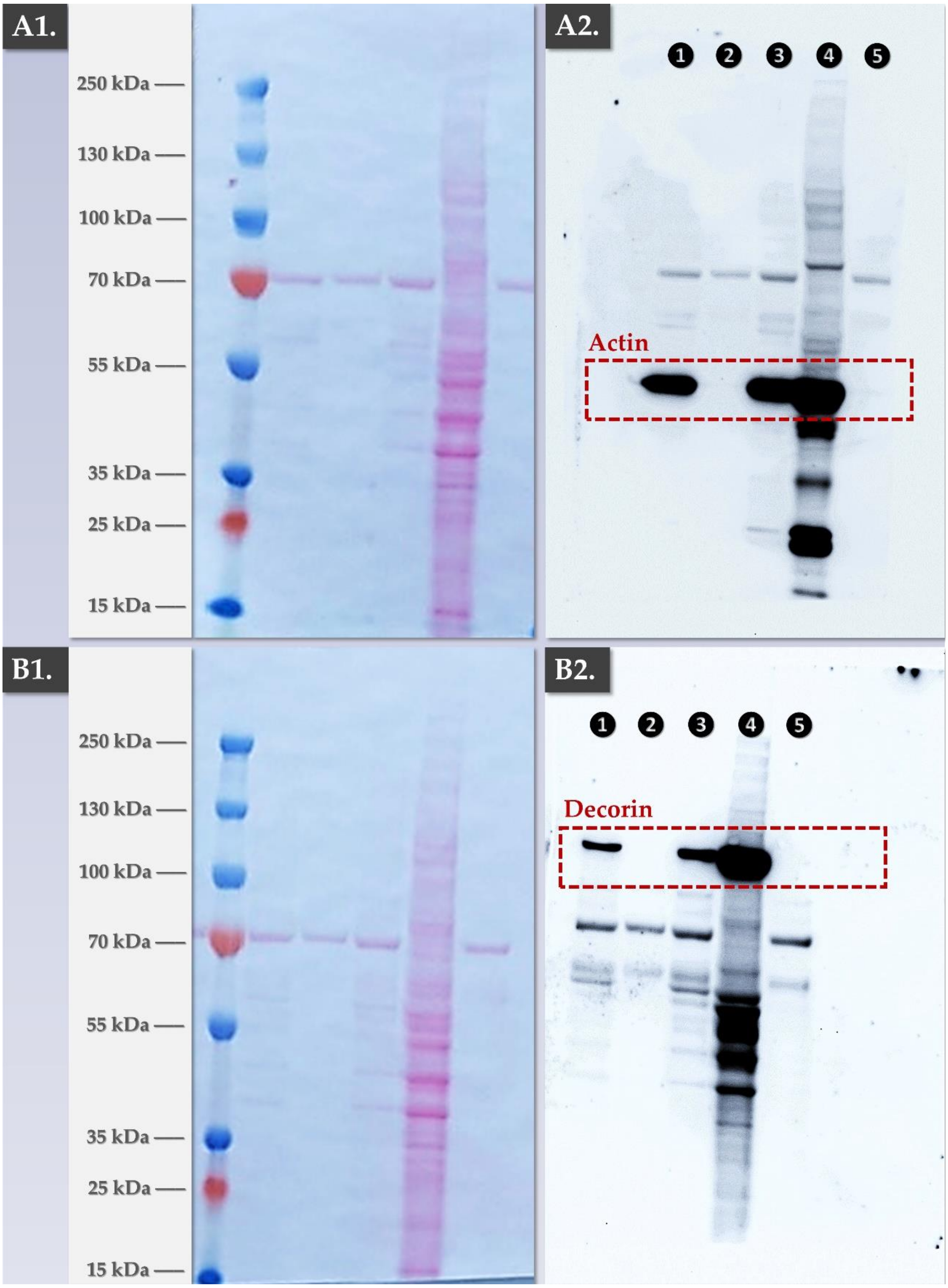

Figure S9. Whole gel data presentation relative to the Western blot data excerpts included in Figure 3, with focus on decorin and actin. (**A1**) Annotated molecular weight ladder relative to the actin gel, as recorded on the Ponceau staining. (**A2**) Western blot for actin, as revealed using ECL, prepared with samples derived from non-plasma-treated scaffolds incubated with FE002 primary progenitor tenocytes at a seeding dose of 2.5 × 10^4 cells/cm (i.e., channel number 1), samples from plasma-treated scaffolds incubated without cells (i.e., channel number 2), samples from plasma-treated scaffolds incubated with FE002 primary progenitor tenocytes at a seeding dose of 2.5 × 10^4 cells/cm (i.e., channel number 3), samples from FE002 primary progenitor tenocyte lysate (i.e., channel number 4), and samples from non-plasma-treated scaffolds incubated without cells (i.e., channel number 5). Actin was expected at 42 kDa. Exposure time = 30 s. (**B1**) Annotated molecular weight ladder relative to the decorin gel, as recorded on the Ponceau staining. (**B2**). Western blot for decorin, as revealed using ECL, prepared with the same sample order as for the actin gel. Decorin was expected between 100 kDa and 140 kDa. Exposure time = 2 min. ECL, electrochemiluminescence; kDa, kiloDalton; min, minute.

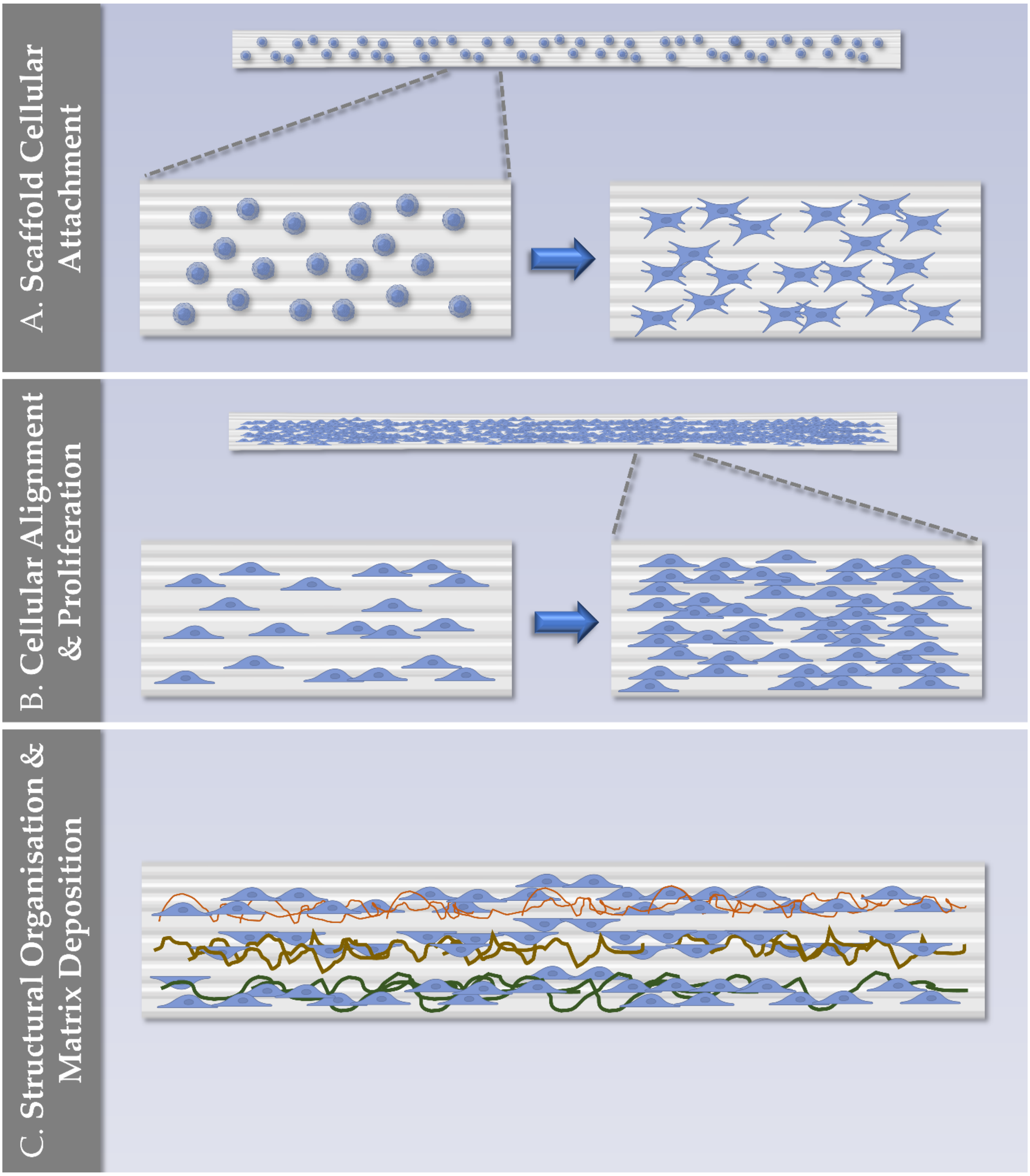

Figure S10. Illustrated schematic overview of the observed behaviors of the FE002 primary progenitor tenocytes on the synthetic Infinity-Lock 3 scaffolds. (**A**) During dynamic cell seeding of the scaffolds, the FE002 primary progenitor cells are distributed along the fibers and start to adhere throughout the scaffold. (**B**) During the static incubation phase following dynamic cell seeding, the FE002 primary progenitor tenocytes align themselves along the scaffold fibers to form a structurally organized cell population. Cellular proliferation along the fibers results in a progressively denser cellular population throughout the scaffold, with enhanced cellular alignment along the fibers. (**C**) Following cellular adhesion on the scaffold, extracellular matrix synthesis and deposition activities take place, further enhancing the structural organization of the biological materials in the bio-enhanced finished product. It is probable that both the cellular proliferation and the extracellular matrix deposition activities start in parallel following cellular adhesion on the scaffold and are not purely sequential.

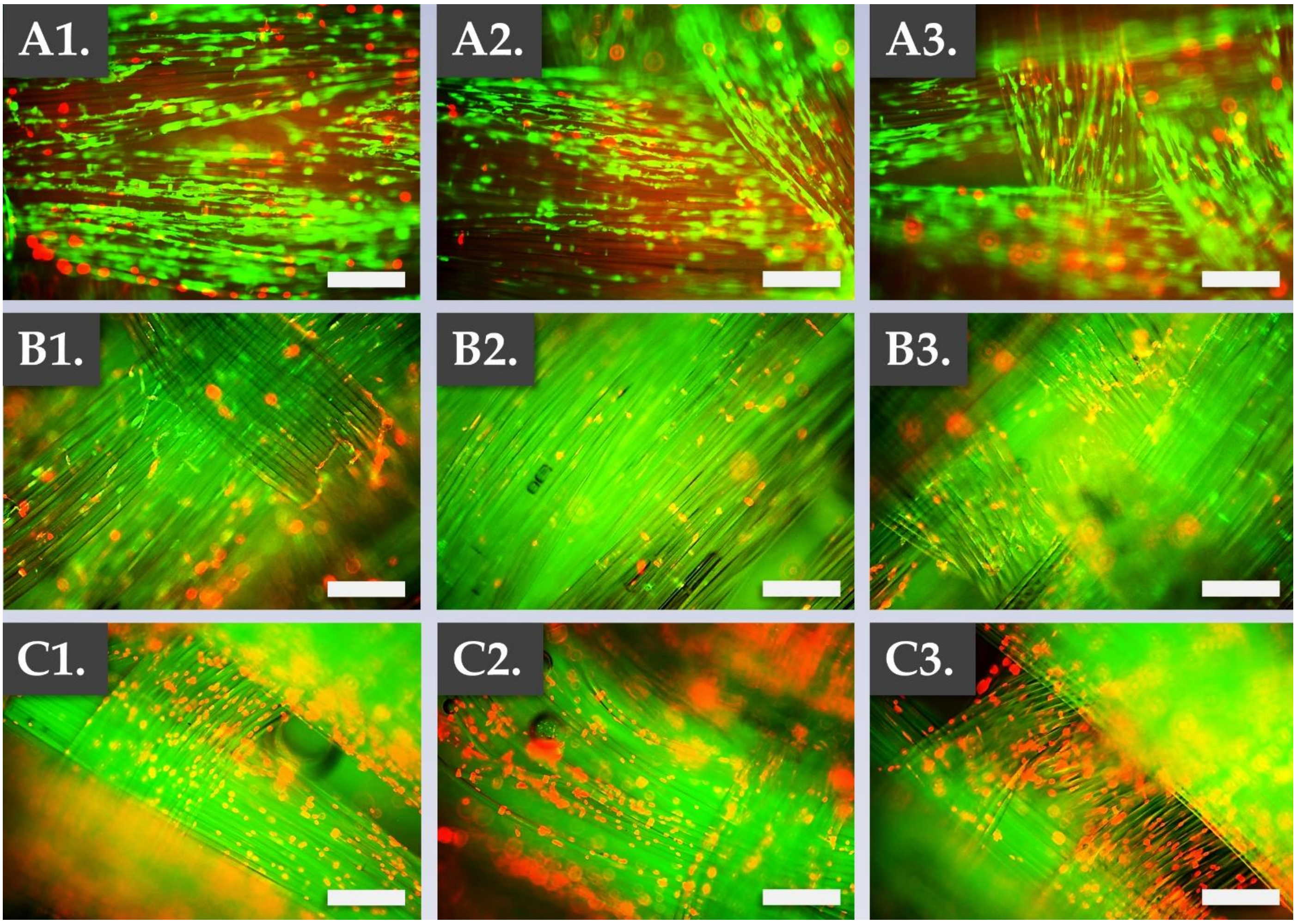

Figure S11. Graphical results of iterative Live-Dead assays used to evaluate the effects of combination product lyophilization and terminal sterilization by γ-irradiation. Pilot experiments were performed to assess whether further processing of the fresh finished products would remove all FE002 progenitor cellular materials from the Infinity-Lock 3 scaffolds, potentially resulting in a decellularized fibrous structure. (**A1–A3**) Live-Dead results of freshly harvested combination products after ten days of incubation. The majority of the FE002 primary progenitor tenocytes were found to be viable (i.e., in green fluorescence) and aligned along the fibers of the scaffold. Scale bars = 200 µm. (**B1–B3**) Live-Dead results of the combination products following two-step lyophilization for temperature stabilization. Post-lyophilization, cellular alignment on the fibers was less evident than for fresh finished products, and less cells were generally visible on the scaffolds. However, significant amounts of FE002 progenitor cellular materials were still present on the lyophilized combination products. Scale bars = 200 µm. (**C1–C3**) Live-Dead results of the combination products following two-step lyophilization and terminal γ-sterilization (i.e., irradiation dose of 25–30 kGy from a ^{60}Co source). Cellular alignment on the fibers was less evident than for fresh finished products. However, significant amounts of FE002 progenitor cellular materials were still present on the lyophilized and sterilized combination products. Scale bars = 200 µm. This aspect confirmed the resistance of cellular structures to γ-irradiation (i.e., presence of biological materials) and partial conservation of adhesion capacities to the scaffold (i.e., localization of cellular materials on the scaffold). It should be noted that for the lyophilized and the lyophilized/irradiated sample groups, interpretation of the Live-Dead fluorescence signals is drastically limited and should only be considered relevantly from a biological material presence/localization point-of-view. Specifically, green fluorescence signals represent the presence of active intracellular esterase enzyme in a viable cell population. While lyophilization processing probably preserved the activity of esterase (i.e., explaining the residual green signal), the cells were confirmed to be totally devitalized by Trypan blue staining. By extension, it is probable that the increased amount of red fluorescence signal in the lyophilized/irradiated sample group corresponded to the inactivation of esterase following sterilization processing. Such results prompted the experimental investigation of alternative parameters/attributes for the evaluation of the impacts of lyophilization and γ-irradiation (e.g., protein synthesis and deposition throughout the scaffold), as described hereafter. Overall and despite significant observable effects of combination product processing on the cellular materials of interest, the presented results did not technically exclude the possibility of obtaining temperature-stable and terminally sterilized finished products bearing FE002 progenitor biological materials. kGy, kiloGray.

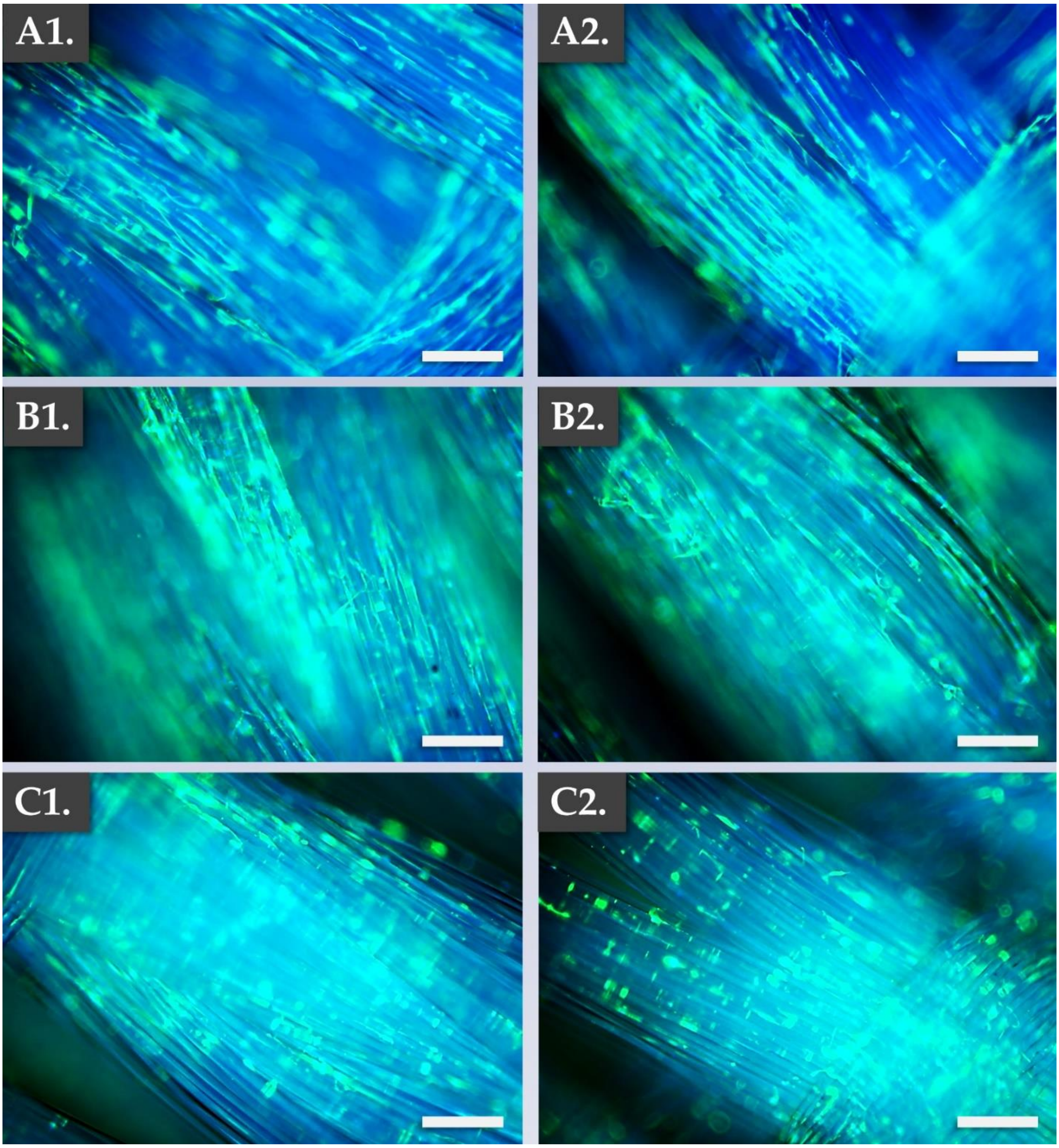

Figure S12. Graphical results of iterative immunofluorescence assays used to evaluate the effects of combination product lyophilization and terminal γ-sterilization. The experiments were performed to assess whether further processing of the fresh finished products would remove all biological materials (i.e., synthesized and deposited FE002 primary progenitor extracellular matrix components) from the scaffolds. (**A1**,**A2**) Imaging of aggrecan on a freshly harvested finished product. Scale bars = 200 µm. (**B1**,**B2**) Imaging of aggrecan on a lyophilized finished product. Post-lyophilization, the majority of the deposited extracellular matrix components were found to still be aligned on the scaffold fibers compared to fresh finished products. Scale bars = 200 µm. (**C1**,**C2**) Imaging of aggrecan on a lyophilized and γ-sterilized (i.e., irradiation dose of 25–30 kGy from a ^{60}Co source) finished product. Post-sterilization, extracellular matrix components were found to be less organized (i.e., less alignment on the fibers) compared to freshly harvested finished products. However, significant amounts of extracellular matrix materials were still present on the lyophilized and sterilized combination products. Scale bars = 200 µm. Overall and despite significant observable effects of combination product processing on the extracellular matrix materials of interest, the presented results did not technically exclude the possibility of obtaining temperature-stable and terminally sterilized finished products bearing FE002 progenitor biological materials.

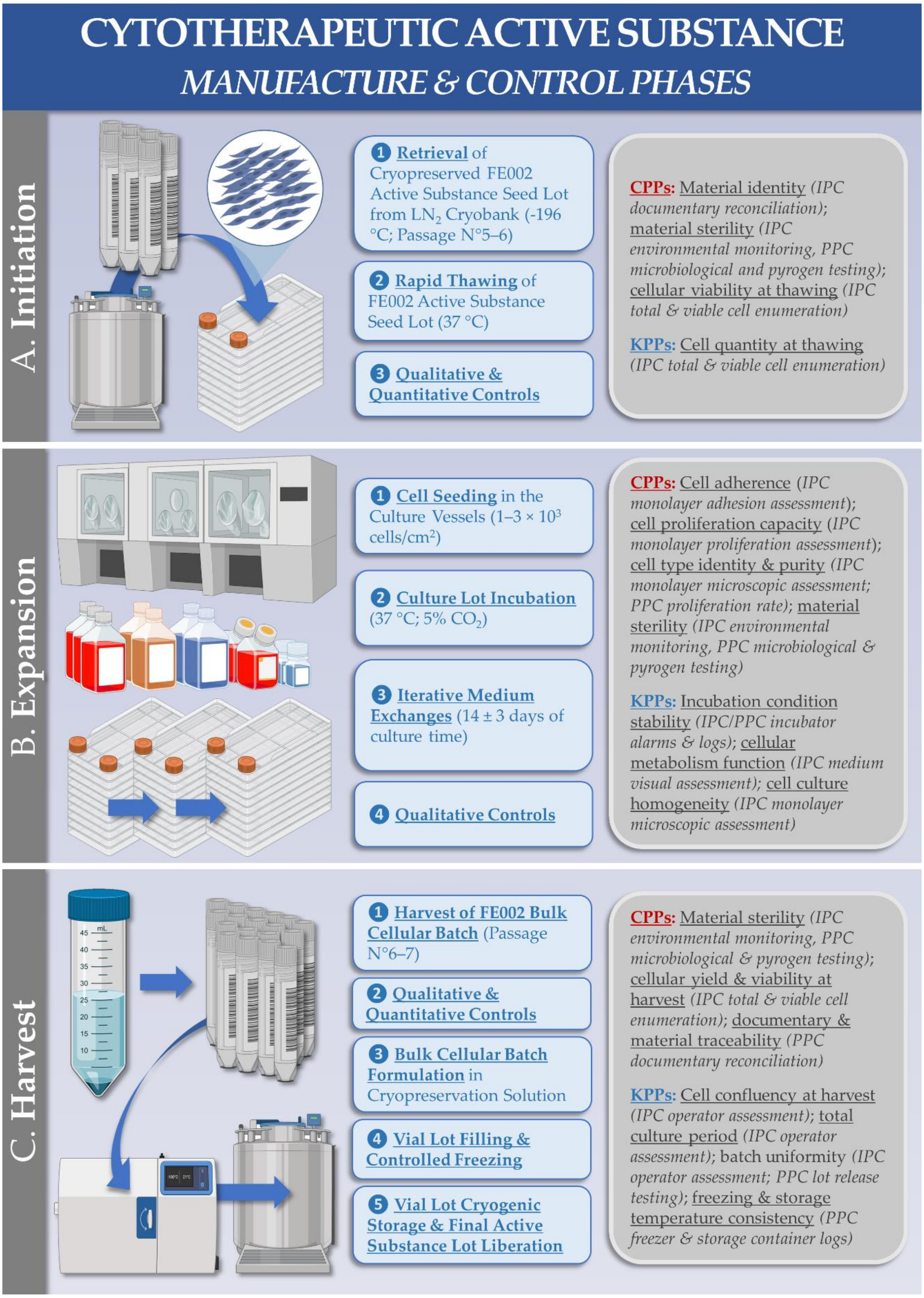

Figure S13. Schematic and parametric description of the manufacturing and control steps performed on the cytotherapeutic active substance (i.e., FE002 primary progenitor tenocytes). For each process phase, major CPP and KPP as well as the related IPC or PPC are specified. The related parameter/control targets and acceptance criteria are detailed in Table S2. The related cellular active substance quality attributes are detailed in Table S4. (**A**) A specific seed lot of FE002 primary progenitor tenocytes is initiated from storage and controlled in view of an in vitro expansion. (**B**) The monolayer expansion phase is performed under standard incubation and diploid primary cell culture maintenance conditions. (**C**) The harvested FE002 cells are used to constitute a cell bank lot and are processed in view of liberation and long-term cryogenic storage. CPP, critical process parameter; IPC, in-process control; KPP, key process parameter; PPC, post-process control.

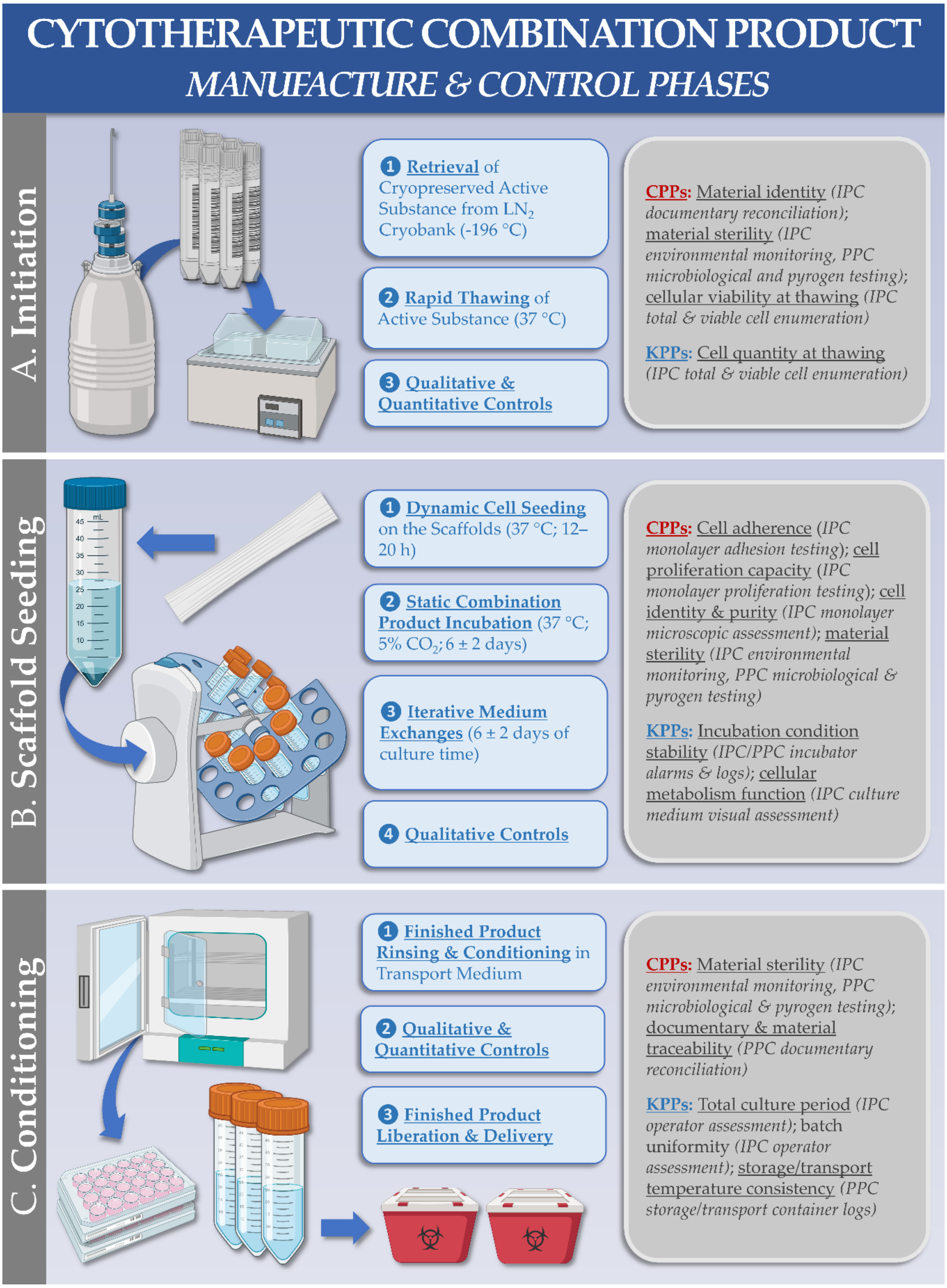

Figure S14. Schematic and parametric description of the manufacturing and control steps performed on the cytotherapeutic combination product (i.e., Neoligaments Infinity-Lock 3 scaffold bearing FE002 primary progenitor tenocytes). For each process phase, major CPP and KPP as well as the related IPC or PPC are specified. The related parameter/control targets and acceptance criteria are detailed in Table S3. The related finished product quality attributes are detailed in Table S5. (**A**) A specific active substance lot of FE002 primary progenitor tenocytes is initiated from storage and controlled in view of Infinity-Lock 3 scaffold seeding. (**B**) Following overnight dynamic cell seeding in an orbital shaker, the scaffolds are incubated to promote cellular alignment along the fibers, cellular proliferation, and extracellular matrix deposition throughout the construct. (**C**) Following finished product harvest and processing, appropriate controls are performed, and products are conditioned for shipment to the clinical site. CPP, critical process parameter; IPC, in-process control; KPP, key process parameter; PPC, post-process control.

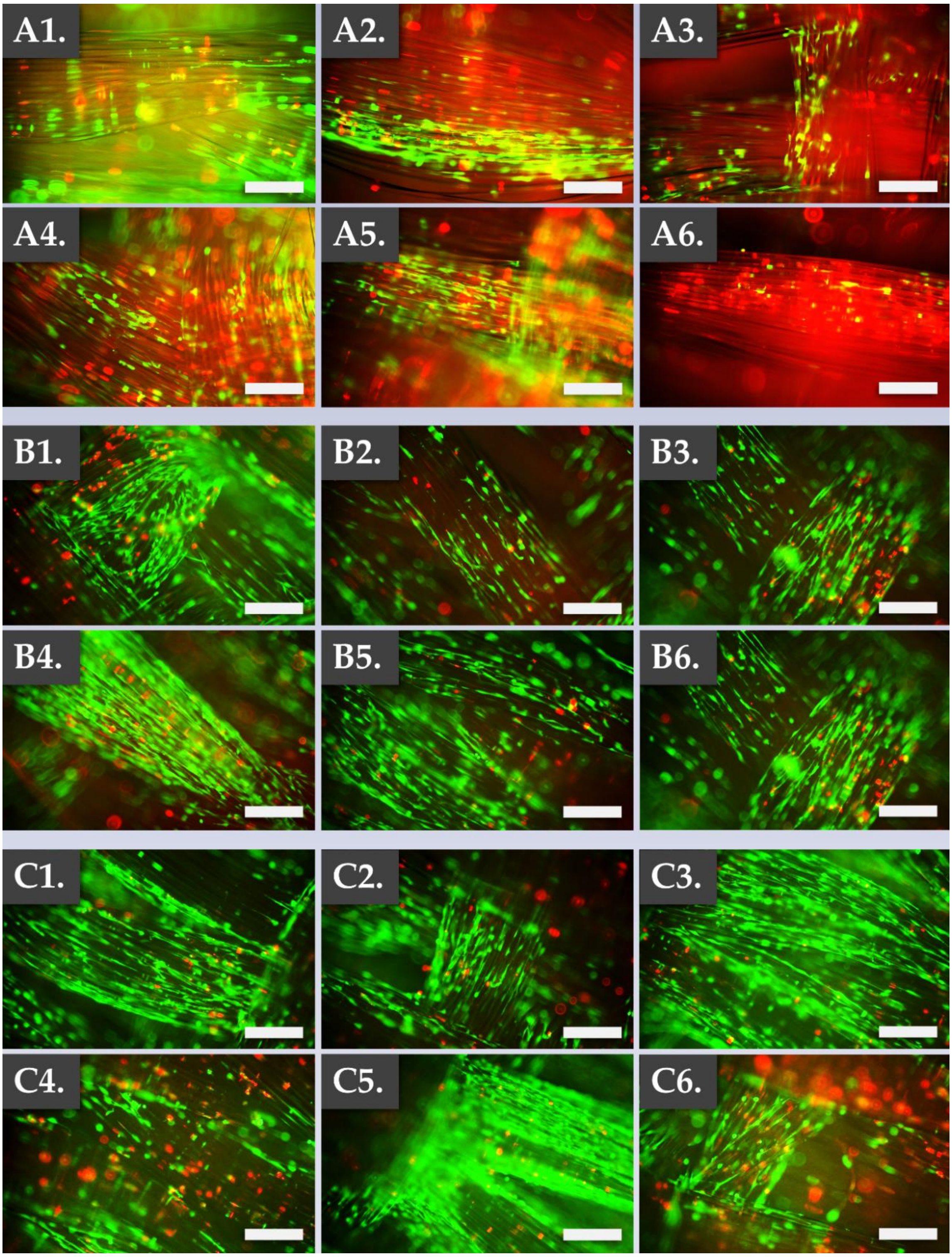

Figure S15. Graphical results of iterative Live-Dead assays used to evaluate the effects of combination product incubation time-period length. Experiments were performed to assess which timepoints resulted in optimal behaviors of the FE002 primary progenitor cells on the Infinity-Lock 3 scaffolds (i.e., cellular adhesion and alignment along the fibers, high proportion of viable cells, cellular proliferation on the scaffold). (**A1**–**A6**) Live-Dead imaging of the freshly harvested combination products after two days of incubation. Cellular adhesion was confirmed, and the cells were found to be in the process of alignment along the fibers of the scaffold. Although most cells were assessed as viable (i.e., in green fluorescence), a significant proportion of cells were assessed as non-viable (i.e., in red fluorescence). Scale bars = 200 µm. (**B1**–**B6**) Live-Dead imaging of the freshly harvested combination products after four days of incubation. A higher level of organization of the cells along the scaffold fibers was noted compared to the two-day timepoint and less non-viable cells were observed. Increased signal intensity confirmed cellular proliferation on the scaffold compared to the two-day timepoint. Scale bars = 200 µm. (**C1**–**C6**) Live-Dead imaging of the freshly harvested combination products after seven days of incubation. The combination products were found to be similar to those from the four-day timepoint, with high structural organization and few non-viable cells. Scale bars = 200 µm.

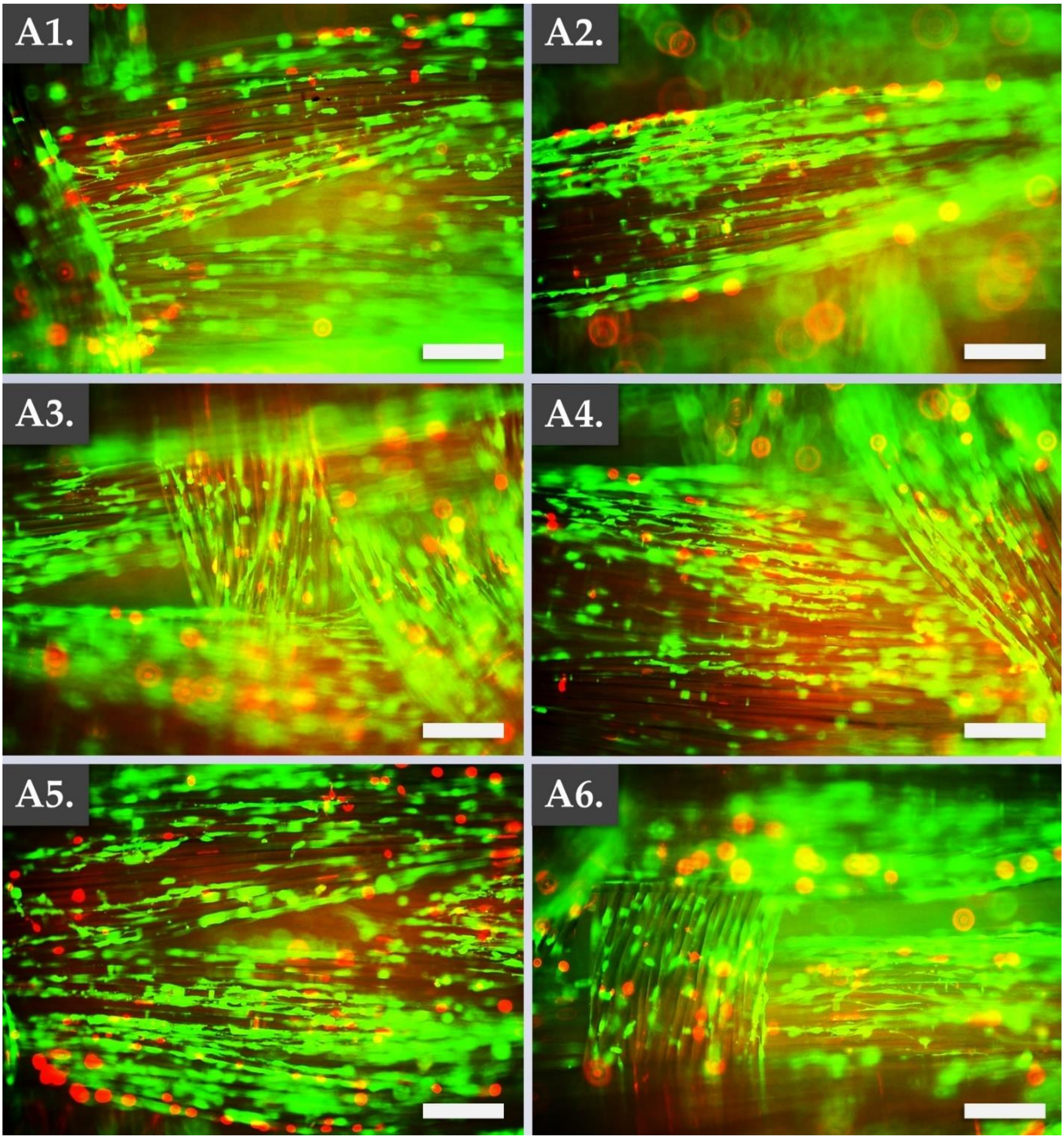

Figure S16. Graphical results of Live-Dead assays used to evaluate the constructs obtained using the optimized finished product manufacturing protocol (i.e., cell seeding dose of 10^5 cells/cm, 7 days of incubation). (**A1**–**A6**) Live-Dead imaging of the freshly harvested combination products (i.e., non-plasma-treated Infinity-Lock 3 scaffolds, seeded with FE002 primary progenitor tenocytes at passage level 7 and in cryopreserved active cellular substance form). The combination products were found to present high structural organization and excellent alignment of the cells along the scaffold fibers. Scale bars = 200 µm.

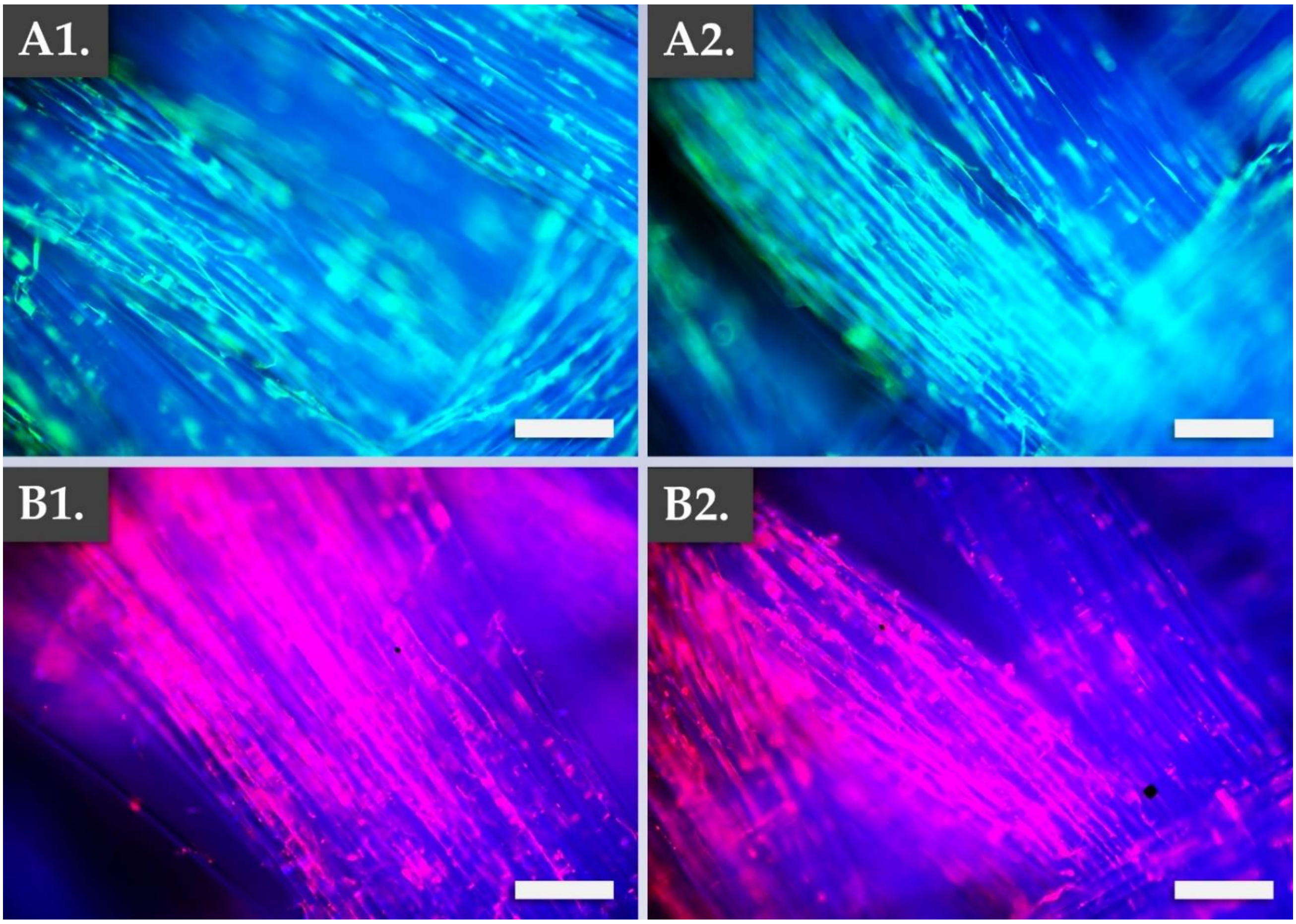

Figure S17. Graphical results of immunofluorescence assays used to evaluate the constructs obtained using the optimized finished product manufacturing protocol (i.e., cell seeding dose of 10^5 cells/cm, 7 days of incubation). (**A1**,**A2**) Imaging of aggrecan on the freshly harvested combination products (i.e., non-plasma-treated Infinity-Lock 3 scaffolds, seeded with FE002 primary progenitor tenocytes at passage level 7 and in cryopreserved active cellular substance form). Scale bars = 200 µm. (**B1**,**B2**) Imaging of phalloidin on the freshly harvested combination products (i.e., non-plasma-treated Infinity-Lock 3 scaffolds, seeded with FE002 primary progenitor tenocytes at passage level 7 and in cryopreserved active cellular substance form). Scale bars = 200 µm.

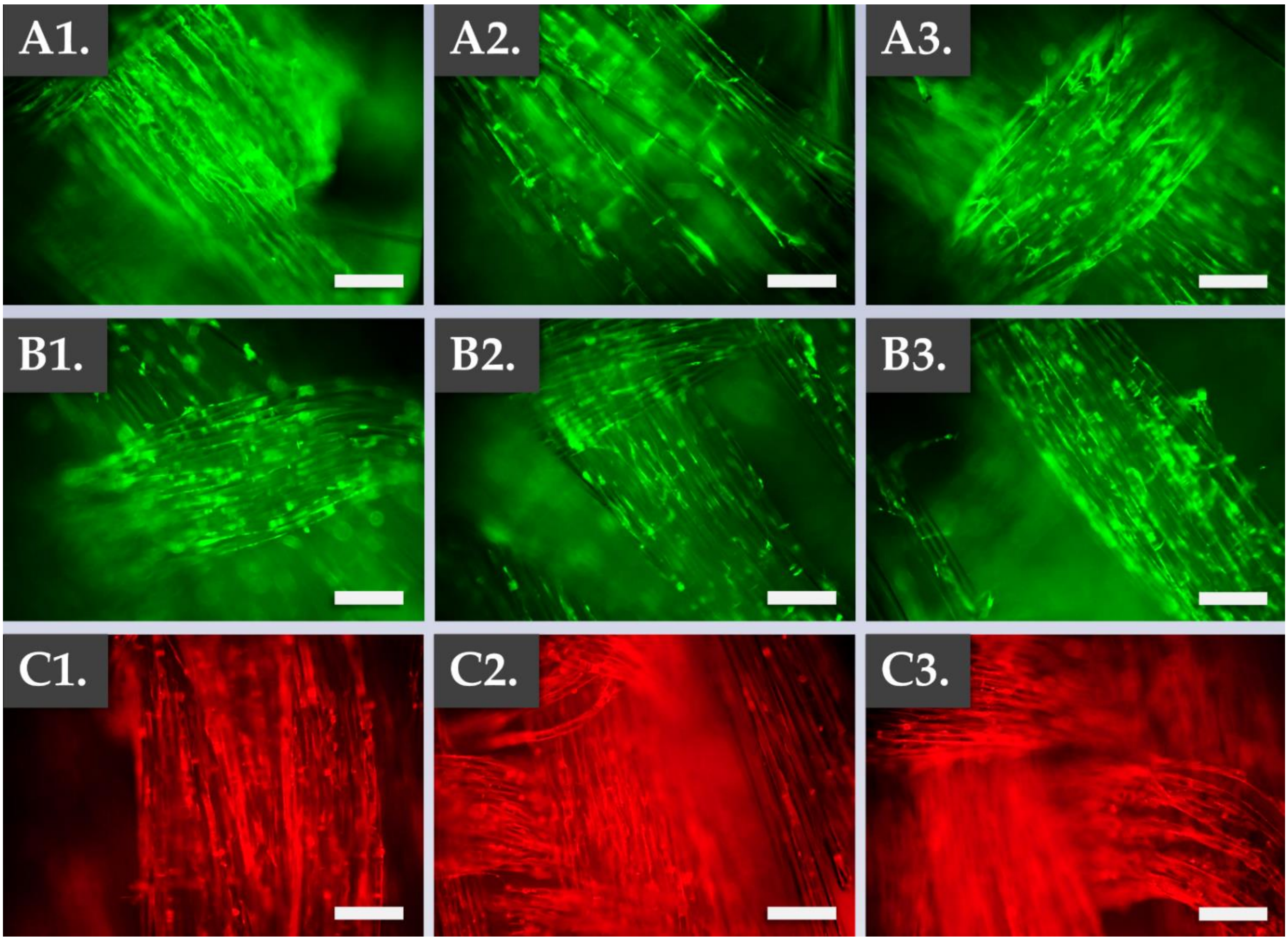

Figure S18. Immunofluorescence data excerpts showing FE002 primary progenitor extracellular matrix component synthesis and deposition along the Infinity-Lock 3 scaffold fibers in combination products incubated during seven days. The plasma-treated Infinity-Lock 3 scaffolds had been dynamically seeded with 10^5 cells/cm with FE002 primary progenitor tenocytes at passage level 7 and in cryopreserved active cellular substance form. (**A1**–**A3**) Imaging of aggrecan on a freshly harvested finished product. Scale bars = 200 µm. (**B1**–**B3**) Imaging of collagen I on a freshly harvested finished product. Scale bars = 200 µm. (**C1**–**C3**) Imaging of phalloidin on a freshly harvested finished product. Scale bars = 200 µm. Overall, the results showed a high level of structural organization of the biological materials along the scaffold fibers, confirming the potency or function of the considered FE002 primary progenitor tenocytes within the finished product.

2. Supplementary Tables

Table S1. Telomerase activity assay results [1]. Telomerase activity in the various cellular samples was investigated using a quantification kit. HeLa cells were used as a positive control. Telomerase activity was detected in all of the samples, but the respective levels of telomerase activity were found to be drastically different. Based on the ΔCT calculation method, HeLa cells displayed a telomerase activity level 225× superior in value to the considered FE002 primary progenitor tenocytes. CT, cycle threshold; qPCR, quantitative polymerase chain reaction.

Sample Type	Mean CT Values	$2^{-\Delta CT}$ Values
FE002 Primary Progenitor Tenocytes	29.70	225.71
Telomerase Detection Kit Positive Control	23.47	3.01
Internal HeLa Positive Control	21.88	1.00

[1] Telomerase activity in the various analytical samples was quantified by a qPCR method.

Table S2. CPP/KPP within the parametric and controlled process for FE002 primary progenitor tenocyte cellular active substance manufacture. For each parameter item, the predefined targets and the IPCs or PPCs are listed, along with the corresponding methods and acceptance criteria. CPP, critical process parameter; FACS, fluorescence-activated cell sorting; h, hours; IPC, in-process control; KPP, key process parameter; min, minutes; NAT, nucleic acid amplification technique; Ph. Eur., European pharmacopoeia; PPC, post-process control; QA, quality assurance; QC, quality control; RH, relative humidity.

CPP KPP	Parameter Definition	Process Targets (Cumulative)	Control Methods	IPCs PPCs	Acceptance Criteria (Cumulative)
CPP1	**Biological material identity**	Processing of the correct biological material	Operator assessment	IPC: Batch file & culture vessel label reconciliation	• Correspondence between the batch file and the culture vessel labels
CPP2	**Cell adherence for in vitro monolayer culture**	Adherence of ≥ 70% of seeded cells after 24 h of incubation following cell seeding	Operator assessment by contrast phase microscopy	IPC: Microscopic monitoring of monolayer cultures	• Adherence of ≥ 70% of seeded cells after 24 h of incubation following cell seeding
CPP3	**Cell proliferation during in vitro monolayer culture**	• Confirmation of positive cell confluency level evolution between medium exchanges<br>• Confirmation of proliferative cellular behavior in culture	Operator assessment by contrast phase microscopy, photographic recording	IPC: Microscopic monitoring at medium exchange, photographic recording of cultures	• Confirmation of proliferative cellular behavior in culture<br>• (Confluency value at medium exchange X) ≥ (Confluency value at medium exchange X-1)
CPP4	**Identity/purity of the cultured cell population**	• Specific cellular morphology & behavior maintenance in monolayer culture<br>• Absence of multiple cell populations in monolayer culture	Operator assessment by contrast phase microscopy, photographic recording, NAT	IPC (*redundant with CPP3*): Microscopic monitoring during monolayer expansion, photographic recording of cultures<br>PPC: Functional/Identity QC	• Spindle-shaped cells; distinctive fibroblastic cellular phenotype in culture<br>• Absence of multiple observable cell populations in culture<br>• Absence of multiple cell populations in FACS
CPP5	**Sterility of in vitro cell culture conditions / Processing**	No contaminating pathogens in the cultured biological materials & in the processing environment	Bactec (culture medium & monitoring plates)	PPC: Microbiological testing & monitoring	• Absence of detection for specified and non-specified contaminants or values of detection < to specified thresholds
CPP6	**Cell viability after endpoint enzymatic harvest**	Cellular viability ≥ 80% upon endpoint enzymatic harvest	Ph. Eur. 2.7.29 [1]	IPC: Total & viable cell enumeration after enzymatic harvest	• Cellular viability ≥ 80% upon enzymatic harvest

CPP7	**Sterility of the conditioned cellular active substance lot**	No contaminating pathogens in the conditioned cell vial lot & in the processing environment	Bactec (culture medium & monitoring plates), Endosafe (cell rinsing solution)	PPC: Microbiological testing & monitoring	• Absence of detection for specified and non-specified contaminants or values of detection < to specified thresholds
CPP8	**Cryopreserved material stability in storage**	• No deterioration of material primary conditioning system (cryotubes)<br>• Non-critical loss of biological material viability	Operator assessment, Ph. Eur. 2.7.29 [1]	PPC: Material inspection, qualitative & functional QCs	• No deterioration of cryotube integrity<br>• Cell viability ≥ 75% upon thawing
CPP9	**Cryogenic storage system stability**	No structural ruptures of storage system, catastrophic drop in liquid nitrogen level, or catastrophic rise in system temperature	Operator assessment & alarm systems	IPC: Storage tank temperature & level monitoring & displays<br>PPC: Storage tank data logs	• Maintenance of storage system structural integrity<br>• Storage tank temperature constantly ≤ −145 °C<br>• Nitrogen level constantly above the lower critical level
CPP10	**Documentary & material traceability**	Availability of all relevant authenticated documents & records for cell type master file, biobank inventory, and manufacturing batch files at the time of reconciliation	Operator & QA assessment	IPC/PPC: Follow-up of manufacturing data in cell type file & in biobank inventory	• Availability of all relevant authenticated documents & records for cell type master file, biobank inventory, and manufacturing batch files at the time of reconciliation
KPP1	**Rate of vial thawing for initiation from storage**	Rapid thawing of frozen vials	Operator assessment	IPC: Operator monitoring	• Complete thawing of the vials < 10 min after removal from liquid nitrogen storage and < 5 min after heating at 37 °C
KPP2	**Overall cell culture manipulation time**	Minimization of the time-period during which the cells are not in adherent state	Operator assessment	IPC: Total time the cells are processed in non-adherent state	• Total time the cells are processed in non-adherent state of < 75 min
KPP3	**Cell culture homogeneity in monolayer culture**	Homogeneous growth of the cell monolayer over the available cell culture surfaces	Operator assessment by contrast phase microscopy, photographic recording	IPC (*redundant with CPP3*): Microscopic monitoring & recording during expansion	• Absence of unpopulated cell culture surface of ≥ 20% of the total available cell culture surface in each culture vessel

KPP4	**Cell metabolism during monolayer expansion**	Maintenance of bright red to dark red colouring of the cell culture medium containing a phenol red indicator	Operator macroscopic assessment	IPC: Macroscopic monitoring of cell culture medium aspect	• Absence of orange or purple colouring of the cell culture medium or signs of severe cell starvation
KPP5	**Incubation parameter stability for cell expansion**	Maintenance of adequate incubation conditions throughout the cell culture expansions	Monitoring system records, operator assessment	IPC: Incubator monitoring & display systems<br>PPC: Incubator monitoring system data logs	• Relative limits [2] of 37 °C ± 2 °C, 5% ± 1% CO_2, 85% ± 5% RH in the incubators<br>• Absolute limits [3] of 34 °C ± 4 °C, 0% – 5% CO_2, 0% – 95% RH in the incubators
KPP6	**Total culture time-period for monolayer cell expansion**	Consistent preliminary culture times, within historical data brackets	Operator assessment	IPC: Monitoring during in vitro cell expansion	• Harvest of expanded cell population at 14 ± 3 days of culture
KPP7	**Cell confluency level at endpoint enzymatic harvest**	Maximized cell confluency level at endpoint enzymatic harvest	Operator assessment by contrast phase microscopy, photographic recording	IPC (*redundant with CPP3*): Microscopic monitoring of cultures, photographic recording of cultures	• Cell confluency level at the time of endpoint enzymatic harvest > 85% in each culture vessel
KPP8	**Cell quantity at endpoint enzymatic harvest**	Maximization of manufacturing cell yield	Ph. Eur. 2.7.29 [1]	IPC (*redundant with CPP6*): Total cell enumeration	• Cell quantity at harvest > 15 × 10^6 cells for the expanded cell population
KPP9	**Cell quantity available for conditioning of the cellular active substance lot**	Maximization of the cellular active substance lot size	Ph. Eur. 2.7.29 [1]	IPC (*redundant with CPP6*): Total cell enumeration & reconciliation	• Cell quantity at harvest > 15 × 10^6 cells for conditioning of the cellular active substance lot
KPP10	**Rate of conditioned cellular active substance lot primary freezing**	Rate of cooling of –1 °C/min from ambient temperature to –80 °C during the primary freezing phase	Operator assessment	PPC: Freezer temperature logs	• Rate of cooling of –1 °C/min from ambient temperature to –80 °C
KPP11	**Time to transfer of the cellular active substance lot to liquid nitrogen storage**	Transfer to liquid nitrogen storage after sufficient primary freezing period but < 24 h after primary freezing initiation	Operator assessment	IPC: Operator monitoring	• Transfer to liquid nitrogen storage > 4 h after primary freezing initiation but < 24 h after primary freezing initiation

KPP12	**Overall cell batch manipulation time**	Minimization of the time-period between confluent cell population harvest & conditioned cellular active substance lot cryogenic storage	Operator assessment	IPC: Total time the cells are processed before cryogenic storage	• Transfer of the cellular active substance lot to liquid nitrogen storage < 36 h after cell population harvest initiation

[1] Ph. Eur. method 2.7.29 "Nucleated cell count and viability". [2] Relative limits refer to the successive incubation phases between the cell culture initiation, the culture medium exchanges, and the cell harvest procedures (i.e., intermittent incubation time-periods characterized by closed incubator doors). [3] Absolute limits refer to the overall cell culture maintenance timeframe, from the initial culture incubation up to the cell harvest procedure (i.e., total and continuous incubation time-period, considering incubator door opening for cell culture handling).

Table S3. CPP/KPP within the parametric and controlled process for finished cytotherapeutic product manufacture. For each process item, the predefined targets and the IPCs or PPCs are listed, along with the corresponding methods and acceptance criteria. CPP, critical process parameter; h, hours; IPC, in-process control; KPP, key process parameter; NAT, nucleic acid amplification technique; Ph. Eur., European pharmacopoeia; PPC, post-process control; QA, quality assurance; QC, quality control; RH, relative humidity.

CPP KPP	Parameter Definition	Process Targets (Cumulative)	Control Methods	IPCs PPCs	Acceptance Criteria (Cumulative)
CPP1	**Biological material identity**	Processing of the correct biological material	Operator & QA assessment	IPC: Batch file & label reconciliation	• Correspondence between the batch file and the material labels
CPP2	**Cryopreserved cellular active substance stability in storage**	• No deterioration of material primary conditioning system (cryotubes)<br>• Non-critical loss of biological material quantity and viability	Operator assessment, Ph. Eur. 2.7.29 [1]	PPC: Material inspection, qualitative & functional QCs	• No deterioration of cryotube integrity<br>• Cell viability ≥ 75% upon thawing
CPP3	**Cell adherence in monolayer control plates**	Adherence of ≥ 70% of the seeded cells after 24 h of incubation following cell seeding of the control plates	Operator assessment by contrast phase microscopy	IPC: Microscopic monitoring of monolayer control plates	• Adherence of ≥ 70% of seeded cells after 24 h of incubation of monolayer controls plates following cell seeding
CPP4	**Cell proliferation in monolayer control plates**	• Confirmation of positive confluency evolution between medium exchanges<br>• Confirmation of proliferative cellular behavior in culture	Operator assessment by contrast phase microscopy, photographic recording	IPC: Microscopic monitoring at medium exchange, photographic recording of control plates	• Confirmation of proliferative cellular behavior in monolayer control plates<br>• (Confluency value at medium exchange X) ≥ (Confluency value at medium exchange X-1)
CPP5	**Sterility of in vitro cell culture conditions / Processing**	No contaminating pathogens in the cultured biological materials & in the processing environment	Bactec (culture medium & monitoring plates)	PPC: Microbiological testing & monitoring	• Absence of detection for specified and non-specified contaminants or values of detection < to specified thresholds
CPP6	**Finished product sterility**	No contaminating pathogens in the finished product	Bactec & Endosafe (culture medium & cell rinsing solution)	PPC: Microbiological testing	• Absence of detection for specified and non-specified contaminants or values of detection < to specified thresholds
CPP7	**Cell viability in the finished product**	Cellular viability ≥ 70% in the finished product	Live-Dead; MTT	IPC (*redundant with CPP6*): Viable cell enumeration & quantification	• Cellular viability ≥ 70% in the finished product

CPP8	**Cell quantity in the finished product**	Cell quantity in the finished product ≥ seeded cell quantity	Live-Dead; Cell-Titer Glo	IPC (*redundant with CPP6*): Total cell quantification	• Cell quantity in the finished product ≥ seeded cell quantity
CPP9	**Finished product uniformity**	Uniformity of the conditioned finished product	Operator assessment; MTT	PPC: Qualitative QC	• Overall and detailed appreciation within historical data brackets
CPP10	**Documentary & material traceability**	Availability of all relevant authenticated documents & records for cell type master file, biobank inventory, and manufacturing batch files at the time of reconciliation	Operator & QA assessment	IPC/PPC: Follow-up of manufacturing data in finished product file	• Availability of all relevant authenticated documents & records for finished product master file at the time of reconciliation
KPP1	**Rate of vial thawing for initiation from storage**	Rapid thawing of frozen vials	Operator assessment	IPC: Operator monitoring	• Complete thawing of the vials < 10 min after removal from liquid nitrogen storage and < 5 min after incubation at 37 °C
KPP2	**Cell metabolism during finished product incubation**	Maintenance of bright red to dark red colouring of the culture medium containing a phenol red indicator	Operator macroscopic assessment	IPC: Macroscopic monitoring of culture medium aspect	• Absence of orange or purple colouring of the culture medium or signs of severe cell starvation
KPP3	**Incubation parameter stability for finished product incubation**	Maintenance of adequate incubation conditions throughout the finished product incubation phase	Monitoring system records, operator assessment	IPC: Incubator monitoring & display systems<br>PPC: Incubator monitoring system data logs	• Relative limits [2] of 37 °C ± 2 °C, 5% ± 1% CO_2, 85% ± 5% RH in the incubators<br>• Absolute limits [3] of 34 °C ± 4 °C, 0% – 5% CO_2, 0% – 95% RH in the incubators
KPP4	**Total time-period for finished product incubation**	Consistent incubation times, within historical data brackets	Operator assessment	IPC: Monitoring during finished product incubation	• Harvest of finished products at 6 ± 2 days of incubation
KPP5	**Finished product storage temperature stability**	Stability of the finished product transport & storage temperatures	Operator & QA assessment	PPC: Transport container temperature logs	• Stable transport container temperature at 15–25 °C

[1] Ph. Eur. method 2.7.29 ”Nucleated cell count and viability”. [2] Relative limits refer to the successive incubation phases between the initiation, the culture medium exchanges, and the finished product harvest procedures (i.e., intermittent incubation time-periods characterized by closed incubator doors). [3] Absolute limits refer to the overall incubation maintenance timeframe, from the initial incubation up to the finished product harvest procedure (i.e., total and continuous incubation time-period, considering incubator door opening for cell culture handling).

Table S4. Established cryopreserved cellular active substance (i.e., FE002 primary progenitor tenocytes) quality attributes, which were specified as major KQA or as CQA. API, active pharmaceutical ingredient; CQA, critical quality attribute; EU, endotoxin units; KQA, key quality attribute.

**API Quality Attributes**	**Quality Attribute Type**	**Requirements for a Cellular API Lot** (Cumulative)
• **Cellular API Identity**	CQA	Appropriate cellular morphology and behavior in two-dimensional culture; appropriate specific proteomic markers.
• **Cellular API Purity**	KQA	Appropriate cellular morphology and behavior in two-dimensional culture; appropriate cell surface markers.
• **Cellular API Function**	CQA	Appropriate extracellular matrix synthesis & deposition in three-dimensional culture.
• **Sterility (bacteria and fungi)**	CQA	Absence of detection for specified and non-specified contaminants or values of detection < to specified thresholds.
• **Sterility (mycoplasma)**	CQA	Absence of detection for specified and non-specified contaminants.
• **Acceptable Endotoxin Level**	CQA	Endotoxin level < 0.2 EU/mL.
• **Cellular Viability Maintenance**	CQA	Cellular viability maintenance throughout storage resulting in cellular viability of > 75% upon initiation from storage.
• **Cellular Proliferation Capacity Maintenance**	CQA	Maintained potential for in vitro monolayer adherence and expansion.
• **Number of Cells/API Container**	KQA	Specified number of cells/container ± 20%.
• **API Identification**	CQA	Correct labelling of API packaging materials.
• **Appropriate API Storage**	CQA	Cryogenic API storage at temperatures constantly < –145 °C.
• **Appropriate API Validity**	CQA	Use of the API within the validated API validity period.

Table S5. Established finished product (i.e., FE002 primary progenitor tenocyte-seeded Infinity-Lock 3 construct) quality attributes, which were specified as major KQA or as CQA. API, active pharmaceutical ingredient; CQA, critical quality attribute; EU, endotoxin units; KQA, key quality attribute.

**Finished Product Quality Attributes**	**Quality Attribute Type**	**Requirements for a Finished Product Lot** (Cumulative)
• **Finished Product Identity**	**CQA**	Appropriate traceability for formulation of the correct API in the finished product.
• **Finished Product Structure**	**CQA**	Maintenance of original & appropriate 3D structure of the construct.
• **Sterility (bacteria and fungi)**	**CQA**	Absence of detection for specified and non-specified contaminants or values of detection < to specified thresholds.
• **Sterility (mycoplasma)**	**CQA**	Absence of detection for specified and non-specified contaminants.
• **Acceptable Endotoxin Level**	**CQA**	Endotoxin level < 0.2 EU/mL.
• **Cellular Viability Maintenance**	**CQA**	Cellular viability > 70% in the finished product during the whole validated product validity period.
• **Finished Product Quantity**	**KQA**	Appropriate finished product quantity at the time of reconciliation with the medical prescription.
• **Finished Product Administration System**	**CQA**	Appropriate administration system for the planned surgical operation, as prescribed.
• **Finished Product Identification**	**CQA**	Correct labelling of product packaging materials and product primary container.
• **Appropriate Finished Product Storage**	**CQA**	Ambient temperature storage of the finished product.
• **Appropriate Finished Product Validity**	**CQA**	Use of the finished product within the validated product validity period.

Table S6. Synoptic overview of preclinical in vivo work reported for the Leeds-Keio ligament (L-K), demonstrating the body of knowledge and the extensive preclinical hindsight available for this type of synthetic construct. Specifically, the Infinity-Lock 3 system is an adaptation of the L-K ligament. L-K ligaments have notably been evaluated and validated in porcine and in canine in vivo models. ACL, anterior cruciate ligament.

Author (*Year*)	Device	In Vivo Model	N° of Animals	Follow-up Periods	Analysis	Results & Comments
Seedhom et al. (*1984*)	L-K ACL	Porcine	5	1–17 months	Macroscopic assessment & histology	Histology results confirmed that host tissue covered the implanted scaffold. Collagen-yielding fibroblasts surrounded the scaffold filaments, being very cellular near the filaments and more mature at a distance. Foreign body cells were associated with the polyester material but there was no active inflammation.
Matsumoto et al. (*1984*) Fujikawa et al. (*1993*)	L-K ACL	Canine	16	36 weeks	Macroscopic assessment & histology	After 36 weeks, the implanted constructs looked almost like normal ligament tissue. The cells had acquired spindle shapes and alignment along the fibers. In the 3 cases where the constructs were intentionally not tensioned correctly, the tissue did not mature.
Otani (*1992*)	L-K ACL	Canine	60	24 weeks	Macroscopic assessment & histology	After removal of the tibial ACL stump, the ingrown tissue regenerated the enthesis (i.e., junction) of the reconstructed ligament. This was recorded only when the construct was implanted under tension (i.e., not slack).
Kawakubo (*1992*)	L-K ACL	Canine	56	24 weeks	Macroscopic assessment & histology	Regardless of the additional steps in the technique to enhance tissue ingrowth, the latter was observed under all conditions. This was however only recorded when the construct was implanted under tension and did not occur when the device was implanted with some slack.

Table S7. Synoptic overview of clinical work reported for the Leeds-Keio ligament (L-K), demonstrating the body of knowledge and the extensive clinical hindsight available for this type of synthetic construct. Specifically, synthetic L-K ligaments were developed several decades ago and have been surgically used for various indications of ligament and tendon repair. In long-term follow-up studies, a risk of re-rupture was identified, where the rate of re-rupture was found to be variable between the different studies. Based on the available animal preclinical data and on the different outcomes reported by surgeons, it is established that the surgical implantation technique, the rehabilitation protocol, and patient selection play important roles in the overall clinical outcome. ACL, anterior cruciate ligament; vs, versus.

**Author (*Year*)**	**Device**	**Surgery**	**Number of Patients**	**Study Duration**	**Results & Observations** [1]
Fujikawa (*1989*)	L-K	ACL reconstruction	42	24 months	Between 3–24 months, tissue ingrowth on the construct could be observed and the synthetic tissue matured over time to resemble native tendon. No signs of inflammation were evidenced. In some cases (i.e., 5 out of 42), new tissue formation was of poor quality. In 2 cases, the constructs were slack instead of tense.
Schroven (*1994*)	L-K	ACL reconstruction	68	5 years	Physical evaluation showed increased laxity of the constructs over time. An elevated re-tear rate of 47% was recorded.
Maracacci (*1996*)	L-K	ACL reconstruction	40	> 5 years	Sufficient stability of the constructs was observed in 80% of patients. 3 cases of re-rupture during sport activities were recorded. Outcome assessment using the IKDC score revealed 54% satisfaction. Outcome assessment using the Lysholm score revealed 80% success rates.
Meins (*1996*)	L-K	ACL	200	3 years	92% patient satisfaction rate. 89% of patients had good functional scores. Elongation, partial rupture, or rupture rate: 11% of patients.

Fukuta (*2002*)	L-K	Patellar tendon reconstruction	2	36 & 40 months	Case 1: 75 years old male patient. Excellent quadriceps strength. No pain. Hospital surgery knee-score of 86. Case 2: 72 years old female patient. Good quadriceps strength. Hospital surgery knee-score of 74.
Jones (*2007*)	L-K	ACL reconstruction	50	> 10 years	IKDC score: 92% high satisfaction. Knee stability: 84 % of patients. At 1 year, 68% of patients returned to pre-injury sport levels. Only 8% of patients reported knee functional decline. At 10 years: 40% of patients still performed sport activity levels similar to pre-injury state. The recorded reductions in sport activity were mainly due to lifestyle changes.
Zaffagnini (*2008*)	L-K	ACL reconstruction	1	20 years	The construct was completely covered with collagen fibers. Low-level vascular network was observed.
Ghalayini (*2009*)	L-K vs. autogenous patellar tendon graft (PT)	ACL reconstruction	26 PT vs. 24 L-K	5 years	No statistical difference found at 5 years in IKDC, Lysholm, and one-hope test scores. Activity and function scores improved over time. Improvement was faster with the autogenous graft. Confirmation that the synthetic construct is an option for tendon repair.
Abdullah (*2021*)	Orthotape	Hand reconstruction surgery (extensor and flexor tendons)	9	> 3 years	5 patients lived with the constructs > 5 years. A 22% extrusion rate was recorded.

[1] Discrepancies between the different scoring methodologies were noted. Subjective evaluations (i.e., patient-reported) were generally more favorable than clinical evaluations with parameters like tendon laxity.

Table S8. In addition to the retrospective preclinical and clinical assessments available on the synthetic constructs, prospective clinical studies are underway. Specifically, the device manufacturer has submitted three post-market clinical follow-up studies for various devices (i.e., the Jewel ACL, AchilloCordPLUS system, and the Poly-Tape) similar to the Infinity-Lock 3 system (i.e., CE-marked medical device polyester scaffolds). ACL, anterior cruciate ligament; vs, versus.

Study Number	Study Title	Device	Status	Description	Outcomes
NCT04580290	Jewel ACL post-market clinical follow-up study	Jewel ACL	Completed	Assessment of re-rupture rates and patient outcomes from subjects with at least 5 years of follow-up, treated with the Jewel ACL device for ACL reconstruction. The impact of autograft augmentation was also assessed. Groups: Jewel ACL only vs. Jewel ACL+ autograft.	Jewel ACL only: 88 patients, 5.7 % re-rupture. Jewel ACL+ autograft: 32 patients, 3.1% re-rupture.
NCT05304819	Post-market clinical follow-up study of the AchilloCordPLUS implant set for acute Achilles tendon repair	AchilloCordPLUS	Recruiting	55 subjects to be enrolled. Follow-up is at 2, 4, 6, 8 and 12 weeks, 6 months, 1 and 2 years. Outcomes to be measured:<br>➢ Achilles tendon rupture<br>➢ Range of motion<br>➢ Return to work and sports<br>➢ Tegner activity scale	Ongoing study
NCT05264389	Post-market clinical follow-up study of the Poly-Tape device for medical patellofemoral ligament reconstruction	Poly-Tape	Recruiting	55 subjects to be enrolled. Follow-up is at 6 months, 1 year, 2 years, and 5 years, in the clinic and by questionnaires. Outcomes to be measured:<br>➢ Re-dislocation/subluxation score at 1 year<br>➢ Kujala score at 1 year<br>➢ Range of motion<br>➢ Patient reported quality of life<br>➢ Norwich patellar instability score	Ongoing study

Table S9. Clinical trials relative to cell-based tendon regenerative medicine, as registered on the ClinicalTrials.gov registry and in the Australian New Zealand Clinical Trials Registry. ADRC, adipose-derived regenerative cells; ASC, adipose-derived stem cells; BM-MSC, bone marrow-derived mesenchymal stem cells; MSC, mesenchymal stem cells; NA, not applicable; vs, versus; PTRCT, partial-thickness rotator cuff tears.

Study Reference Number	Intervention	Study/Clinical Intervention Description
NCT01687777	OrthADAPT ± MSC	Supraspinatus tendon surgery ± MSC.
ACTRN12617000684325	Ortho-ATI vs. corticosteroids	Autologous tenocyte injection for rotator cuff repair.
ACTRN12616000458437	Ortho-ATI vs. surgery	Autologous tenocyte injection for severe, chronic, resistant lateral epicondylitis.
NCT01856140	ALLO-ASC	Allogeneic MSC ultrasonographic-guided injection in epicondylitis.
NCT04670302	ASC+ AAdMSC-HAM	ASC seeded on HAM scaffold. Investigation in supraspinatus tendon repair augmentation.
NCT03449082	ALLO-ASC	Intra-tendon injection of allogeneic ASC in intractable common extensor tendinosis patients in comparison with a control treatment.
NCT03068988	BM-MSC	BM-MSC for healing of sutured tendon supraspinatus muscle.

NCT03279796	ASC vs. betamethasone	Injection in rotator cuff or in lateral epicondylosis.
NCT03362424	MSC	Investigation of MSC in rotator cuff repair.
NCT02484950	BM-MSC	Investigation of BM-MSC in sutured rotator cuff repair.
NCT03838666	MSC	Investigation of MSC in rotator cuff repair surgery.
NCT04057833	E-CEL UVEC (allogeneic)	E-CEL UVEC cells in full-thickness rotator cuff tears with arthroscopic surgical repair.
NCT04077190	ASC vs. corticosteroids	ADRCs for PTRCT compared to a single corticosteroid injection into the associated subacromial space.
NCT05400798	ASC vs. corticosteroids	Follow-up study.
